# Whole-Genome Resequencing of Red Junglefowl and Indigenous Village Chicken Reveal New Insights on the Genome Dynamics of the Species

**DOI:** 10.3389/fgene.2018.00264

**Published:** 2018-07-20

**Authors:** Raman A. Lawal, Raed M. Al-Atiyat, Riyadh S. Aljumaah, Pradeepa Silva, Joram M. Mwacharo, Olivier Hanotte

**Affiliations:** ^1^Cells, Organisms and Molecular Genetics, School of Life Sciences, University of Nottingham, Nottingham, United Kingdom; ^2^Genetics and Biotechnology, Animal Science Department, Agriculture Faculty, Mutah University, Karak, Jordan; ^3^Animal Production Department, College of Food and Agriculture Sciences, King Saud University, Riyadh, Saudi Arabia; ^4^Department of Animal Sciences, Faculty of Agriculture, University of Peradeniya, Peradeniya, Sri Lanka; ^5^Small Ruminant Genomics, International Centre for Agricultural Research in the Dry Areas, Addis Ababa, Ethiopia; ^6^LiveGene – CTLGH, International Livestock Research Institute, Addis Ababa, Ethiopia

**Keywords:** red junglefowl, *Gallus gallus*, indigenous village chicken, chicken domestication, chicken adaptation, environmental adaptation, positive selection, candidate sweep regions

## Abstract

The red junglefowl *Gallus gallus* is the main progenitor of domestic chicken, the commonest livestock species, outnumbering humans by an approximate ratio of six to one. The genetic control for production traits have been well studied in commercial chicken, but the selection pressures underlying unique adaptation and production to local environments remain largely unknown in indigenous village chicken. Likewise, the genome regions under positive selection in the wild red junglefowl remain untapped. Here, using the pool heterozygosity approach, we analyzed indigenous village chicken populations from Ethiopia, Saudi Arabia, and Sri Lanka, alongside six red junglefowl, for signatures of positive selection across the autosomes. Two red junglefowl candidate selected regions were shared with all domestic chicken populations. Four candidates sweep regions, unique to and shared among all indigenous domestic chicken, were detected. Only one region includes annotated genes (*TSHR* and *GTF2A1*). Candidate regions that were unique to each domestic chicken population with functions relating to adaptation to temperature gradient, production, reproduction and immunity were identified. Our results provide new insights on the consequence of the selection pressures that followed domestication on the genome landscape of the domestic village chicken.

## Introduction

Since Charles Darwin proposed a single ancestry of chicken from the red junglefowl, its status as either monophyletic or polyphyletic has been debated (Darwin, 1868; Beebe, 1918; Danforth, 1958; Morejohn, 1968; Fumihito et al., 1994). While the red junglefowl is the main ancestor, some studies are now supporting genetic contributions from other junglefowl species (Eriksson et al., 2008; Lawal, 2017).

Evidences are also controversial as to the timing and places where chicken domestication first occurred (Zeuner, 1963; Crawford, 1984; West and Zhou, 1988; Fumihito et al., 1996; Liu et al., 2006; Xiang et al., 2014, 2015; Peters et al., 2015). A study on mitochondrial DNA suggests multiple centers of chicken domestication (Liu et al., 2006) from which chicken dispersed to different parts of the world through humans' influence. They entered North Africa, the Middle East and Sri Lanka from the Indian subcontinent, while maritime introductions, likely originating initially in South-East Asia, occurred along the coast of East Africa as well as Sri Lanka (Silva et al., 2009; Gifford-Gonzalez and Hanotte, 2011; Mwacharo et al., 2011). Following these migration events, natural and artificial selections have shaped the genome landscape of domestic chicken resulting in a wide spectrum of breeds and ecotypes.

Aside the fancy breeds, domestic chickens primarily come under two major categories; commercial and indigenous village chickens (Schmid et al., 2015). In developing countries, the latter play prominent roles in the livelihood of smallholder farmers, being adapted to their local environmental conditions. They are often under the custody of women and children, mainly kept as dual purpose (eggs and meat) birds. Furthermore, indigenous village chicken showing special visual appeal such as comb type, skin and feather colors may have been selected by smallholder farmers, thereby increasing the frequencies of desirable phenotypes (Dana et al., 2010; Desta et al., 2013). Extensive phenotypic variations such as plumage color and other morphological characteristics, behavioral, and production traits, which are present in domestic chicken but absent in the red junglefowl, are the result of domestication, adaptation to various agro-ecosystems and stringent human selection for production and/or aesthetic values (Schütz et al., 2001; Keeling et al., 2004; Tixier-Boichard et al., 2011).

In commercial chicken lines, the genetic factors that control growth, development, reproduction, and production traits have been well studied (Rubin et al., 2010; Fu et al., 2016). Meanwhile, the genetic mechanisms underlying unique adaptations to tropical environmental pressures and productivity remain poorly studied in indigenous chicken. Likewise, in the red junglefowl, little is known about the genetic control of its adaptation and survival in its natural habitat. Here, we investigate, using whole-genome sequence data, footprints of positive selection in the genome of red junglefowl and domesticated indigenous village chicken in order to better understand the evolutionary pressures during the domestication of the species and its adaptation to different production environments.

## Materials and methods

### Sampling and sequencing

A total of 27 indigenous domestic village chickens were sampled and then grouped into three populations based on the countries of origin. They include, Ethiopian domestic chicken from two districts, Horro (*n* = 6, altitude around 2,320 m above sea level (asl)) and Jarso (*n* = 5, altitude of around 1,870 m asl), Saudi Arabian domestic chicken from three villages, Al Qurin (*n* = 2, altitude around 130 m asl), Goligglah (*n* = 2, altitude around 130 m asl) and Al Oyoun (*n* = 1, altitude around 110 m asl) in the Eastern Province, and Sri Lankan domestic chicken from Puttalam district (*n* = 11, altitude around 60 m asl). Horro is a sub-humid region, with an annual rainfall of 1,685 mm and an average temperature of around 19°C. Jarso is semi-arid with an average annual temperature of 21°C and annual average rainfall of 700 mm (Desta et al., 2013). The Eastern Province of Saudi Arabia has an average annual temperature of 26°C (ranging from 21.2 to 50.8°C) and average annual rainfall of 74 mm. Puttalam district of Sri Lanka has an average annual rainfall of ~1,000 mm and temperature of 27°C.

Collection of blood samples was through the wing vein and genomic DNA was extracted using ammonium acetate precipitation (Bruford et al., 1998) and phenol-chloroform protocols. A minimum of 3 μg at 30 ng/μl DNA concentration was used for whole genome re-sequencing. Samples were sequenced at the Beijing Genomic Institute (BGI) or at Novogene on a HiSeq 2000/2500 Illumina platform. Five hundred (500) bp paired-end insert size libraries with read lengths of between 90–100 bp and genome coverage of between 10X and 30X (Table S1) were generated. Adapter pollutions from the raw reads and sequences with quality scores ≤ 5 were deleted at source BGI/Novogene.

For the six red junglefowl, one whole-genome sequence (15X genome coverage) from a captive bird (Koen Vanmechelen private collection)^1^ and five whole genome sequences (12X−36X genome coverage) from the Wang et al. (2015) were included in the analyses (Table S1). The five red junglefowl were sampled in Yunnan (altitude ~3,000 m asl) and Hainan (altitude ~1,840 m asl) provinces, China. Yunnan is a subtropical highland or humid tropical zone with an annual rainfall range of between 600 mm and 2,300 mm, and annual temperature range of between 8 to 27°C. For the humid tropical Hainan province, the average annual rainfall is about 2,000 mm and temperature ranges between 16 and 29°C. Fastq files for all samples newly sequenced in this study have been deposited to NCBI with the SRA accession number SRP142580 or accessible through https://www.ncbi.nlm.nih.gov/sra/SRP142580.

### Sequence alignment and variants calling

The 33 whole-genome sequences were independently aligned to *Galgal* 4.0, which has reference genome size of 1.07 Gb (Hillier et al., 2004), using Burrows-Wheeler Aligner (BWA) version 0.7.5a (Li and Durbin, 2010). Sorting the alignment files into coordinate order, marking the duplicate reads and indexing the binary alignment map (bam) files were done using Picard tools version 1.105^2^. Using the genome analysis toolkit (GATK) version 3.4.0 (McKenna et al., 2010; DePristo et al., 2011; Auwera et al., 2013), we performed a two-steps protocol for local realignment around insertions and deletions (indels) to clean up artifacts that arose, during the initial mapping steps, following misalignments. Finally, we applied a quality score recalibration step for each base call to remove any errors carried over during the sequencing.

To call variants, we ran “HaplotypeCaller” from GATK for each sample bam file to create a single-sample “gVCF” using the “-emitRefConfidence GVC” option. We then followed the multi-sample aggregation approach which jointly genotyped variants by merging together, records of all genome data from each population. Using the “-selectType SNP” option along with the “SelectVariants” from GATK, we extracted the SNPs from the raw genotype file before filtering the extracted SNPs using “VariantFiltration.” All investigations were restricted to bi-allelic single nucleotide polymorphisms (SNPs) using bcftools version 1.2 (Li et al., 2009), autosomes (chromosomes 1–28) and the full mitochondrial DNA (mtDNA).

The mapping metrics including the percentage of read pairs that properly mapped to the same chromosome, mean depth coverage, total reads mapped, percentage of the genome with bases covered by at least 5, 10 and 20 reads were calculated using samtools version 0.1.19 (Li et al., 2009). Using Ensembl's “VEP” version 85 (Aken et al., 2016), we predicted the consequences of the variants while the total number of SNPs in each sample/population were identified using VCFtools version 0.1.11 (Danecek et al., 2011). The “VennDiagram” package (Chen and Boutros, 2011) in R was used to plot the unique and shared SNPs between the domestic chicken and red junglefowl.

### Population structure and genetic differentiation

We removed SNPs in linkage disequilibrium to establish the genetic structure of each population and the relationships between samples using PLINK version 1.9^3^. We then assessed the structure of each population unsupervised, using ADMIXTURE version 1.3.0 (Alexander et al., 2009). Using the default (folds = 5) for cross-validation, we ran the analysis for 10 clusters (*K*). For the principal component analysis (PCA), we ran the smartpca program in eigenstrat version 6.0.1 (Price et al., 2006). The proportion of variance explained by each eigenvector was calculated by dividing the corresponding eigenvalue to the sum of all the eigenvalues.

Genome-wide, nucleotide diversity (π) and genetic differentiation (*F*_ST_) were calculated within and between population(s), respectively in 20 kb windows with 10 kb slide using VCFtools version 0.1.11 (Danecek et al., 2011). For *F*_ST_, the pairwise values were calculated between each domestic chicken population and the red junglefowl.

### Mitochondrial DNA analysis

The full mitochondrial consensus sequence was extracted from the whole genome sequence of each of the 33 samples using “consensus” option in bcftools version 1.2 (Li et al., 2009). Multiple sequence alignment was conducted for the 33 mtDNA genomes using ClustalX version 2.1 (Larkin et al., 2007). To identify the best-fit nucleotide substitution model, we ran jModeltest version 2.1.7 (Darriba et al., 2012). The HKY+I+G model (Hasegawa et al., 1985) was selected as the best, based on the Akaike Information Criterion (AIC), and was subsequently used to construct an unrooted maximum likelihood tree using phyml 3.0 (Guindon and Gascuel, 2003). The tree was then viewed in MEGA 7.0 (Kumar et al., 2016).

To assess the haplogroup (clade) of each mtDNA sequence, we extracted the first 397 bp hypervariable region (HVR) of the D-loop from the full mitochondrial sequences using as reference mtDNA sequences of Komiyama et al. (2003) (NCBI accession number AB098668) and six haplogroups *sensu* Mwacharo et al. (2011) (Table S2). A haplotype data file including all the 40 HVR of D-loop sequences was generated using DnaSP version 5.1 (Librado and Rozas, 2009) from which the median-joining network was constructed using network 5.0.0.1^4^

### Selective sweep analysis

To detect putative selection sweeps, we used the pool heterozygosity (*H*_p_) method (Rubin et al., 2010). It was performed using a 20 kb window size with a 10-kb sliding step following the equation:

(1) $$\begin{array}{r} H\text{p}=\;\frac{\;2\sum n_{MAJ}\sum n_{MIN}\;}{{\left(\sum n_{MAJ}+\sum n_{MIN}\right)}^{2}} \end{array}$$

Where ∑*n*_*MAJ*_ and ∑*n*_*MIN*_ are the sums of major and minor allele frequencies, respectively for all the SNPs in the 20 kb window. The values for the *H*_p_ calculated for each window size were then Z-transformed using the equation:

(2) $$\begin{array}{r} Z\left(H\text{p}\right)=\;\frac{\;H\text{p }-\bar{X}\left(H\text{p}\right)\;}{\sigma \;\left(H\text{p}\right)} \end{array}$$

Where $\bar{X}$ is the mean and, σ is the standard deviation of *H*_p_.

A genome-wide score of Z(*H*_p_) ≤ −4.0 was taken as the threshold after examining the distribution plot of the Z(*H*_p_) values (Figures S1A–D). The size of each candidate selective sweep region was calculated by adding the number of overlapping adjacent windows above the genome-wide threshold.

Since the accuracy of detecting selective sweeps depend on the number of SNPs in each window and considering the high polymorphisms identified within populations, only windows with at least 50 SNPs were considered. Following this criterion, 52, 103, 56, and 39 windows were excluded from the Ethiopian, Saudi Arabian and Sri Lankan chicken populations and red junglefowl datasets, respectively.

### Haplotype trees

In order to assess if a single or multiple haplotypes were selected across population, we build-up haplotype trees for common candidate “domesticated” regions and regions shared between all domestic chicken and red junglefowl. Only shared significant window(s) across population were used to define the region. For this purpose, we included the haplotype sequences from all junglefowl species used in Lawal (2017) study. Maximum likelihood trees were rooted with the green junglefowl and built using Phyml 3.0 (Guindon and Gascuel, 2003) after the evolutionary model was predicted using jModeltest 2.1.7 (Darriba et al., 2012). Genome sequences of the non-red junglefowl species and *G. gallus bankiva* are available at DNA Data Bank Japan Sequence Read Archive (accession no. DRA003951) (Ulfah et al., 2016).

### Remapping the *Galgal* 4.0 sweep regions to *Galgal* 5.0 coordinates

Following the release of the new reference genome *Galgal* 5.0 (Warren et al., 2017), we remapped the *Galgal* 4.0 candidate sweep regions to the corresponding *Galgal* 5.0 coordinates using NCBI remapper (February 2017 release). All the remapping options were set to default threshold. Selective sweep regions based on the *Galgal* 4.0 and their corresponding positions in *Galgal* 5.0 are reported at Tables S4–S7, including changes in the annotated genes between the two reference genomes. Only *Galgal* 5.0 position annotated genes at candidate regions are reported and discussed herein.

### Gene ontology and pathways analysis

To establish the biological significance of the genes found in each candidate selection sweep region, we performed gene ontology and pathways analysis using Database for Annotation, Visualization, and Integrated Discovery (DAVID version 6.8)^5^ and the Kyoto Encyclopaedia of Genes and Genomes (KEGG) (KOBAS version 3.0)^6^. The Fisher Exact *P* < 0.05 default threshold was used to identify over-represented genes.

## Results

### Sequencing and SNPs identification

Following filtering for quality checks and adapter pollutions, clean sequence reads for each domestic chicken sample range between 108.8 and 408.9 million base pairs (bp) depending on the extent of genome coverage (10X−30X) (see Table S1). For each domestic chicken, the number of nucleotides with quality score >20 (Q20) ranged from 94 to 96%.

More than 90% of the read pairs in all samples were properly mapped to the same chromosome. Except for the red junglefowl_koen sample with 94.69% of mapped reads, ≥97% of all the reads were mapped to the reference genome. On average, ≥97% of the bases were covered by at least 5 reads, while ≥89% of the bases had minimum support of 10 reads (Table S1).

The intermediate genomic variants generated for individual birds using the “HaplotypeCaller” from GATK (Auwera et al., 2013) were used to jointly genotype all samples belonging to a population into a single variants file. Excluding the multi-allelic sites, the average number of SNPs in each sample was ~6 million (~6 SNPs/kb). The only exception is red junglefowl5 and red junglefowl_koen samples having ≥7 million SNPs. Around 60% of the SNPs were heterozygous in each sample except in three domestic chicken (JB1A25B, JB2A04B, and Saudi Arabia1), which showed ~45% heterozygous SNPs (Table S1). At the population level, we identified 13.07 (~12 SNPs/kb), 10.23 (~9 SNPs/kb) and 14.46 (~13 SNPs/kb) million SNPs in Ethiopian, Saudi Arabian, and Sri Lankan domestic chickens, respectively, and 15.31 (~14 SNPs/kb) million SNPs in the red junglefowl. It corresponds to a total of 17.0 million SNPs (~16 SNPs/kb) for the domestic chicken populations combined, and 20.81 million SNPs (~19 SNPs/kb) after combining the genome of all the domestic chicken populations and red junglefowl (Table S3; Figure S2).

Around 11.05 million SNPs were shared between domestic chicken and red junglefowl, 5.4 and 3.8 million SNPs were unique to domestic chicken and the red junglefowl, respectively (Figure S2). We identified 1.76 million (13% of the total number of SNPs), 1.03 million (10%), and 2.33 million (16%) novel SNPs in Ethiopian, Saudi Arabian and Sri Lankan domestic chickens, respectively and 4.45 million (29%) in the red junglefowl. More than 54% of the SNPs occurred within introns, 30% in intergenic regions, 5.7 and 4.3% in upstream and downstream gene regions, respectively. 3′ and 5′ UTR variants accounted for 1.8 and 0.4% of the SNPs, respectively (Table S3).

### Population structure

**Figure 1:**
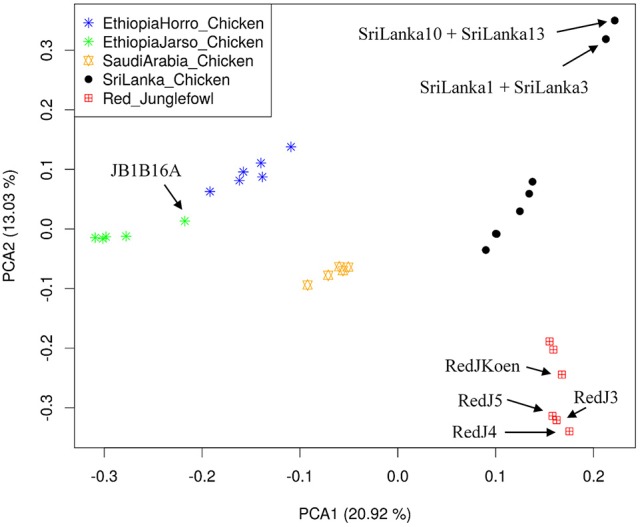
Principal Component Analysis (PCA) plot. The top left label defines colors for each population. Individuals with name annotations have been uniquely identified for comparison purpose with Figures 2, 3. The proportion of variance explained by the eigenvector in the x- and y-axes are denoted beside the PCA1 and PCA2.

**Figure 2:**
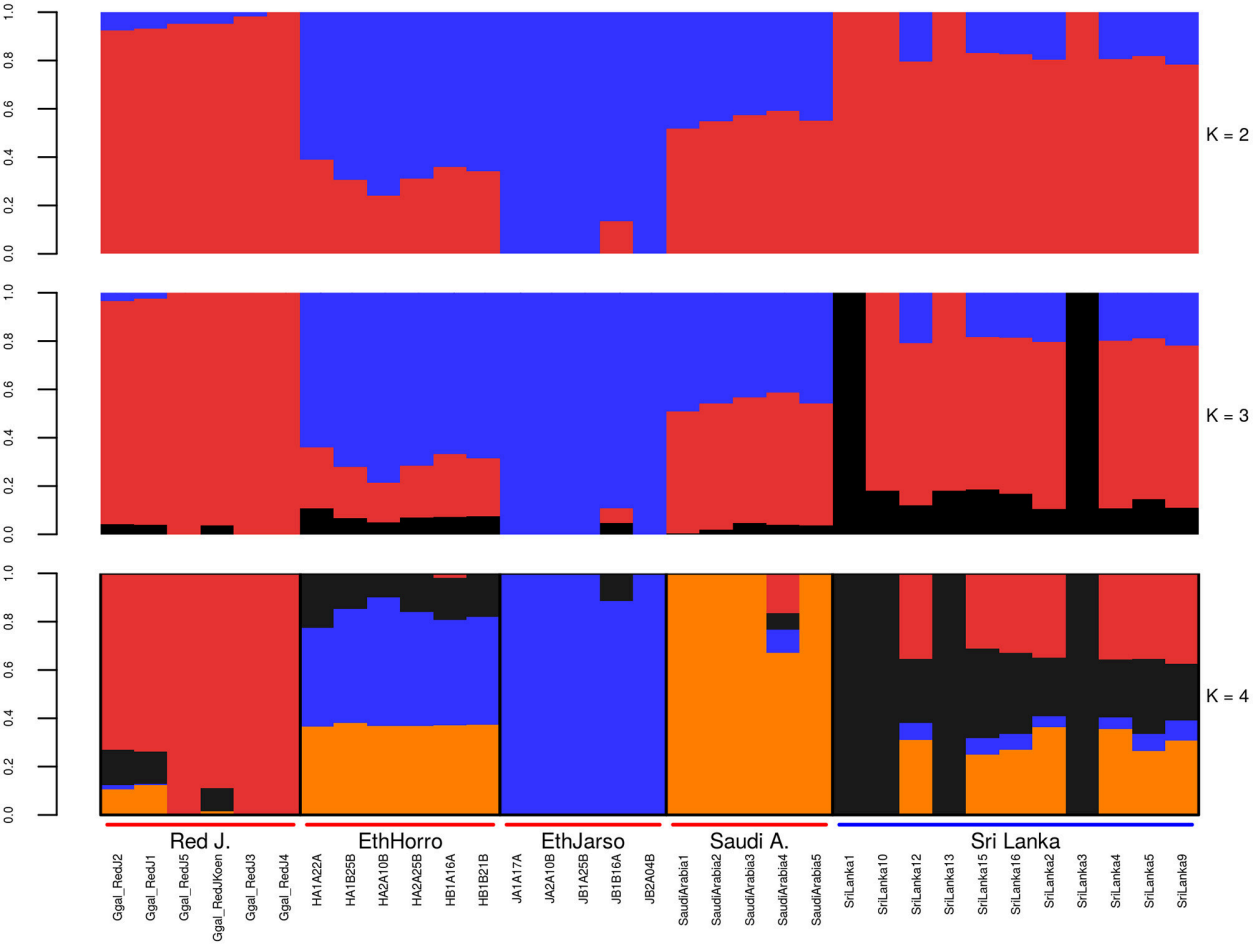
Autosomal admixture plot. The labels on the x-axis; Red J. (red junglefowl), EthHorro (Ethiopian Horro chicken) and EthJarso (Ethiopian Jarso chicken), Saudi A. (Saudi Arabian chicken), and Sri Lanka (Sri Lankan chicken). Each population is delineated with black border lines and each admixture bar is annotated with the sample names within their respective populations. Under the Red J. label, the sample names with prefix Ggal_RedJ (1, 2, 3, 4, 5, koen) correspond to red junglefowl (1, 2, 3, 4, 5, and koen) samples in Table S1.

Population structure at autosomal level was examined using Principal Component (PC) (Figure 1) and Admixture analyses (Figure 2). PC1 and PC2 separate all the domestic populations from the red junglefowl, a result that was also obtained at *K* = 4 in the admixture analysis. The other admixture plots 5 ≤ *K* ≤ 10 are shown in Figure S3.

### Diversity and genetic differentiation

Across populations, we observe the highest genome nucleotide diversity (π = 0.0052) in the red junglefowl. Among the domestic chicken populations, Sri Lankan domestic chicken show the highest nucleotide diversity (π = 0.0046), followed by the Ethiopian Horro (π = 0.0040), Saudi Arabian (π = 0.0039) and Ethiopian Jarso domestic chicken (π = 0.0036).

For the pairwise *F*_ST_ analysis, we calculated the genetic distances between the red junglefowl and each of the domestic chicken populations to evaluate the levels of autosomal genetic differentiation between domestic chicken and red junglefowl. The Ethiopian Jarso returns the highest *F*_ST_ value (0.148), followed by Ethiopian Horro (*F*_ST_ = 0.113), Saudi Arabian (*F*_ST_ = 0.095) and the Sri Lankan domestic chicken (*F*_ST_ = 0.062) populations.

### Mitochondrial phylogenetic relationships

**Figure 3:**
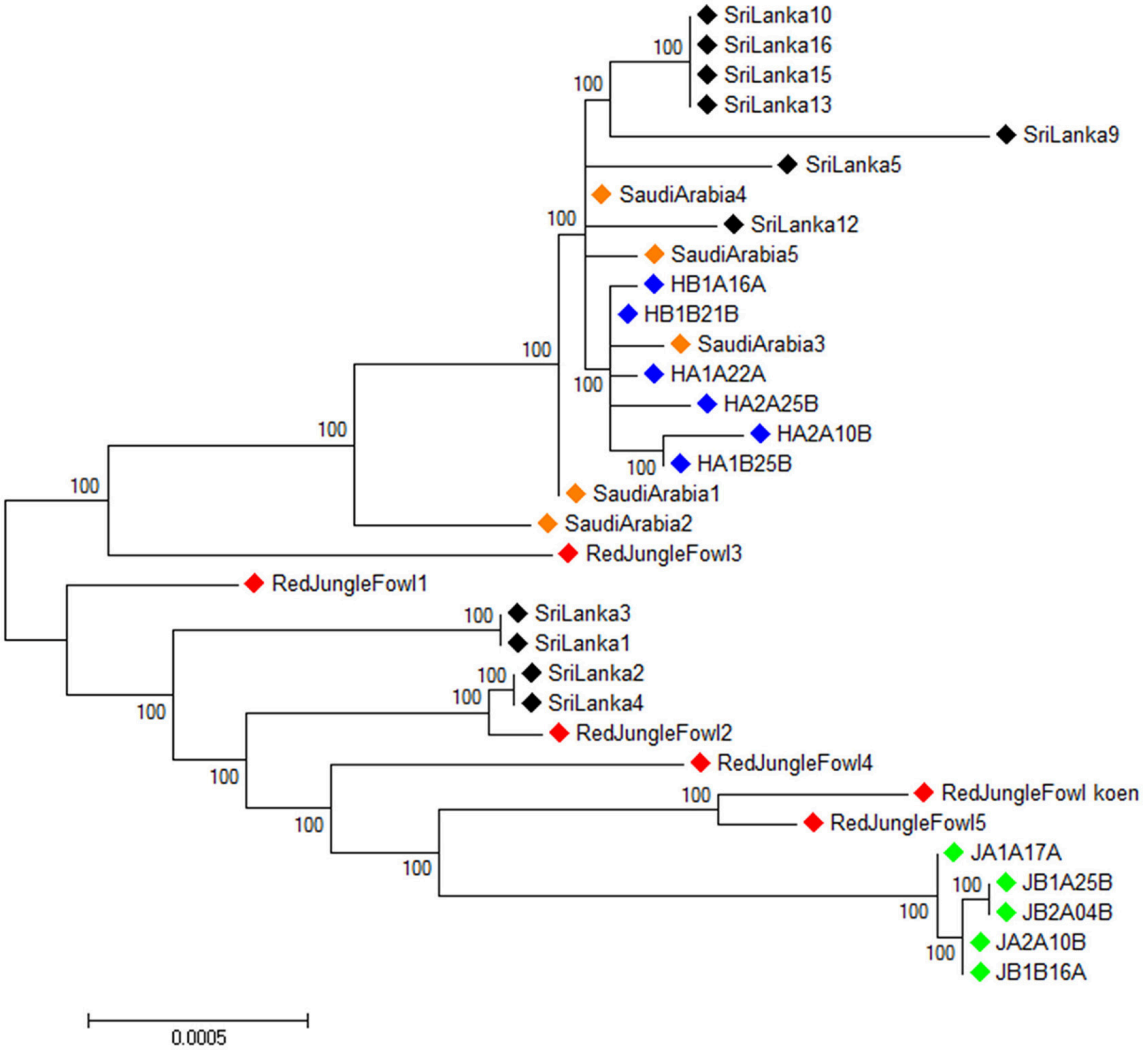
Unrooted maximum likelihood phylogenetic tree for the full mitochondrial DNA sequences of all the samples. 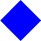, Ethiopian Horro chicken, 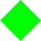, Ethiopian Jarso chicken, 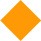, Saudi Arabian chicken, 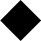, Sri Lankan chicken, 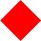, Red junglefowl.

The 33 individual mitochondrial genomes were used to construct an unrooted maximum likelihood tree using Phyml 3.0 (Guindon and Gascuel, 2003) (Figure 3). Sri Lankan domestic chicken are divided in two clusters. The first cluster belongs to the same lineage than the Ethiopian Horro and Saudi Arabian chicken. The second cluster included the red junglefowl and Ethiopian Jarso chicken with the Sri Lankan domestic chicken being closer to the former than the later.

**Figure 4:**
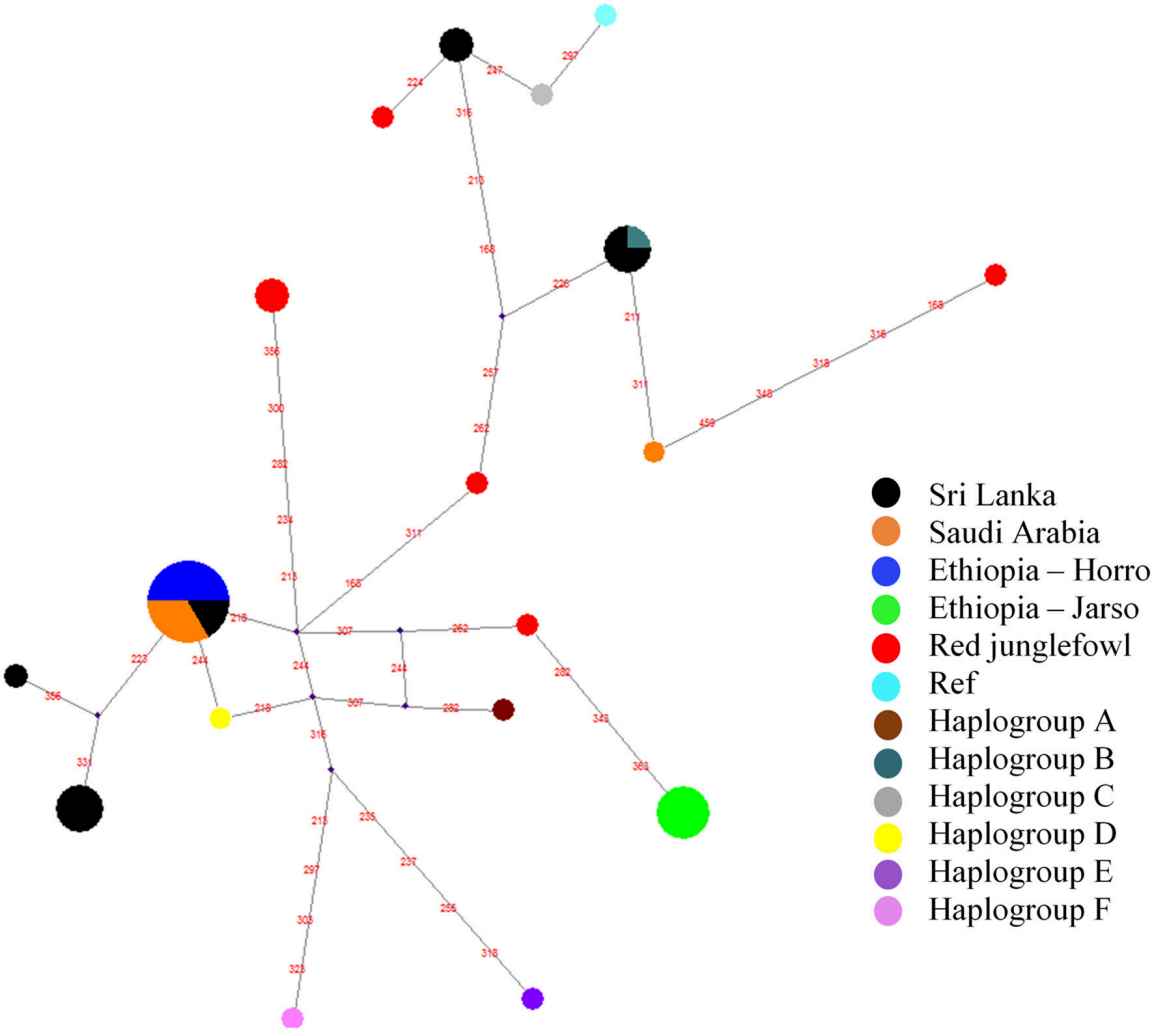
Haplotype median-joining network analysis for the hypervariable D-loop region of mitochondrial DNA. Red values on the lines represent segregating sites. The “Ref” represents the reference *Galgal* 5.0 haplotype for the D-loop region (accession number AB098668).

To assess the possible maternal origins of our indigenous village chicken mitochondrial DNA, we extracted the hypervariable region (spanning the first 397 bp) of the mitochondrial DNA D-loop region. We included in our analysis reference haplotypes representing six major chicken haplogroups *sensu* Mwacharo et al. (2011) (Table S2). Haplogroups A, B, C, and D were observed in our dataset (Figure 4). Within a single segregating site, all Ethiopian Horro, four Saudi Arabian and two Sri Lankan domestic chicken haplotypes are linked to haplogroup D. Four Sri Lankan haplotypes are separated by three mutations from the reference D haplotype. Other Sri Lankan domestic chicken haplotypes (*n* = 5) link to haplogroups B and C and a single Saudi haplotype was also close to haplogroup B. The Ethiopian Jarso chicken haplotypes were found closer to haplogroup A.

### Mean genome heterozygosity

We calculated the average level of within population *H*_p_ genome heterozygosity (20 kb window size). The genome heterozygosity of the red junglefowl averages to 0.32 ± 0.028 (*n* = 6). Among the domestic chicken populations, Ethiopian chicken population shows the lowest level of genome heterozygosity (mean 0.31 ± 0.051, n = 11) followed by Sri Lankan chicken population (0.32 ± 0.039, *n* = 11). Saudi Arabian chicken population shows the highest level of genome heterozygosity (0.36 ± 0.048, *n* = 5) (Table 1).

**Table 1:** Genome-wide pool heterozygosity (*H*_p_) statistics for the three domestic populations and red junglefowl.

**Populations**	**Sample no**	**Pool heterozygosity (*****H***_**p**_**) statistics**			
		**Total number of windows**	**Genome mean (*H*_p_)**	**Z(*H*_p_) ≤ − 4.0^a^**	**Number of candidate sweep regions identified**
Ethiopian domestic chicken	11	89,443	0.31 ± 0.051	247	84
Saudi Arabian domestic chicken	5	87,646	0.36 ± 0.048	565	212
Sri Lankan domestic chicken	11	89,701	0.32 ± 0.039	299	127
Red junglefowl	6	90,170	0.32 ± 0.028	434	190

a *Total number of windows that passed the genome-wide threshold*.

### Selection sweeps detection in red junglefowl

**Figure 5:**
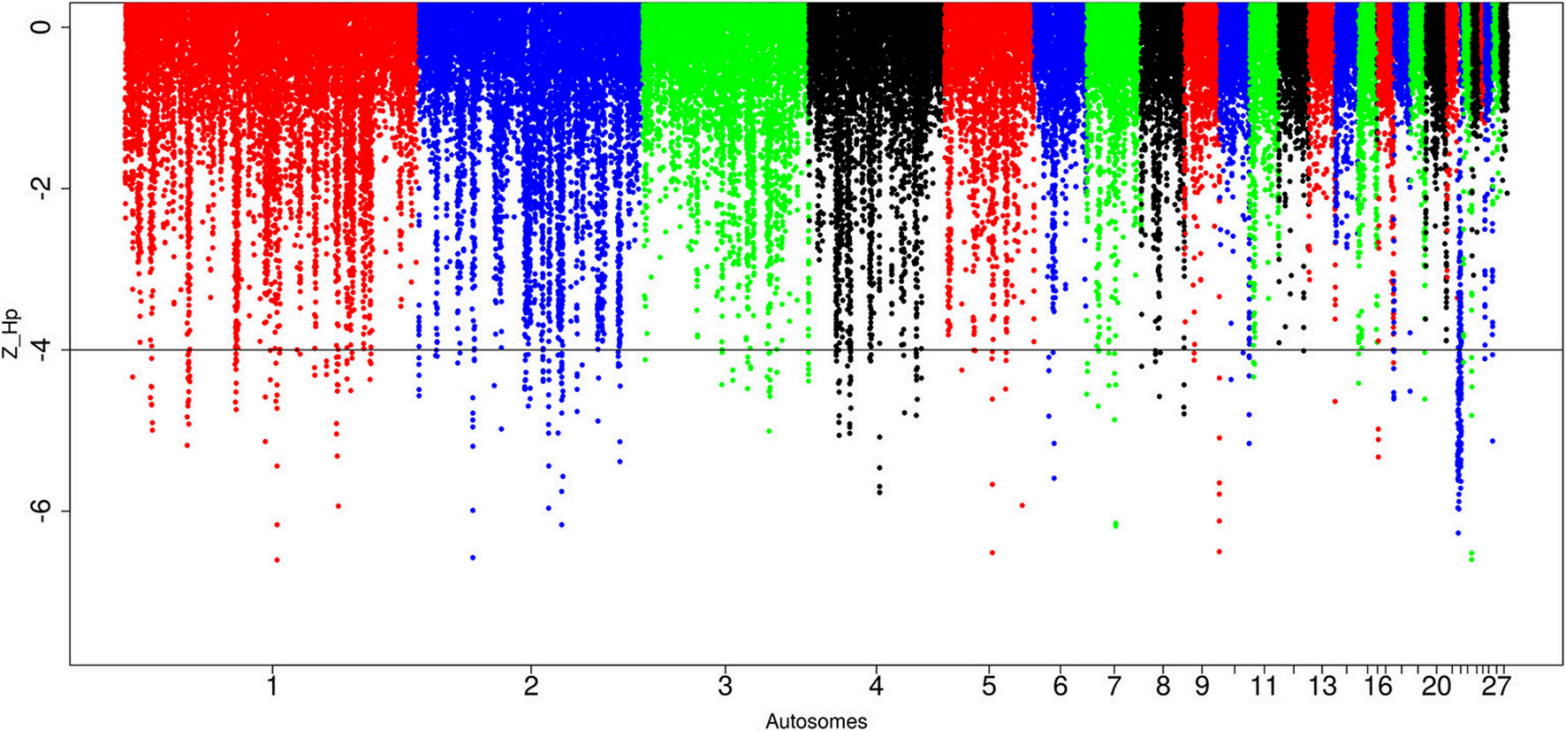
Manhattan plots for selection sweep analysis performed using the standardized pool heterozygosity Z(*H*_p_) approach. The horizontal line represent the arbitrary threshold for Z(*H*_p_) ≤ − 4. This figure shows the selection sweep test for red junglefowl.

A total of 434 out of 90,170 windows passed the genome-wide threshold ≤ −4 resulting in 190 candidates sweep regions (Table 1; Table S4). Genome-wide, a single ~20 kb window located on chromosome 5 (*Galgal* 5.0 position 51895684–51909028 bp) had the lowest Z(*H*_p_) score (−5.93) (Figure 5; Table S4). The region with the largest fragment size (~210 kb, *Galgal* 5.0 position 2376153–2590429 bp, Z(*H*_p_) score = −4.63 ± 0.653) is on chromosome 22. Two other candidate regions >100 kb in size are also present; ~110 kb region on chromosome 2 (*Galgal* 5.0 position 33529–143341 bp) and ~150 kb on chromosome 22 (*Galgal* 5.0 position 578106–728044 bp). Ninety-one candidates sweep regions out of the 190 have fragment sizes of 20 kb, 44 have sizes of 30 kb, 13 have sizes of 40 kb, 17 have sizes of 50 kb, and 25 have sizes of 60 kb and above, respectively. We did not identify any peaks below our threshold on chromosomes 14, 16, 20, 21, 24, 25, 27, and 28 at Z(*H*_p_) score ≤ −4 (Figure 5).

### Selection sweep detection in the domestic chicken

**Figure 6:**
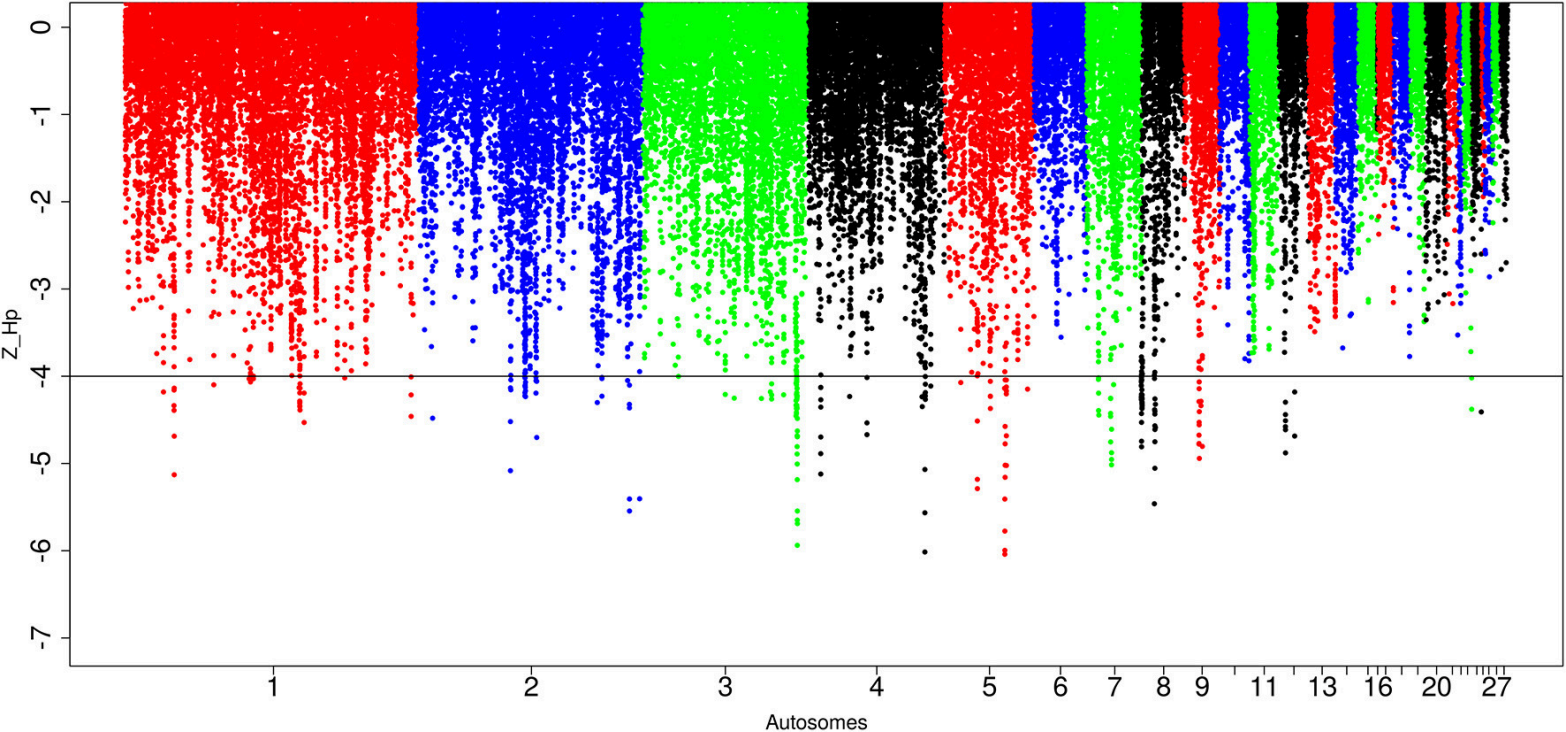
Manhattan plots for selection sweep analysis performed using the standardized pool heterozygosity Z(*H*_p_) approach. The horizontal line represents the arbitrary threshold for Z(*H*_p_) ≤−4. This figure shows the Ethiopian chicken population.

Out of the 89,443 windows analyzed in Ethiopian domestic chicken, 247 windows passed the genome-wide threshold of ≤ −4. They define 84 candidates sweep regions (Table 1; Table S5). The ~50 kb candidate region on chromosome 5 (*Galgal* 5.0 position 40828747–40878736 bp) has the lowest Z(*H*_p_) score (−5.8 ± 0.289) and spans the *TSHR* and *GTF2A1* genes. Genome-wide, the largest candidate sweep region (~210 kb in size, *Galgal* 5.0 position 424781–634785 bp; Z(*H*_p_) score = −4.29 ± 0.055) is on chromosome 8 (Figure 6; Table S5). Three other candidate regions have fragment sizes >100 kb; two on chromosome 3 with a size of ~110 kb (*Galgal* 5.0 position 103157991–103267894 bp) and ~150 kb (*Galgal* 5.0 position 103517529–103667817 bp), respectively, and the other on chromosome 8 (*Galgal* 5.0 position 164536–274537 bp) with a size of ~110 kb (Table S5). The analysis of the fragment sizes of each sweep region found below the genome-wide threshold of Z(*H*_p_) ≤ −4 reveals that 36 candidate regions are 20 kb in size, 13 are 30 kb, nine are 40 kb, ten are 50 kb, and 16 have sizes ≥60 kb. We did not identify any peaks on chromosomes 6, 10, 11, 13, 14, 15, 16, 17, 18, 19, 20, 21, 22, 25 26, 27, and 28 (Figure 6).

**Figure 7:**
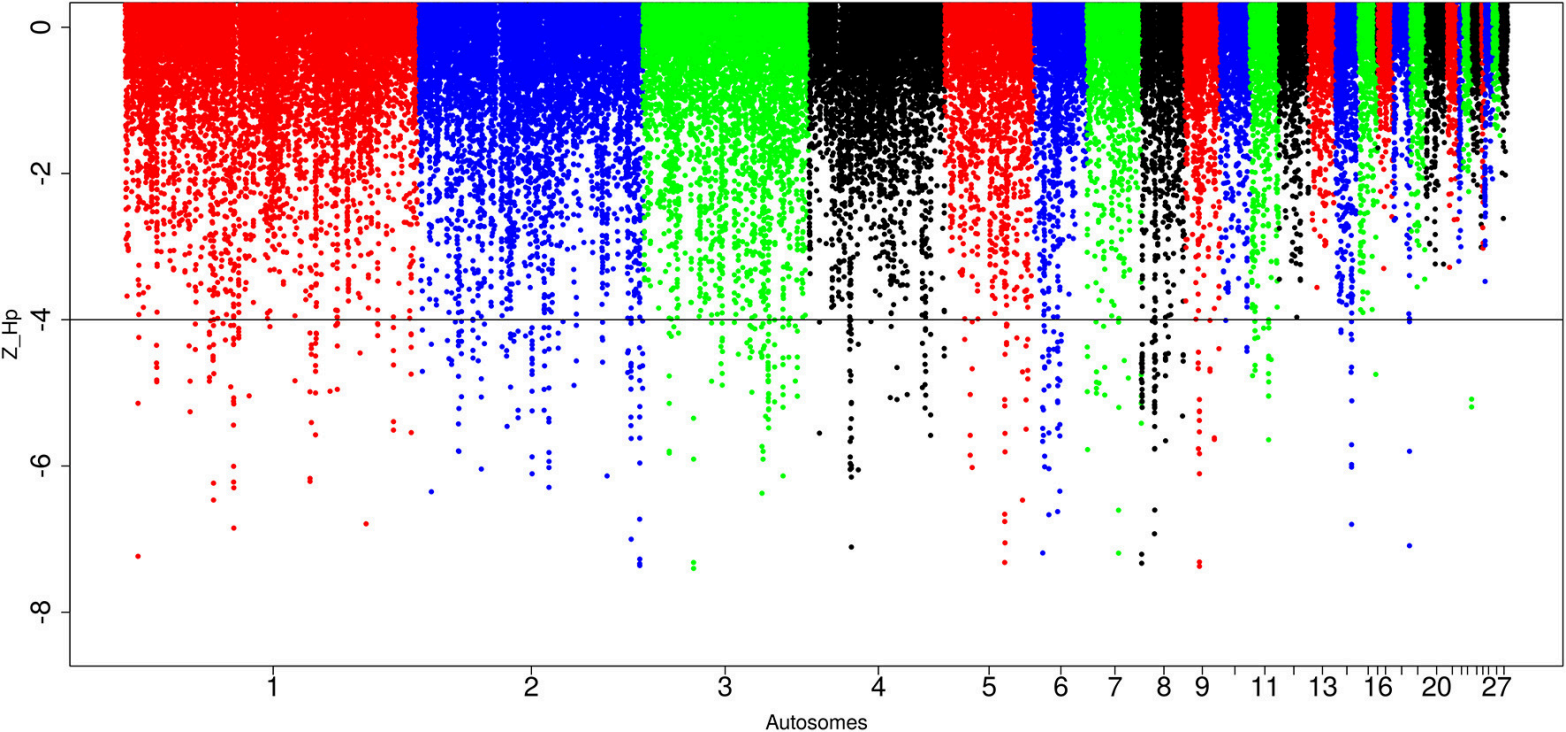
Manhattan plots for selection sweep analysis performed using the standardized pool heterozygosity Z(*H*_p_) approach. The horizontal line represents the arbitrary threshold for Z(*H*_p_) ≤−4. This figure shows the Saudi Arabian chicken population.

For the Saudi Arabian domestic chicken, we identified in total, 87,646 windows out of which 565 passed the genome-wide threshold, defining 212 candidates sweep regions (Table 1; Table S6). The peak with the lowest Z(*H*_p_) score (−7.27 ± 0.087) is ~30 kb region on chromosome 8 (*Galgal* 5.0 position 204536–234537 bp). The largest sweep region (~210 kb in size, *Galgal* 5.0 position 424781–634785 bp; Z(*H*_p_) score = −4.78 ± 0.272) occurs on chromosome 8 at the same position as the largest candidate selected region in Ethiopian chicken (Figure 7; Table S6). Five other candidate selection sweep regions have sizes >100 kb. It includes two regions on chromosome 2 (~140 kb region at *Galgal* 5.0 position 75375947–75512081 bp, and ~110 kb at *Galgal* 5.0 position 147224789–147334917 bp), one region on chromosome 4 (~120 kb in size, *Galgal* 5.0 position 28881313–29001315 bp) and two regions on chromosome 8 (~196 kb length region at *Galgal* 5.0 position 8806310–9002909 bp, and ~113 kb region at *Galgal* 5.0 position 9108796–9221862 bp) (Table S6). Analysing fragment sizes for the selection sweep regions show that 81 out of the 212 candidate regions have a fragment size of 20 kb, 55 have a fragment size of 30 kb, 27 are 40 kb in size, 15 are 50 kb size, and 35 are ≥60 kb in size. We did not identify any peaks below our threshold on chromosomes 12, 13, 16, 17, 19, 20, 21, 22, 24, 25 26, 27, and 28 (Figure 7).

**Figure 8:**
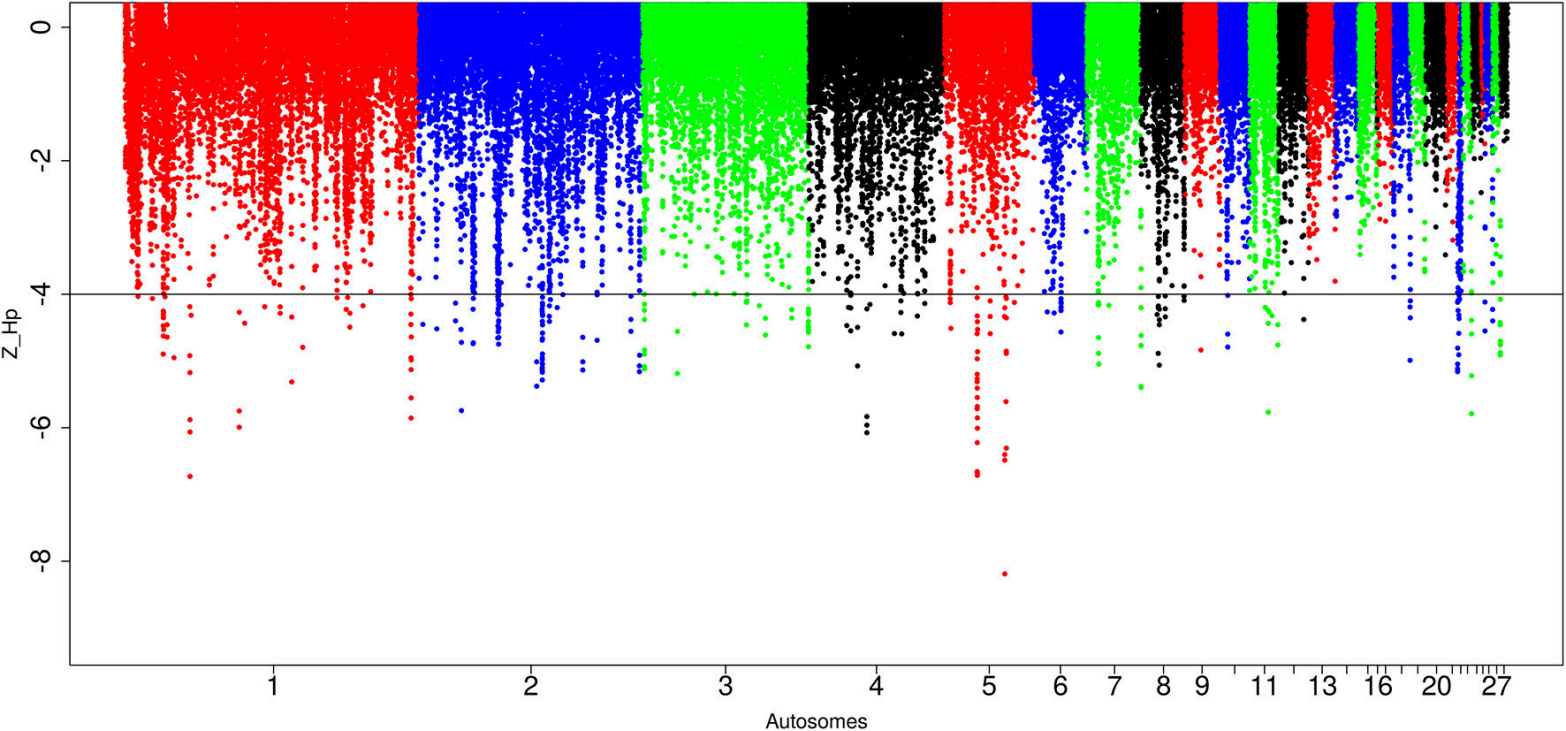
Manhattan plots for selection sweep analysis performed using the standardized pool heterozygosity Z(*H*_p_) approach. The horizontal line represents the arbitrary threshold for Z(*H*_p_) ≤−4. This figure shows the Sri Lankan chicken populations.

In Sri Lankan domestic chicken, of the 89,701 windows detected, 299 passed the genome-wide threshold resulting in 127 candidates sweep regions (Table 1; Table S7). Like Ethiopian chicken, the lowest genome-wide Z(*H*_p_) score (−6.32 ± 1.634) occurs in ~50 kb region on chromosome 5 (*Galgal* 5.0 position 40828747–40878736 bp) (Figure 8; Table S7). The candidate region with the largest fragment size (~290 kb; Z(*H*_p_) score = −4.65 ± 0.454) is located on chromosome 2 (*Galgal* 5.0 position 82190953–82481139 bp). Two other candidate regions have fragment sizes >100 kb. They include a ~130 kb region on chromosome 3 (*Galgal* 5.0 position 111008970–111138863 bp) and a ~220 kb region on chromosome 5 (*Galgal* 5.0 position 22371859–22591888 bp). The analysis of the 127 candidate regions reveal that the length of 63 are 20 kb, 30 are 30 kb, 15 are 40 kb, five are 50 kb, and 14 have sizes ≥60 kb. We did not identify any peak below our threshold on chromosomes 13, 14, 15, 16, 17, 19, 20, 21, 24, 25, and 28 (Figure 8).

### Overlapping sweep regions across populations

**Figure 9:**
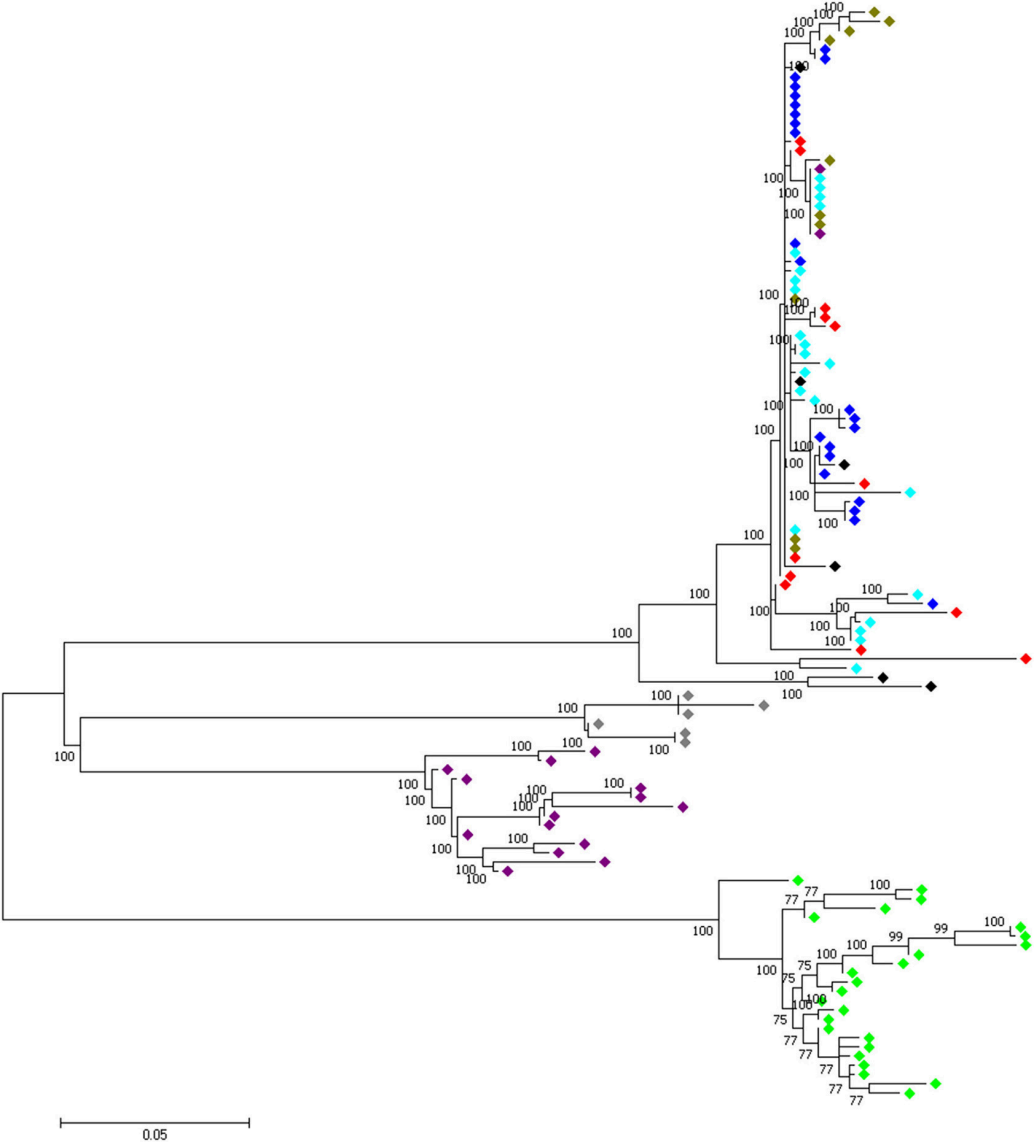
A 30 kb candidate selected region on chromosome 23 (*Galgal* 5.0 position 5521861–5551860) shared between all domestic chicken population and the red junglefowl. 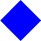, Ethiopian chicken; 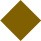, Saudi Arabian chicken; 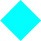, Sri Lankan chicken; 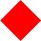, Red junglefowl; 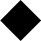, Javan red junglefowl; 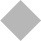, Grey junglefowl; 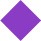, Ceylon junglefowl; 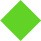, Green junglefowl.

At the genome level, only two sweep regions are common to all domestic chicken and the red junglefowl. They include ~20 kb candidate region on chromosome 7 (*Galgal* 5.0 position 8578942–8598945 bp) within an intergenic region and ~30 kb length on chromosome 23 (*Galgal* 5.0 position 5521861–5551860 bp) spanning three functional genes (*HPCAL4, TRIT1* and *MYCL*) (Table 2). Haplotype trees analysis for the two regions illustrate the variation within the selected haplotypes (Figure 9; Figure S4). One hundred and thirty-two, and 181 variable sites are present across domestic and red junglefowl samples in the 20 and 30 kb regions, respectively (Table 3). It corresponds to an average of 7 and 6 SNPs/kb, well below the combined domestic chicken and red junglefowl populations genome average of 19 SNPs/kb (Figure S2, Table 3).

**Table 2:** Candidate selection sweep regions shared between/among populations.

***Galgal*** **5.0 reference genome coordinates**			**Domestic chicken populations**			**Red junglefowl**	***Galgal* 5.0 reference annotation**
**Chr**	**Start**	**End**	**Ethiopian**	**Saudi Arabian**	**Sri Lankan**	**Red**	**Genes**
1	8655463	8685324		x	x		–
1	25502468	25522471	x		x		–
1	32471039	32491038	x		x		–
1	58796189	58816190	x	x			–
1	82949734	82969734	x	x			–
1	141722332	141782278		x		x	–
1^a^	190947207	190967194	x	x	x		–
2	28171924	28211919		x	x		*5S_rRNA*
2	35402148	35482144		x		x	–
2	60957242	60997244	x	x			–
2	70554878	70585159	x			x	–
2	70821057	70851026	x			x	–
2	71021440	71061441	x	x		x	–
2	78554635	78584572	x		x		–
2	82140679	82160679		x	x		–
2	86485412	86555403		x		x	–
2	86625403	86655403		x		x	–
2	92866622	92886622		x		x	*gga-mir-1803*
2	96092435	96132454		x	x	x	–
2	139258589	139278588	x	x			–
2	141531206	141601206		x	x		*KCNQ3*
2	147144279	147164279		x	x		–
2^a^	147254792	147274793	x	x	x		–
3	53690958	53710958		x		x	–
3	79759035	79779035	x	x			*HMGN3*
3	82484657	82504658		x	x		*RIMS1*
3	84955044	84985013		x		x	–
3	103517529	103667817	x	x			–
3	111068964	111128966			x	x	–
4	27150530	27174313		x	x		–
4	27853766	27873764	x	x			–
4	28621318	28671317		x	x		–
4	39449745	39489745	x		x		*TACR3*
4	42031828	42051827		x		x	–
4	76373927	76391801	x	x			*LCORL*
4	78118133	78168138	x	x			–
4	78407934	78487939	x	x			–
5	22511835	22551887	x		x		–
5^a^	40828747	40878736	x	x	x		*GTF2A1, TSHR*
5^a^	41868268	41908264	x	x	x		–
5	41828256	41848267	x			x	–
5	51895684	51909028		x		x	–
5	55431566	55451566	x	x			*C14orf37*
6	13734753	13764764			x	x	*KCNMA1*
6	18316795	18346799		x	x		–
7	492550	522549		x		x	*COL5A2*
7	8369039	8429044	x		x		–
7^b^	8578942	8598945	x	x	x	x	–
7	15343407	15393407			x	x	–
7	36850252	36880269		x	x		*BAZ2B*
7	36890268	36924725		x	x		*NAA20, Mar-07*
8	164536	274537	x	x			–
8	424781	634785	x	x			–
8	8824776	8844776	x	x			–
8	8894776	8914776	x	x			–
8	9138797	9221862	x	x			–
8	9511843	9541920	x	x			–
8	17163256	17243252		x	x		–
8	29736834	29766834			x	x	*TYW3*
9	10389300	10433014	x	x			*GK5*
9	11499478	11529475	x		x		*PLOD2*
18	10732550	10752550			x	x	*JPT1, SLC16A5, ARMC7*
22	238113	318051			x	x	*ANTXR1, BMP10, ARHGAP25, GKN2*
22	578106	728044			x	x	*PPP2R2A, EBF2*
22	1068084	1098084			x	x	–
22	1098084	1188031			x	x	*STC1*
22	1208020	1258019			x	x	*LOXL2*
22	1536501	1636436			x	x	*DUSP26, MAK16, LZTS1, ATP6V1B2, RNF122, TTI2, SLC18A1*
23^b^	5521861	5551860					*HPCAL4, TRIT1, MYCL*
26	5155940	5205938			x	x	*OPTC, PRELP*

a *Significant regions in the three domestic chicken populations*.

b *Significant regions in domestic chicken population and red junglefowl*.

x *means the candidate region is found selected in the respective population*.

**Table 3:** Number of variable sites (SNPs) within the selected regions.

	**Length (kb)**	**Total number of SNPs in the selected region**	Average SNPs/kb in the selected region^*^
**DOMESTIC CHICKEN SELECTED REGIONS**			
1:190947207–190967194	20	217	11
2:147254792–147274793	20	179	9
5:40828747–40878736	50	207	4
5:41868268–41908264	40	212	5
**DOMESTIC CHICKEN AND RED JUNGLEFOWL SELECTED REGIONS**			
7:8578942–8598945	20	132	7
23:5521861–5551860	30	181	6

* *Average SNPs/kb in the selected region calculated as total number of SNPs in the selected region divided by the length (kb) of the region*.

**Figure 10:**
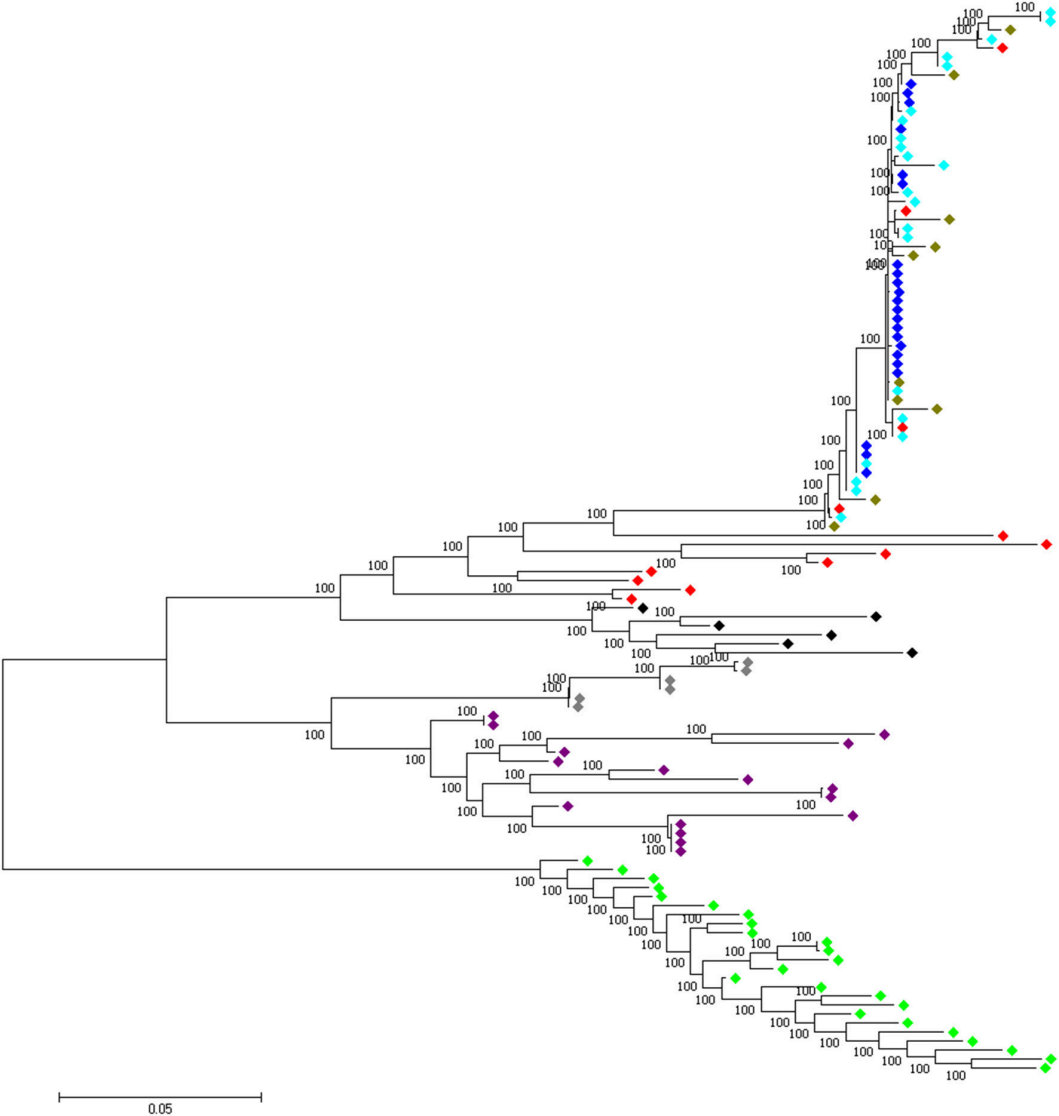
A 50 kb candidate selected region on chromosome 5 (*Galgal* 5.0 position 40828747–40878736) identified in all domestic chicken population. This region includes the *TSHR* and *GTF2A1* loci. 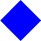, Ethiopian chicken; 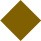, Saudi Arabian chicken; 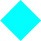, Sri Lankan chicken; 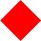, Red junglefowl; 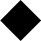, Javan red junglefowl; 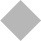, Grey junglefowl; 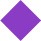, Ceylon junglefowl; 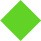, Green junglefowl.

Four candidate selected regions shared between the three domestic chicken populations are identified. One is located on chromosome 1 (~20 kb: *Galgal* 5.0 position 190947207–190967194 bp), one on chromosome 2 (~20 kb: *Galgal* 5.0 position 147254792–147274793 bp) and two on chromosome 5 (~50 kb: *Galgal* 5.0 position 40828747–40878736 bp and ~40 kb: *Galgal* 5.0 position 41868268–41908264 bp) (Table 3). We identified two genes (*TSHR* and *GTF2A1*) within the 50 kb region of chromosome 5, while the 20 kb region on chromosome 2 includes an exon of the transcript ENSGALT00000026040. The two other candidate regions are found within intergenic/intronic regions. Figure 10 and Figures S5–S7 illustrates the haplotype variation. Between 179 and 217 variable sites were identified across these regions or an average of 4 to 11 SNPs/kb (Table 3), lower than the genome average of 16 SNPs/kb calculated for the combined domestic chicken populations genomes (Figure S2).

Among the domestic chicken populations, 18 candidates sweep regions, out of a total of 70, are shared between Ethiopian and Saudi Arabian domestic chicken (Table 2). Four of the regions span annotated genes; *HMGN3* (chromosome 3), *LCORL* (chromosome 4), *C14orf37* (chromosome 5) and *GK5* (chromosome 9). Two out of the six candidate regions that are shared between the Ethiopian and Sri Lankan domestic chickens overlap with genes including *TACR3* (chromosome 4) and *PLOD5* (chromosome 9). The genes present on the 13 candidate sweep regions shared between Saudi Arabian and Sri Lankan domestic chickens include *5S_rRNA* and *KCNQ3* (chromosome 2), *RIMS1* (chromosome 3), *BAZ2B, Mar-07* and *NAA20* (chromosome 7) (Table 2).

### Functional annotations for the enriched genes within the sweep regions

To identify the functions of candidate genes that may have played significant roles in adaptation to production environments and the domestication process, we performed enrichment analysis for all genes identified within the candidate sweep regions. Only classes of genes with default fisher exact *P* < 0.05 were considered overrepresented for the GO and KEGG pathways analysis. The GO results for all populations is found in Table S8 and that of KEGG pathway is found in Table S9.

## Discussion

The autosomal genetic background and adaptation to local production environments of three populations of indigenous domestic village chicken were analyzed alongside the wild progenitor, the red junglefowl, using whole-genome re-sequencing data. Our objectives were to identify candidate positively selected regions (i) shared between wild red junglefowl and domestic chicken, (ii) shared among domestic chicken only and (iii) specific to individual domestic chicken and red junglefowl population.

### Common genome regions selected in both domestic and red junglefowl

Common regions under selection will be expected between a domesticate and its wild ancestor considering their shared evolutionary history. They may correspond, for examples, to species specific signature of selection underlining shared morphological and behavioral phenotypes. It may be particularly true for village indigenous chicken where human selection pressures have been lower compared to commercial and fancy chicken breeds.

We identified two candidates sweep regions that are shared between all domestic chicken and the red junglefowl. While we could not identify any functional genes within the region on chromosome 7, suggesting possibly an important regulatory role for the region, the one on chromosome 23 spanned three candidate genes (*HPCAL4, TRIT1, MYCL*). *HPCAL4* is known to play a role in the development of central nervous system (Kobayashi et al., 1998). However, while the biological functions of *MYCL* is still being studied (Brägelmann et al., 2017), both *TRIT1* and *MYCL* genes have been linked to the maintenance of tumors (Smaldino et al., 2015; Brägelmann et al., 2017). All three genes may be of importance in both domestic and the wild ancestor; *HPCAL4* in relation to behavioral characteristics, *TRIT1* and *MYCL* in relation to adaptation to retrovirus infection in particular to virus causing tumors (e.g., leukosis and Marek virus) commonly affecting chicken (Cheng et al., 2010; Wragg et al., 2015).

### Domestic chicken specific signature of selection

Candidate signature of positive selection specific to domestic chicken may originate from the domestication process itself or after the domestication of the species following geographic dispersion and local responses to human and natural selection pressures. The distinction between the two is difficult. It may be approached using ancient DNA studies (Flink et al., 2014; Loog et al., 2017). We can also expect that genome regions selected at an early stage of the domestication process, prior to the geographic dispersion of the domesticate will be present in most if not all populations. Compared to fancy chicken breeds and commercial chicken lines, that are characterized by smaller effective population sizes and are heavily selected by humans, the indigenous domestic village chicken, with large effective population sizes, uncontrolled mating and relaxed artificial selection, may represent a better model for the identification of such regions.

We identified four candidate genome regions under positive selection in all the domestic chicken populations but not in the red junglefowl (see Table 2). Excluding one region on chromosome 2, these regions have all been previously identified in commercial broilers and layers (Rubin et al., 2010) adding support to early selected domestic region. For the region on chromosome 2, Johnsson et al. (2016) also reported a selected candidate region on this chromosome (*Galgal* 5.0 position 147194251–147234789 bp) which falls 20-kb away from ours (*Galgal* 5.0 position 147254792–147274793 bp). This region is only found in domestic chicken and not in feral birds, and it may be therefore of relevance to the domestication process.

For the remaining three regions, the 50 kb selected region on chromosome 5 includes two genes; the *TSHR* locus involved in metabolic regulation and reproduction process (Yoshimura et al., 2003; Hanon et al., 2008; Rubin et al., 2010) and *GTF2A1*, a candidate biomarker for detecting ovarian tumor (Huang et al., 2009). Hanon et al. (2008) reports that *TSH*-expressing cells of the pars tuberalis is linked to seasonal reproductive control in vertebrates and therefore to the onset of egg laying (Loog et al., 2017). We now know from the studies of Flink et al. (2014) and Loog et al. (2017) that selection at the *TSHR* in European chicken likely followed the selection for higher egg production characteristics. Our studies indicate that similar selection pressures may have acted on Ethiopian, Saudi Arabian, and Sri Lankan domestic chicken. Analysis of chicken populations from different parts of the world, e.g., East and South Asia is required.

### Signatures of selection in relation to the production environments

Response to selection is environmentally driven either naturally or artificially (Oleksyk et al., 2010). The ancestral species of domestic chicken, the red junglefowl, has a very large geographic range (Delacour, 1977). While different wild red junglefowl subspecies and domestic chicken populations may be witnessing different environmental challenges (e.g., altitudes), all are living in regions that are characterized by rather a warm climate and substantial rainfall which however may show considerable annual variation (e.g., monsoon cycles) or daily variation (e.g., temperature difference during the day). Accordingly, signatures of selection related to thermotolerance including temperature and humidity may be expected in domestic chicken and the red junglefowl.

In Ethiopian chicken, we identified two candidate genes, *HRH1* and *AGTR1*, associated with “vasoconstriction regulation.” Vasoconstriction has been linked to reduction in peripheral blood flow leading to increase in internal body temperature (Sessler et al., 1990). These genes may likely play important roles in thermoregulation (Collier and Collier, 2011; Su et al., 2011). The reduction in evaporative heat loss and stress through decreased cutaneous blood flow has been reported previously in cattle and birds (Collier and Collier, 2011; Klotz et al., 2016). Compared to the average chicken body temperature of 41°C (Bolzani et al., 1979), the ambient temperatures of Horro and Jarso districts are relatively low (19 to 21°C) and the two selected candidate genes may played important roles in adaptation to their local environments. At the opposite, Saudi Arabia is very dry with extreme heat during the day which could rise above 50°C in July/August. Here, we identified several GO terms such as “blood circulation,” “regulation of heart contraction,” “regulation of muscle system process,” “regulation of muscle adaptation,” and “regulation of cardiac muscle contraction” that may be linked to the control of blood flow and evaporative cooling (Collier and Collier, 2011). Other studies have associated some of these GO terms to oxygen deprivation response due to high altitude adaptation (Li et al., 2013; Wang et al., 2015). However, this causative explanation is unlikely in our case because the Saudi Arabian chicken were sampled at an altitude of about 100 m asl. Considering the climatic conditions of the sampling area, we favor here the link to heat loss in response to extreme heat. The significantly enriched GO terms, cellular response to hydrogen peroxide and toll-like receptor signaling pathways, observed in Saudi Arabian chickens may suggest strong selection as well in response to disease challenges (Medzhitov, 2001; Stone and Yang, 2006).

In the genomes of Saudi Arabian and Sri Lankan domestic chicken alongside the red junglefowl, we uncovered the *KCNMA1* gene, that may be linked to hypoxia response challenge. The region harboring this gene did not come as significant in the Ethiopian chicken. *KCNMA1* is associated with the regulation of smooth muscle contraction through the activation of calcium ions (Williams et al., 2004). Increase in calcium ions stimulates hypoxia-inducible factor-1 (Hui et al., 2006). However, the biological roles played by this gene in red junglefowl and Saudi Arabian or Sri Lankan domestic chicken may be different. While in the two domestic chicken populations, it may be related, to heat tolerance and stress control considering the low elevations of the sampling sites; in the red junglefowl however, it may rather play a role in adaptation to high altitudes. Both the Yunnan (altitude ~3,000 m asl) and Hainan (altitude ~1,840 m asl) provinces, where the two red junglefowls were sampled, are mountainous. High elevations are associated with decrease in arterial oxygen content (Simonson et al., 2010). Another gene, *ADAM9*, detected in our red junglefowl, which plays a role in the development of cardiorespiratory system has also been proposed to be involved in adaptation to high-altitude in Tibetan chicken (Zhang et al., 2016).

*KCNMA1* and *ADAM9* were not detected in the candidate regions in Ethiopian chicken. These chickens live at an altitude of around 2,000 m asl. Perhaps, neither the climate and/or altitude where Horro and Jarso populations live result in strong selection pressures in their genomes. Analysis of Ethiopian chicken, living at much higher altitudes may provide further insights on the possible roles of *KCNMA1* and *ADAM9* in altitude adaptation in African domestic chicken.

In addition, one of the previously reported gene under selection in commercial chicken (Rubin et al., 2010; Johnsson et al., 2016), *NT5C1A*, was also identified in the red junglefowl and Sri Lankan indigenous domestic chicken studied here. Importantly, this gene is known to be involved in regulating the levels of heart adenosine during hypoxia and ischemia especially when blood supply becomes inadequate in some parts of the body (Hunsucker et al., 2001). The detection of hypoxia adaptation in both the red junglefowl and domestic chicken may or may not be related to environmental conditions. However, it is well documented that activities relating to extreme exercise may induce hypoxia (Springer et al., 1991; Lindholm and Rundqvist, 2016). Wild and domestic cocks are most often aggressive in nature with the latter having a long history of being selected for cock fighting (Delacour, 1977). We could then argue that the aggressiveness already presents in the wild relative, due in part to predator evasion and sexual selection behaviors, which can be seen as extreme exercise, may have undergone positive selection in most domestic chicken populations.

## Conclusions

Examining signature of selection in both domestic chicken and red junglefowl, our study reveals that only two candidate positive selected regions are common to both while four regions are shared across the domestic populations only. Proviso of the relatively low number of red junglefowl examined and the lack of consensus on the geographic origin of the domestic centers of the species, our results illustrate the major impact of human selection activities on the species, and the consequences on the genome landscape of adaptations to new environments. It exemplifies how quickly a domestic species may evolve when under selection pressures in environments.

## Author contributions

RL and OH conceived and designed the project. PS contributed the DNA and provided knowledge on the Sri Lankan chicken. RA-A, RA, and JM provided the Saudi Arabian chicken samples and their genome sequences. RA-A provided knowledge on the Saudi Arabian chicken and the sampling area. RL performed the analyses and OH supervised the project and contributed substantial knowledge on the interpretation of the results. RL prepared and wrote the manuscript. JM and OH revised the manuscript. All the authors read and approved the final manuscript.

### Conflict of interest statement

The authors declare that the research was conducted in the absence of any commercial or financial relationships that could be construed as a potential conflict of interest.

## Footnotes

^1^http://www.ccrp.be/

^2^http://picard.sourceforge.net

^3^https://www.cog-genomics.org/plink2

^4^http://www.fluxus-engineering.com/sharenet.htm

^5^https://david.ncifcrf.gov/

^6^http://kobas.cbi.pku.edu.cn/
