# Supplementary material for: Whole-Genome Resequencing of Red Junglefowl and Indigenous Village Chicken Reveal New Insights on the Genome Dynamics of the Species
Source: Front Genet. 2018 Jul 20;9:264. doi: 10.3389/fgene.2018.00264 (PMC6062655; doi:10.3389/fgene.2018.00264)
Supplement: Supplementary file 1 [file Table_1.PDF]

Table S1 | Summary of sequencing statistics and numbers of SNPs detected

**Sample**	**Reads**	**PPR (%)**	**MR (%)**	**MD (X)**	**5_b (%)**	**10_b (%)**	**20_b (%)**	**Hom**	**Het**	**Total SNPs**
HA2A25B[a]	404491652	93.75	99.27	32.38	97.9	96.7	91.3	2663926	3625010	6288936
HA1B25B[a]	375265404	93.68	98.77	31.05	98.1	96.8	89.7	2731530	3490701	6222231
HB1A16A[a]	402727784	93.55	98.79	31.93	98.2	97	90.8	2663242	3678469	6341711
HB1B21B[a]	406780167	93.92	98.87	32.51	98.1	96.9	91.1	2683493	3611342	6294835
HA1A22A[a]	386714341	94.48	98.84	31.52	98.1	96.9	90.4	2978357	3010187	5988544
HA2A10B[a]	408859746	92.63	98.84	32.81	98.2	97.1	91.4	2773136	3440448	6213584
JA2A10B[a]	399745882	93.38	98.79	32.04	98.1	96.9	90.7	2865428	3210674	6076102
JB1B16A[a]	363393410	94.61	99.36	29.31	98.4	96.2	83.1	2658765	3617612	6276377
JB1A25B[a]	382829596	93.76	98.88	32.04	98.1	96.9	90.7	3081092	2741341	5822433
JB2A04B[a]	401918477	93.25	98.31	31.85	98.1	96.8	90.3	3185080	2589279	5774359
JA1A17A[a]	396334439	91.52	97.61	31.55	98	96.7	89.5	2822391	3291155	6113546
Saudi Arabia1[b]	108937623	91.83	98.87	13.75	96.1	78.2	8.7	3011627	2475976	5487603
Saudi Arabia2[b]	109127614	91.97	98.64	13.79	95.7	75.5	10	2563278	3487603	6050881
Saudi Arabia3[b]	108810954	92.71	98.02	10.22	87.4	43.2	4.4	2626554	3051262	5677816
Saudi Arabia4[b]	109038659	94.2	98.73	13.87	96.3	78.8	9	1768821	4878947	6647768
Saudi Arabia5[b]	109394273	92.41	98.53	10.5	91.5	50.9	2.1	2523799	3350890	5874689
Sri Lanka1[c]	272007288	92.75	98.74	31.46	97.9	96.4	87.7	2427647	4167743	6595390
Sri Lanka2[c]	269162107	92.84	98.65	31.03	98	96.4	87.7	2380427	4236500	6616927
Sri Lanka3[c]	262738537	92.9	98.77	30.45	98	96.3	86.4	2428299	4166738	6595037
Sri Lanka4[c]	269326187	93.02	98.57	31.05	98	96.4	86.9	2365620	4349780	6715400
Sri Lanka5[c]	281834982	92.6	98.82	32.41	98.5	96.5	85.3	2395630	4285906	6681536
Sri Lanka9[c]	267558558	92.73	98.78	30.9	98.4	96.1	83	2333190	4347940	6681130
Sri Lanka10[c]	276733672	92.68	98.65	31.89	98.5	96.4	84.2	2462458	4110133	6572591
Sri Lanka12[c]	269404630	92.48	98.8	31.23	98	96.5	87.2	2365932	4326208	6692140
Sri Lanka13[c]	249093732	91.34	98.72	28.73	98.3	95.4	79.3	2461960	4099930	6561890
Sri Lanka15[c]	229370558	92.89	98.99	26.8	97.3	93.6	74.7	2453733	4150327	6604060
Sri Lanka16[c]	251649616	93.18	98.8	29.2	97.9	96.1	84.5	2428773	4249479	6678252
Red junglefowl1[d]	154287870	93.85	97.72	12.96	93.4	70.2	9.8	2524724	3573351	6098075
Red junglefowl2[d]	286656521	95.33	98.93	25.6	98.7	95.8	76.2	2561487	4115394	6676881

Red junglefowl3[d]	186743667	94.21	99.14	15.9	95.1	77.1	27.7	2633783	4364117	6997900
Red junglefowl4[d]	224231071	95.5	99.07	19.09	97.1	92.8	43.2	2974376	3800042	6774418
Red junglefowl5[d]	403120509	94.8	98.91	36.05	98.2	97.3	93.4	2540845	4919100	7459945
Red junglefowl_koen[e]	149692501	90.08	94.69	15.97	96.6	83.5	20.8	2393695	4754034	7147729

[a] Population from Ethiopia including Horro samples (name start with H) and Jarso samples (name start with J)

[b] Population from Saudi Arabia

[c] Population from Sri Lanka

[d] Red junglefowl sequences retrieved from "Wang, M.-S., Li, Y., Peng, M.-S., Zhong, L., Wang, Z.-J., Li, Q.-Y., et al. (2015). Genomic analyses reveal potential independent adaptation to high altitude in Tibetan chickens. *Mol. Biol. Evol.* **32**, 1880-1889"

[e] Red junglefowl sequence from Cosmopolitan Chicken Research Project (http://www.ccrp.be/#/0).

PPR: Percentage of both mates of a read pair that were properly mapped to the same chromosome.
MR: Total reads mapped to the reference genome.
MD: The mean sequence depth or the genome sequence coverage.
5_b: Percentage of the genome with bases covered by at least 5 reads.
10_b: Percentage of the genome with bases covered by at least 10 reads.
20_b: Percentage of the genome with bases covered by at least 20 reads.
Hom: Homozygous SNPs compare to the reference.
Het: Heterozygous SNPs compare to the reference.
The **Total SNPs** includes the bi-allelic **Hom** + **Het.**
