# Supplementary material for: Whole-Genome Resequencing of Red Junglefowl and Indigenous Village Chicken Reveal New Insights on the Genome Dynamics of the Species
Source: Front Genet. 2018 Jul 20;9:264. doi: 10.3389/fgene.2018.00264 (PMC6062655; doi:10.3389/fgene.2018.00264)
Supplement: Supplementary file 2 [file Table_2.PDF]

Table S2 | Reference haplogroup sequences for the D-loop mitochondrial DNA analysis. The nomenclature of the haplogroups can be found in Mwacharo *et al.,* (2011)

Haplogroup	D-loop sequence
A	AATTTTATTTTTTAACCTAACTCCCCTACTAAGTGTACCCCCCCTTTCCCCCCCAGGGGGGGTATACTATGCATAATCGTGCATACATTTATATACCAC<br>ATATATTATGGTACCGGTAATATATACTATATATGTACTAAACCCATTATATGTATACGGGCATTAATCTATATTCCACATTTCTCCCAATGTCCATTCT<br>ATGCATGATCCAGGACATACTCATTCACCCTCCCCATAGACAGCTCCAAACCACTACCAAGTCACCTAACTATGAATGGTTGCAGGACATAAATCTCA<br>CTCTCATGCTCTTCCCCCAACAAGTCACCTAACTATGAATGGTTACAGGACATACATTTAACTACCATGTTCTAACCCATTTGGTTATGCTCGCCGTATC
B	AATTTTATTTTTTAACCTAACTCCCCTACTAAGTGTACCCCCCCTTTCCCCCCCAGGGGGGGTATACTATGCATAATCGTGCATACATTTATATACCACAT<br>ATATTATGGTACCGGTAATATATACTATATATGTACTAAACCCATTATATGTATACGGGCATTAACCTATATTCCACATTTCTCCCAATGTCCATTCTATGC<br>ATGATCCAGGACATACTCATTTACCCTCCCCATAGACAGTTCCAAACCACTATCAAGCCACCTAACTATGAATGGTTACAGGACATAAATCTCACTCTCA<br>TGTTCTCCCCCCAACAAGTCACCTAACTATGAATGGTTACAGGACATACATTTAACTACCATGTTCTAACCCATTTGGTTATGCTCGCCGTATC
C	AATTTTATTTTTTAACCTAACTCCCCTACTAAGTGTACCCCCCCTTTCCCCCCCAGGGGGGGTATACTATGCATAATCGTGCATACATTTATATACCACAT<br>ATATTATGGTACCGGTAATATATACTATATATGTACTAAACCCATTATATGTATACGGGCATTAATCTATATTCCACATTTCTCCCAATGTCCATTCTATGC<br>ATGATCCAAGACATACTCATTCACCCTCCCCATAGACAGTTCTAAACCACTATCAAGCCACCTAACTATGAATGGTTACAGGACATAAATCTCACTCTCA<br>TGTTCTCCCCCTAACAAGTCACCTAACTATGAATGGTTACAGGACATACATTTAACTACCATGTTCTAACCCATTTGGTTATGCTCGCCGTATC
D	AATTTTATTTTTTAACCTAACTCCCCTACTAAGTGTACCCCCCCTTTCCCCCCCAGGGGGGGTATACTATGCATAATCGTGCATACATTTATATACCACAT<br>ATATTATGGTACCGGTAATATATACTATATATGTACTAAACCCATTATATGTATACGGGCATTAATCTATATTCCACATTTCTCCCAATGTCCATTCTATGC<br>ATGATCCAGGACACACTCATTCACCCTCCCCATAGACAGCTCCAAACCACTACCAAGTCACCTAACTATGAATGGTTACAGGACATAAATCTCACTCTCA<br>TGTTCTTCCCCCAACAAGTCACCTAACTATGAATGGTTACAGGACATACATTTAACTACCATGTTCTAACCCATTTGGTTATGCTCGCCGTATC
E	AATTTTATTTTTTAACCTAACTCCCCTACTAAGTGTACCCCCCCTTTCCCCCCCAGGGGGGGTATACTATGCATAATCGTGCATACATTTATATACCACAT<br>ATATTATGGTACCGGTAATATATACTATATATGTACTAAACCCATTATATGTATACGGGCATTAATCTATATTCCACATTTCTCCCAATGTCCATTCTATGC<br>ATGATCCAGGACATACTCATTCACCCTCCCTACAGACAGCTCCAAACCACCACCAAGTCACCTAACTATGAATGGTTACAGGACATAAATCTCACTCTCA<br>TGTTCTTCCCCTACCAAGTCACCTAACTATGAATGGTTACAGGACATACATTTAACTACCATGTTCTAACCCATTTGGTTATGCTCGCCGTATC
F	AATTTTATTTTTTAACCTAACTCCCCTACTAAGTGTACCCCCCCTTTCCCCCCCAGGGGGGGTATACTATGCATAATCGTGCATACATTTATATACCACA<br>TATATTATGGTACCGGTAATATATACTATATATGTACTAAACCCATTATATGTATACGGGCATTAATCTATATTCCACATTTCTCCCAATGTCCATTCTATG<br>CATGATCCAAGACATACTCATTCACCCTCCCCATAGACAGCTCCAAACCACTACCAAGTCACCTAACTATGAATGGTTACAGGACATAAATCTAACTCTT<br>ATGTTCTTCCCCTAACAAGCCACCTAACTATGAATGGTTACAGGACATACATTTAACTACCATGTTCTAACCCATTTGGTTATGCTCGCCGTATC

Mwacharo, J., Bjørnstad, G., Mobegi, V., Nomura, K., Hanada, H., Amano, T., et al. (2011). Mitochondrial DNA reveals multiple introductions of domestic chicken in East Africa. *Mol. Phylogenet. Evol. 58*(2), 374-382.
