# Supplementary material for: Whole-Genome Resequencing of Red Junglefowl and Indigenous Village Chicken Reveal New Insights on the Genome Dynamics of the Species
Source: Front Genet. 2018 Jul 20;9:264. doi: 10.3389/fgene.2018.00264 (PMC6062655; doi:10.3389/fgene.2018.00264)
Supplement: Supplementary file 3 [file Table_3.PDF]

Table S3 | Variants SNPs statistics and annotations at population level

**Variant consequences**	**Ethiopian Domestic**	**Saudi Arabian Domestic**	**Sri Lankan Domestic**	**Red Junglefowl**
Total number of sample	11	5	11	6
Total number of bi-allelic SNPs	13,075,004	10,231,837	14,455,255	15,317,274
Novel Variants	1,765,053	1,029,993	2,325,291	4,451,313
Existing Variants	11,309,951	9,201,844	12,129,964	10,865,961
Splice donor variant	643	444	693	730
Splice acceptor variant	570	496	603	653
Stop gained	1154	886	1302	1281
Stop lost	307	265	345	371
Start lost	365	303	416	428
Missense variant	74,488	57,637	83,438	87,272
Splice region variant	26,015	20,522	28,811	30,350
Stop retained variant	79	59	80	92
Synonymous variant	123,041	98,486	138,077	145,705
Coding sequence variant	3	1	4	1
Mature miRNA variant	170	133	197	214
5 prime UTR variant	51,655	40,869	57,787	60,366
3 prime UTR variant	246,270	193,040	273,686	289,436
Non-coding transcript exon variant	133,818	10,6657	148,262	158,914
Intron variant	7,058,272	554,4587	7,791,825	8,244,547
Upstream gene variant	758,660	604,830	838,568	885,235
Downstream gene variant	568,176	451,103	626,089	664,720
Intergenic variant	4,031,318	3,111,519	4,465,072	4,746,959
