# Supplementary material for: Whole-Genome Resequencing of Red Junglefowl and Indigenous Village Chicken Reveal New Insights on the Genome Dynamics of the Species
Source: Front Genet. 2018 Jul 20;9:264. doi: 10.3389/fgene.2018.00264 (PMC6062655; doi:10.3389/fgene.2018.00264)
Supplement: Supplementary file 4 [file Table_4.PDF]

Table S4 | Candidate selective sweep regions detected in red junglefowl population using H_p. The **Ggal** is the reference genome annotation for the *Galgal* version 4 or 5, **Ln** is length in kilobase, **nWd** is the number of windows analysed for the sweep region.

**Chr**	**Ggal4_start**	**Ggal4_stop**	**Ln**	**nWd**	**Total SNP**	**mean (H_p)**	**mean Z(H_p)**	**Ggal4_gene**	**Ggal5_start**	**Ggal5_stop**	**Ggal5_gene**
1	4950000	4970000	20	1	168	0.19	-4.34	-	5069405	5104866	-
1	16750000	16770000	20	1	181	0.19	-4.59	-	16952247	16972255	-
1	17290000	17310000	20	1	59	0.19	-4.45	*FAM19A5*	17491327	17511336	*FAM19A5*
1	17560000	17610000	50	4	147	0.19 ± 0.008	-4.48 ± 0.27	-	17761917	17812482	-
1	18060000	18090000	30	2	156	0.18 ± 0.002	-4.95 ± 0.065	-	18261775	18291774	-
1	41210000	41240000	30	2	286	0.18 ± 0.007	-5.01 ± 0.25	-	41317078	41347077	-
1	41720000	41790000	70	6	104	0.21 ± 0.036	-3.64 ± 1.257	*LRRIQ1, ALX1*	41826212	41896214	*ALX1, LRRIQ1*
1	41880000	41910000	30	2	80	0.2 ± 0.002	-4.21 ± 0.062	-	41986136	42016136	-
1	42210000	42300000	90	8	151	0.19 ± 0.007	-4.43 ± 0.255	*MGAT4C*	42327465	42417466	-
1	42380000	42410000	30	2	151	0.2 ± 0.004	-4.12 ± 0.142	-	42497466	42527478	-
1	42980000	43010000	30	2	111	0.19 ± 0.001	-4.36 ± 0.05	*FAR2*	43097039	43127040	-
1	73000000	73020000	20	1	194	0.2	-4.18	*FAR2*	72938488	72958490	-
1	73320000	73340000	20	1	242	0.19	-4.65	*CCND2*	73258531	73278336	*CCND2*
1	73630000	73700000	70	6	83	0.2 ± 0.006	-4.14 ± 0.228	*KCNA1*	73605981	73674409	*KCNA1*
1	73990000	74010000	20	1	68	0.2	-4.14	*NTF3*	73966929	73986959	*NTF3*
1	93150000	93180000	30	2	174	0.18 ± 0.011	-4.86 ± 0.39	-	93794617	93824618	-
1	96500000	96530000	30	2	154	0.2 ± 0.007	-4.19 ± 0.258	-	97155922	97185922	-
1	100180000	100210000	30	2	256	0.19 ± 0.009	-0.44 ± 0.333	*NCAM2*	100870906	100900908	*NCAM2*
1	100940000	101010000	70	6	264	0.17 ± 0.026	-5.32 ± 0.905	-	101604382	101671296	-
1	102620000	102640000	20	1	136	0.2	-4.04	-	103284233	103304233	-
1	116290000	116310000	20	1	126	0.2	-4.05	-	116803463	116823463	-
1	125890000	125910000	20	1	142	0.2	-4.22	*PUDP*	126535044	126555056	*PUDP*
1	126080000	126100000	20	1	140	0.19	-4.31	-	126724651	126744650	-
1	133830000	133870000	40	3	151	0.2 ± 0.002	-4.22 ± 0.085	-	134546355	134586833	-
1	140470000	140530000	60	5	145	0.19 ± 0.014	-4.45 ± 0.496	-	141190145	141250147	-
1	140990000	141050000	60	5	163	0.19 ± 0.012	-4.59 ± 0.43	-	141722332	141782278	-

1	141550000	141600000	50	4	174	0.2 ± 0.005	-4.17 ± 0.19	-	142297793	142347867	-
1	141730000	141760000	30	2	240	0.18 ± 0.037	-5.01 ± 1.307	-	142478079	142508079	-
1	147430000	147450000	20	1	150	0.2	-4.17	-	148200414	148220430	-
1	147500000	147530000	30	2	108	0.2 ± 0.005	-4.25 ± 0.161	-	148270430	148300616	-
1	149980000	150000000	20	1	117	0.19	-4.51	-	150769621	150789620	-
1	150800000	150830000	30	2	230	0.2 ± 0.001	-4.07 ± 0.047	-	151586377	151616202	-
1	162740000	162770000	30	2	110	0.2 ± 0.005	-4.25 ± 0.16	*PCDH17*	163427031	163457027	*PCDH17*
1	163380000	163410000	30	2	208	0.2 ± 0.002	-4.11 ± 0.086	-	164066156	164096161	-
2	30000	140000	110	10	65	0.2 ± 0.01	-4.03 ± 0.358	*SMARCD3, CHPF2, ABCF2*	33529	143341	*ABCF2, SMARCD3*
2	36860000	36890000	30	2	154	0.2 ± 0	-4.12 ± 0.012	*ZNF385D*	36418635	36448634	*ZNF385D*
2	11110000	11130000	20	1	154	0.2	-4.07	-	11172889	11192886	-
2	11630000	11650000	20	1	214	0.2	-4.01	-	11693648	11713649	-
2	12120000	12140000	20	1	189	0.2	-4.08	-	12190839	12210910	-
2	25810000	25830000	20	1	126	0.2	-4.16	-	25735001	25754883	-
2	27290000	27310000	20	1	139	0.2	-4.04	-	27211890	27231890	-
2	35840000	35920000	80	7	109	0.17 ± 0.021	-5.28 ± 0.728	-	35402148	35482144	-
2	36750000	36770000	20	1	201	0.2	-4	-	36308665	36328643	-
2	50110000	50130000	20	1	139	0.19	-4.44	*POU6F2*	49686779	49706779	*POU6F2*
2	54830000	54850000	20	1	210	0.18	-4.98	*ADCY1*	54917813	54937809	*ADCY1*
2	70370000	70400000	30	2	148	0.19 ± 0.001	-4.47 ± 0.02	-	70554878	70585159	-
2	70640000	70670000	30	2	154	0.19 ± 0.002	-4.43 ± 0.075	-	70821057	70851026	-
2	70750000	70770000	20	1	132	0.2	-4.13	-	70931388	70951385	-
2	70850000	70870000	20	1	126	0.19	-4.37	-	71031438	71051451	-
2	71030000	71050000	20	1	179	0.2	-4.06	-	71211288	71231327	-
2	71180000	71200000	20	1	192	0.2	-4.28	-	71361195	71381205	-
2	72710000	72740000	30	2	163	0.19 ± 0.013	-4.38 ± 0.453	-	72909251	72939223	-
2	73980000	74010000	30	2	203	0.19 ± 0.003	-4.54 ± 0.091	-	74191145	74221140	-
2	82420000	82440000	20	1	131	0.2	-4.08	-	82661398	82681343	-
2	82470000	82490000	20	1	117	0.2	-4.12	-	82711342	82731341	-

2	82510000	82530000	20	1	151	0.2	-4	-	82751341	82771342	-
2	82600000	82650000	50	4	187	0.19 ± 0.002	-4.32 ± 0.065	-	82841405	82891406	-
2	86210000	86280000	70	6	177	0.18 ± 0.029	-4.68 ± 1.012	-	86485412	86555403	-
2	86350000	86380000	30	2	178	0.18 ± 0.023	-4.85 ± 0.83	-	86625403	86655403	-
2	86470000	86500000	30	2	119	0.2 ± 0.005	-4.25 ± 0.166	*5S_rRNA*	86745402	86775729	*5S_rRNA*
2	92490000	92510000	20	1	207	0.2	-4.07	*NETO1*	92806623	92826623	-
2	92550000	92570000	20	1	203	0.17	-5.03	*MIR1803*	92866622	92886622	*gga-mir-1803*
2	93440000	93470000	30	2	156	0.2 ± 0.004	-4.16 ± 0.146	-	93753805	93783785	-
2	93900000	93920000	20	1	117	0.2	-4.27	*DOK6*	94213760	94233769	*DOK6*
2	94880000	94910000	30	2	203	0.19 ± 0	-4.41 ± 0.007	-	95194384	95224385	-
2	94940000	95010000	70	6	169	0.18 ± 0.027	-4.85 ± 0.957	-	95254294	95324294	-
2	95010000	95060000	50	4	153	0.2 ± 0.004	-4.16 ± 0.136	-	95324294	95374294	-
2	95090000	95120000	30	2	122	0.2 ± 0.001	-4.21 ± 0.047	-	95404293	95434290	-
2	95750000	95790000	40	3	149	0.18 ± 0.017	-4.89 ± 0.593	-	96092435	96132454	-
2	105180000	105230000	50	4	171	0.19 ± 0.006	-4.47 ± 0.213	-	105802838	105852842	-
2	108550000	108570000	20	1	234	0.2	-4.19	-	109188601	109208592	*SNTG1*
2	119050000	119070000	20	1	96	0.18	-4.88	*ZFHX4*	119773491	119793306	*ZFHX4*
2	119430000	119450000	20	1	207	0.19	-4.35	-	120164442	120184442	-
2	132480000	132500000	20	1	110	0.2	-4.03	-	133345581	133365192	-
2	132540000	132560000	20	1	88	0.2	-4.08	-	133404207	133424203	-
2	132600000	132620000	20	1	101	0.2	-4.2	-	133478026	133498056	-
2	132650000	132670000	20	1	129	0.2	-4.17	-	133527683	133547686	-
2	133630000	133670000	40	3	155	0.18 ± 0.018	-4.91 ± 0.63	-	134504980	134545011	-
2	133820000	133840000	20	1	111	0.19	-4.45	-	134694509	134714515	-
3	1680000	1700000	20	1	120	0.2	-4.12	-	1706107	1726106	-
3	52600000	52620000	20	1	142	0.19	-4.43	-	53471438	53491438	-
3	52820000	52840000	20	1	160	0.2	-4.03	-	53690958	53710958	-
3	59920000	59950000	30	2	126	0.19 ± 0.003	-4.31 ± 0.09	*NKAIN2*	60613531	60643547	-
3	69730000	69750000	20	1	124	0.19	-4.48	*GRIK2*	70546301	70566301	*GRIK2*
3	69850000	69880000	30	2	135	0.2 ± 0.001	-4.12 ± 0.046	*GRIK2*	70666310	70696311	*GRIK2*

3	71740000	71770000	30	2	218	0.2 ± 0	-4.22 ± 0.016	-	72581753	72611648	-
3	82760000	82780000	20	1	198	0.19	-4.33	*BAI3*	83634484	83654483	-
3	83840000	83860000	20	1	192	0.2	-4.04	-	84739965	84759965	-
3	84040000	84070000	30	2	180	0.18 ± 0.01	-4.76 ± 0.347	-	84955044	84985013	-
3	84590000	84660000	70	6	156	0.19 ± 0.006	-4.36 ± 0.198	-	85510940	85580939	-
3	89190000	89210000	20	1	167	0.2	-4.28	*CSMD1*	89953067	89973065	-
3	110220000	110280000	60	5	110	0.2 ± 0.004	-4.23 ± 0.135	*EVA1A*	111068964	111128966	-
4	18220000	18270000	50	4	76	0.21 ± 0.006	-3.88 ± 0.208	*AFF2*	18329794	18379794	*AFF2*
4	18460000	18510000	50	4	54	0.21 ± 0.01	-3.76 ± 0.369	-	18578223	18628130	-
4	19730000	19750000	20	1	177	0.19	-4.41	-	20421152	20441152	-
4	19990000	20020000	30	2	236	0.19 ± 0.018	-4.45 ± 0.635	*NPY2R*	20681147	20711144	*NPY2R*
4	20200000	20240000	40	3	196	0.18 ± 0.008	-4.80 ± 0.269	*GUCY1A3*	20897359	20937251	*GUCY1A3*
4	20440000	20460000	20	1	180	0.19	-4.38	-	21137250	21157251	-
4	25730000	25750000	20	1	147	0.2	-4.2	-	26470754	26490763	-
4	27210000	27270000	60	5	189	0.18 ± 0.008	-4.78 ± 0.272	-	27953847	28013843	-
4	27700000	27730000	30	2	100	0.2 ± 0.001	-4.11 ± 0.027	-	28448563	28491310	-
4	27750000	27800000	50	4	154	0.2 ± 0.005	-4.11 ± 0.16	-	28511310	28561314	-
4	27800000	27820000	20	1	152	0.2	-4.03	-	28561314	28581315	-
4	27950000	27990000	40	3	175	0.19 ± 0.012	-4.34 ± 0.408	-	28711322	28751310	-
4	40500000	40530000	30	2	194	0.2 ± 0	-4.1 ± 0.003	-	41330667	41360667	-
4	41190000	41210000	20	1	112	0.2	-4.14	-	42031828	42051827	-
4	41440000	41460000	20	1	166	0.2	-4.06	-	42281885	42301886	-
4	47080000	47130000	50	4	352	0.16 ± 0.009	-5.5 ± 0.309	-	47987546	48037237	-
4	63130000	63150000	20	1	176	0.2	-4.07	-	63933853	63953853	-
4	63630000	63650000	20	1	255	0.18	-4.78	*SGCZ*	64433852	64453852	-
4	71460000	71510000	50	4	143	0.2 ± 0.01	-4.29 ± 0.364	-	72322171	72378964	-
4	71530000	71550000	20	1	103	0.2	-4.08	-	72402670	72422682	-
4	71570000	71590000	20	1	100	0.19	-4.32	-	72442687	72462683	-
4	71610000	71630000	20	1	211	0.19	-4.37	-	72481491	72501268	-
4	72120000	72150000	30	2	168	0.19 ± 0.008	-4.42 ± 0.278	-	72998015	73027972	-
4	74850000	74870000	20	1	141	0.19	-4.35	-	75813513	75833513	-

5	11530000	11550000	20	1	315	0.2	-4.25	-	12135842	12155842	-
5	19590000	19610000	20	1	112	0.2	-4.01	-	20294633	20314633	-
5	20330000	20350000	20	1	266	0.2	-4.01	-	21034381	21054389	-
5	31590000	31610000	20	1	214	0.2	-4.11	-	32326480	32346480	-
5	31820000	31870000	50	4	107	0.17 ± 0.031	-5.2 ± 1.108	-	32566762	32616762	-
5	40650000	40670000	20	1	154	0.19	-4.49	-	41418261	41438262	-
5	40950000	40970000	20	1	116	0.2	-4.13	-	41718279	41738279	-
5	51720000	51740000	20	1	88	0.15	-5.93	*AHNAK2*	51895684	51909028	-
5	41060000	41080000	20	1	116	0.2	-4.03	-	41828256	41848267	-
6	9820000	9860000	40	3	137	0.19 ± 0.011	-4.39 ± 0.383	-	10314423	10354423	-
6	12630000	12650000	20	1	224	0.2	-4.03	-	13065139	13085140	-
6	13300000	13330000	30	2	64	0.16 ± 0.009	-5.38 ± 0.306	*KCNMA1*	13734753	13764764	*KCNMA1*
7	30000	50000	20	1	80	0.19	-4.55	*NAB1*	48381	68381	*NAB1*
7	470000	500000	30	2	176	0.2 ± 0.002	-4.22 ± 0.069	*COL5A2*	492550	522549	*COL5A2*
7	7710000	7730000	20	1	77	0.18	-4.7	*TMEFF2*	8219061	8239061	-
7	8080000	8100000	20	1	101	0.2	-4.02	-	8588942	8608945	-
7	8160000	8180000	20	1	107	0.2	-4	-	8668747	8688747	-
7	14780000	14830000	50	4	70	0.2 ± 0.009	-4.02 ± 0.306	*TTN*	15343407	15393407	-
7	18510000	18550000	40	3	198	0.19 ± 0.012	-4.4 ± 0.421	*CERS6*	19106886	19146886	*CERS6*
7	19320000	19370000	50	3	84	0.16 ± 0.028	-5.59 ± 1.001	*SCN2A*	19916447	19966445	-
8	330000	350000	20	1	79	0.2	-4.2	-	334539	354538	-
8	9490000	9530000	40	3	132	0.2 ± 0.002	-4.07 ± 0.069	-	9581924	9621982	-
8	12170000	12190000	20	1	144	0.19	-4.58	-	13223917	13243917	-
8	12750000	12770000	20	1	147	0.2	-4.04	*BCAR3*	13826198	13846198	*DNTTIP2*
8	28400000	28420000	20	1	170	0.18	-4.71	-	29596909	29616910	-
8	28540000	28570000	30	2	74	0.19 ± 0.007	-4.61 ± 0.254	*TYW3*	29736834	29766834	*TYW3*
9	6460000	6490000	30	2	222	0.2 ± 0.002	-4.08 ± 0.062	-	6980087	7010087	-
9	23220000	23290000	70	6	102	0.16 ± 0.022	-5.58 ± 0.767	*PFN2, RNF13, COMMD2, WWTR1*	23853875	23923874	*WWTR1, RNF13, PFN2, COMMD2*

10	7630000	7650000	20	1	105	0.19	-4.37	*UNC13C*	8116641	8136640	-
10	15610000	15630000	20	1	153	0.2	-4.04	-	16123416	16143425	-
10	19590000	19640000	50	4	115	0.19 ± 0.014	-4.59 ± 0.486	*FES, FURIN, MFAP1, HYPK, SERINC4, PDIA3, CKMT1A*	20124637	20175340	*FES, SERINC4, PDIA3, FURIN, MFAP1, HYPK*
10	19750000	19770000	20	1	82	0.2	-4.1	*TP53BP1*	20286420	20306420	*TP53BP1*
11	2540000	2560000	20	1	65	0.2	-4.15	*NECAB2*	2609674	2629675	-
11	2580000	2600000	20	1	54	0.19	-4.34	*OSGIN1*	2649677	2669678	*OSGIN1*
11	3250000	3270000	20	1	66	0.2	-4.18	-	3872538	3892538	-
12	16560000	16580000	20	1	176	0.2	-4.01	-	16607821	16627821	-
13	17540000	17560000	20	1	52	0.19	-4.64	*CDC23*	18171476	18191474	*CDC23*
15	320000	350000	30	2	88	0.2 ± 0.007	-4.23 ± 0.258	*AL158801.1*	328627	358621	*UBE2L5P*
17	190000	230000	40	3	85	0.17 ± 0.005	-5.14 ± 0.175	-	687112	720224	-
17	10020000	10040000	20	1	101	0.2	-4	-	10504830	10524084	-
17	10350000	10370000	20	1	55	0.2	-4.16	*RALGPS1*	10833930	10863712	*RALGPS1*
18	30000	60000	30	2	58	0.19 ± 0.006	-4.38 ± 0.21	*ZNF302*	79637	109640	-
18	110000	130000	20	1	52	0.19	-4.61	*GSG1L2*	159529	179529	*GLP2R, GSG1L2*
18	150000	190000	40	3	86	0.2 ± 0.009	-4.2 ± 0.33	*GLP2R, GAS7*	199528	239528	*GLP2R, GAS7*
18	10910000	10930000	20	1	56	0.19	-4.51	*SLC16A5, ARMC7*	10732550	10752550	*SLC16A5, ARMC7, JPT1*
19	9430000	9460000	30	2	88	0.19 ± 0.012	-4.32 ± 0.408	*PAFAH1B1*	9455587	9485463	*PAFAH1B1*
22	230000	310000	80	7	119	0.17 ± 0.009	-5.22 ± 0.322	*ANTXR1, GKN2,*	238113	318051	*GKN2, ANTXR1,*

								BMP10,<br>*ARHGAP25*			*BMP10,*<br>*ARHGAP25*
22	340000	370000	30	2	60	0.18 ± 0.003	-4.84 ± 0.107	*TMEM230*	348052	378008	*SVCT2,*<br>*TMEM230*
22	440000	500000	60	5	87	0.16 ± 0.008	-5.52 ± 0.289	*PROM2,*<br>*DPYSL2*	447995	508112	*PROM2,*<br>*DPYSL2*
22	570000	720000	150	10	56	0.17 ± 0.018	-5.03 ± 0.641	*PPP2R2A,*<br>*EBF2*	578106	728044	*EBF2,*<br>*PPP2R2A*
22	740000	770000	30	2	76	0.18 ± 0.017	-4.86 ± 0.612	-	748044	778044	-
22	790000	830000	40	3	76	0.19 ± 0.005	-4.58 ± 0.173	*CDCA2*	798042	838042	*KCTD9,*<br>*GnRH-I*
22	870000	960000	90	7	74	0.19 ± 0.009	-4.63 ± 0.314	*KCTD9,*<br>*GNRH1,*<br>*DOCK5,*<br>*NEFL,*<br>*NEFM*	878042	968083	*NEFL,*<br>*NEFM*
22	990000	1040000	50	4	76	0.18 ± 0.013	-4.75 ± 0.453	-	998083	1048083	-
22	1060000	1090000	30	2	54	0.16 ± 0.001	-5.58 ± 0.051	-	1068084	1098084	-
22	1090000	1180000	90	8	63	0.17 ± 0.018	-5.27 ± 0.64	*STC1,*<br>*SLC25A37*	1098084	1188031	*STC1*
22	1200000	1250000	50	4	133	0.18 ± 0.007	-4.76 ± 0.238	*ENTPD4,*<br>*LOXL2,*<br>*R3HCC1*	1208020	1258019	*LOXL2*
22	1290000	1330000	40	3	115	0.19 ± 0.008	-4.65 ± 0.271	*RHOBTB2*	1301346	1340487	-
22	1380000	1410000	30	2	146	0.2 ± 0.001	-4.06 ± 0.026	*DOK2,*<br>*GFRA2*	1405569	1435635	*GFRA2*
22	1420000	1480000	60	5	174	0.19 ± 0.01	-4.4 ± 0.353	-	1445616	1506489	-
22	1510000	1610000	100	9	132	0.19 ± 0.011	-4.37 ± 0.402	*LZTS1,*<br>*ATP6V1B2,*<br>*SLC18A1,*<br>*MAK16,*	1536501	1636436	*LZTS1,*<br>*SLC18A1,*<br>*MAK16,*<br>*TTI2,*<br>*RNF122,*

								TTI2, RNF122			*DUSP26, ATP6V1B2*
22	1710000	1740000	30	2	136	0.2 ± 0.006	-4.27 ± 0.22	-	1736535	1766538	-
22	1760000	1780000	20	1	111	0.2	-4.12	-	1786538	1806536	*UNC5D*
22	2080000	2100000	20	1	87	0.19	-4.61	-	2106404	2126412	-
22	2120000	2190000	70	6	113	0.19 ± 0.011	-4.34 ± 0.403	*ERLIN2, PROSC*	2146417	2215300	*ZNF703, PLPBP, ERLIN2*
22	2270000	2330000	60	5	90	0.17 ± 0.017	-5.03 ± 0.612	*LSM1, BAG4, DDHD2, PPAPDC1B, WHSC1L1, LETM2*	2294998	2355935	*DDHD2, BAG4, LETM2, NSD3, PLPP5*
22	2350000	2560000	210	20	95	0.19 ± 0.019	-4.63 ± 0.653	*TACC1, PLEKHA2, TM2D2, ADAM9, INDOL1, ZMAT4, SFRP1*	2376153	2590429	*TACC1, TM2D2, C8orf4, PLEKHA2, ADAM9, ZMAT4,*
22	2570000	2600000	30	2	60	0.17 ± 0.002	-5.09 ± 0.087	*GOLGA7, GINS4, AGPAT6*	2600431	2630057	*SFRP1, GOLGA7*
22	2620000	2660000	40	3	65	0.19 ± 0.009	-4.65 ± 0.31	*GKN2*	2650063	2690060	*ANK1, gga-mir-2188*
23	100000	120000	20	1	88	0.2	-4.16	*RPS6KA1*	103515	123509	*RPS6KA*
23	5450000	5510000	60	5	104	0.17 ± 0.034	-5.26 ± 1.217	*NT5C1A, HPCAL4, TRIT1, MYCL, MFSD2A,*	5511860	5571894	*NT5C1A, MYCL, MFSD2A, TRIT1,*

								CAP1, NIPAL3			*CAP1, HPCAL4*
23	5700000	5720000	20	1	58	0.19	-4.46	*RCAN3*	5761781	5781785	*NIPAL3, RCAN3*
26	100000	120000	20	1	66	0.2	-4.11	*SRPK1, SLC26A8*	104471	124471	*SRPK1, SLC26A8*
26	5190000	5240000	50	4	104	0.2 ± 0.024	-3.98 ± 0.861	*PRELP, OPTC*	5155940	5205938	*PRELP, OPTC*
