# Supplementary material for: Whole-Genome Resequencing of Red Junglefowl and Indigenous Village Chicken Reveal New Insights on the Genome Dynamics of the Species
Source: Front Genet. 2018 Jul 20;9:264. doi: 10.3389/fgene.2018.00264 (PMC6062655; doi:10.3389/fgene.2018.00264)
Supplement: Supplementary file 5 [file Table_5.PDF]

**Table S5** | Candidate selective sweep regions detected in Ethiopian domestic chicken population using $H_p$. The **Ggal** is the reference genome annotation for the *Galgal* version 4 or 5, **Ln** is length in kilobase, **nWd** is the number of windows analysed for the sweep region.

| **Chr** | **Ggal4_start** | **Ggal4_stop** | **Ln** | **nWd** | **Total SNP** | **mean ($H_p$)** | **mean Z($H_p$)** | **Ggal4_gene** | **Ggal5_start** | **Ggal5_stop** | **Ggal5_gene** |
|---|---|---|---|---|---|---|---|---|---|---|---|
| 1 | 25440000 | 25460000 | 20 | 1 | 78 | 0.1 | -4.18 | - | 25502468 | 25522471 | - |
| 1 | 32430000 | 32450000 | 20 | 1 | 146 | 0.09 | -4.34 | - | 32471039 | 32491038 | |
| 1 | 32480000 | 32510000 | 30 | 2 | 130 | 0.09 ± 0.008 | -4.27 ± 0.164 | - | 32521038 | 32551568 | |
| 1 | 32580000 | 32620000 | 40 | 3 | 112 | 0.07 ± 0.025 | -4.65 ± 0.497 | - | 32621786 | 32661772 | |
| 1 | 58890000 | 58910000 | 20 | 1 | 194 | 0.1 | -4.1 | - | 58796189 | 58816190 | |
| 1 | 82370000 | 82390000 | 20 | 1 | 121 | 0.1 | -4.03 | - | 82949734 | 82969734 | |
| 1 | 83290000 | 83310000 | 20 | 1 | 252 | 0.1 | -4.07 | - | 83885475 | 83905475 | |
| 1 | 85220000 | 85240000 | 20 | 1 | 268 | 0.1 | -4.03 | - | 85815617 | 85835617 | |
| 1 | 115620000 | 115640000 | 20 | 1 | 202 | 0.1 | -4.13 | *IL1RAPL1* | 116133430 | 116153430 | *IL1RAPL1* |
| 1 | 116020000 | 116120000 | 100 | 8 | 64 | 0.09 ± 0.007 | -4.25 ± 0.14 | - | 116533502 | 116633508 | *IL1RAPL1* |
| 1 | 116200000 | 116240000 | 40 | 3 | 81 | 0.1 ± 0.008 | -4.2 ± 0.161 | - | 116713508 | 116753509 | - |
| 1 | 118940000 | 118970000 | 30 | 2 | 110 | 0.09 ± 0.012 | -4.36 ± 0.239 | - | 119499993 | 119529993 | |
| 1 | 145950000 | 145970000 | 20 | 1 | 136 | 0.1 | -4.02 | *GPC6* | 146719329 | 146739329 | *GPC6* |
| 1 | 190070000 | 190110000 | 40 | 3 | 205 | 0.09 ± 0.012 | -4.23 ± 0.227 | - | 190937229 | 190977200 | |
| 2 | 9110000 | 9130000 | 20 | 1 | 145 | 0.08 | -4.48 | *PTPRN2* | 9142747 | 9162747 | *PTPRN2* |
| 2 | 60840000 | 60880000 | 40 | 3 | 173 | 0.08 ± 0.024 | -4.58 ± 0.477 | - | 60957242 | 60997244 | |
| 2 | 61100000 | 61130000 | 30 | 2 | 228 | 0.1 ± 0.004 | -4.1 ± 0.081 | - | 61217342 | 61247344 | |
| 2 | 70360000 | 70380000 | 20 | 1 | 133 | 0.11 | -4 | - | 70544869 | 70564874 | |
| 2 | 70450000 | 70500000 | 50 | 4 | 138 | 0.1 ± 0.005 | -4.14 ± 0.092 | - | 70632976 | 70682149 | |
| 2 | 70650000 | 70680000 | 30 | 2 | 196 | 0.1 ± 0.003 | -4.06 ± 0.06 | - | 70831053 | 70861025 | - |
| 2 | 70730000 | 70750000 | 20 | 1 | 78 | 0.1 | -4.13 | - | 70911434 | 70931388 | - |
| 2 | 70840000 | 70880000 | 40 | 3 | 131 | 0.1 ± 0.004 | -4.16 ± 0.083 | - | 71021440 | 71061441 | - |
| 2 | 77810000 | 77860000 | 50 | 4 | 116 | 0.1 ± 0.005 | -4.05 ± 0.1 | *CTNND2* | 78056727 | 78107080 | *CTNND2* |
| 2 | 78310000 | 78340000 | 30 | 2 | 117 | 0.09 ± 0.023 | -4.38 ± 0.455 | - | 78554635 | 78584572 | - |
| 2 | 118570000 | 118590000 | 20 | 1 | 98 | 0.09 | -4.3 | - | 119293576 | 119313578 | |
| 2 | 121660000 | 121700000 | 40 | 3 | 319 | 0.1 ± 0.006 | -4.09 ± 0.121 | - | 122409571 | 122449574 | |
| 2 | 138730000 | 138750000 | 20 | 1 | 124 | 0.1 | -4.05 | - | 139258589 | 139278588 | |

| | | | | | | | | | | | |
|---|---|---|---|---|---|---|---|---|---|---|---|
| 2 | 139930000 | 139950000 | 20 | 1 | 105 | 0.09 | -4.32 | - | 140458643 | 140478642 | |
| 2 | 139970000 | 140020000 | 50 | 4 | 141 | 0.06 ± 0.037 | -4.85 ± 0.728 | - | 140498641 | 140551586 | |
| 2 | 146720000 | 146740000 | 20 | 1 | 109 | 0.03 | -5.4 | - | 147254792 | 147274793 | - |
| 3 | 23760000 | 23780000 | 20 | 1 | 86 | 0.11 | -4 | - | 24475569 | 24495569 | |
| 3 | 54860000 | 54880000 | 20 | 1 | 99 | 0.1 | -4.21 | *AHI1* | 55762675 | 55782675 | *AHI1* |
| 3 | 60820000 | 60840000 | 20 | 1 | 108 | 0.09 | -4.25 | - | 61527746 | 61547720 | - |
| 3 | 78890000 | 78910000 | 20 | 1 | 280 | 0.09 | -4.26 | *HMGN3* | 79759035 | 79779035 | *HMGN3* |
| 3 | 84850000 | 84870000 | 20 | 1 | 58 | 0.1 | -4.18 | *EYS* | 85768126 | 85788126 | - |
| 3 | 85680000 | 85720000 | 40 | 3 | 129 | 0.1 ± 0.005 | -4.19 ± 0.09 | *KHDRBS2* | 86598181 | 86638181 | *KHDRBS2* |
| 3 | 93670000 | 93690000 | 20 | 1 | 162 | 0.09 | -4.21 | - | 94457822 | 94477823 | |
| 3 | 101900000 | 101950000 | 50 | 4 | 211 | 0.09 ± 0.008 | -4.29 ± 0.156 | *APOB* | 102677018 | 102726963 | *APOB* |
| 3 | 102150000 | 102210000 | 60 | 5 | 86 | 0.09 ± 0.004 | -4.35 ± 0.087 | - | 102948024 | 103007976 | |
| 3 | 102260000 | 102290000 | 30 | 2 | 123 | 0.1 ± 0.001 | -4.08 ± 0.028 | - | 103057981 | 103087981 | |
| 3 | 102360000 | 102470000 | 110 | 10 | 83 | 0.1 ± 0.007 | -4.17 ± 0.129 | *gga-mir-6678* | 103157991 | 103267894 | *gga-mir-6678* |
| 3 | 102490000 | 102520000 | 30 | 2 | 67 | 0.09 ± 0.006 | -4.28 ± 0.126 | - | 103287894 | 103317895 | - |
| 3 | 102570000 | 102660000 | 90 | 8 | 124 | 0.08 ± 0.015 | -4.45 ± 0.299 | - | 103367896 | 103457512 | |
| 3 | 102720000 | 102870000 | 150 | 12 | 91 | 0.06 ± 0.033 | -4.96 ± 0.651 | - | 103517529 | 103667817 | |
| 4 | 7890000 | 7970000 | 80 | 7 | 105 | 0.08 ± 0.02 | -4.51 ± 0.392 | - | 7949811 | 8029812 | |
| 4 | 27110000 | 27130000 | 20 | 1 | 129 | 0.09 | -4.23 | - | 27853766 | 27873764 | |
| 4 | 38620000 | 38660000 | 40 | 3 | 213 | 0.08 ± 0.018 | -4.41 ± 0.346 | *TACR3* | 39449745 | 39489745 | *TACR3* |
| 4 | 75220000 | 75240000 | 20 | 1 | 107 | 0.09 | -4.24 | - | 76190363 | 76210367 | - |
| 4 | 75400000 | 75420000 | 20 | 1 | 108 | 0.09 | -4.35 | *LCORL* | 76373927 | 76391801 | *LCORL* |
| 4 | 77130000 | 77180000 | 50 | 4 | 154 | 0.04 ± 0.04 | -5.21 ± 0.778 | - | 78118133 | 78168138 | |
| 4 | 77410000 | 77490000 | 80 | 7 | 131 | 0.1 ± 0.008 | -4.07 ± 0.163 | - | 78407934 | 78487939 | |
| 4 | 81040000 | 81060000 | 20 | 1 | 205 | 0.1 | -4.11 | - | 82050389 | 82070389 | |
| 5 | 40060000 | 40110000 | 50 | 4 | 166 | 0.01 ± 0.015 | -5.8 ± 0.289 | *TSHR, GTF2A1* | 40828747 | 40878736 | *TSHR, GTF2A1* |
| 5 | 10780000 | 10800000 | 20 | 1 | 123 | 0.1 | -4.07 | *SOX6* | 11375407 | 11395406 | |
| 5 | 21810000 | 21850000 | 40 | 3 | 66 | 0.05 ± 0.021 | -4.99 ± 0.42 | - | 22511835 | 22551887 | |
| 5 | 30440000 | 30490000 | 50 | 4 | 81 | 0.09 ± 0.007 | -4.22 ± 0.127 | - | 31176457 | 31226458 | |

| | | | | | | | | | | | |
|---|---|---|---|---|---|---|---|---|---|---|---|
| 5 | 39900000 | 39920000 | 20 | 1 | 131 | 0.1 | -4.14 | - | 40668742 | 40688742 | |
| 5 | 40250000 | 40310000 | 60 | 5 | 166 | 0.07 ± 0.022 | -4.71 ± 0.438 | - | 41018589 | 41078590 | |
| 5 | 41050000 | 41140000 | 90 | 8 | 92 | 0.1 ± 0.027 | -4.15 ± 0.537 | - | 41818256 | 41908264 | |
| 5 | 55190000 | 55210000 | 20 | 1 | 338 | 0.1 | -4.15 | *C14orf37* | 55431566 | 55451566 | *C14orf37* |
| 7 | 7770000 | 7790000 | 20 | 1 | 95 | 0.1 | -4.04 | *TMEFF2* | 8279061 | 8299061 | - |
| 7 | 7860000 | 7920000 | 60 | 5 | 96 | 0.13 ± 0.054 | -4.0 ± 1.051 | - | 8369039 | 8429044 | |
| 7 | 8070000 | 8090000 | 20 | 1 | 100 | 0.08 | -4.45 | - | 8578942 | 8598945 | |
| 7 | 15860000 | 15920000 | 60 | 5 | 260 | 0.08 ± 0.01 | -4.43 ± 0.204 | *KIAA1715* | 16423183 | 16483186 | *LNPK* |
| 7 | 16480000 | 16530000 | 50 | 4 | 183 | 0.06 ± 0.009 | -4.86 ± 0.18 | *OLA1* | 17047182 | 17097167 | - |
| 7 | 17880000 | 17900000 | 20 | 1 | 229 | 0.1 | -4.1 | *MYO3B* | 18477165 | 18497165 | *MYO3B* |
| 8 | 10000 | 70000 | 60 | 5 | 100 | 0.1 ± 0.005 | -4.16 ± 0.09 | *AMY2A* | 14572 | 74532 | - |
| 8 | 110000 | 140000 | 30 | 2 | 53 | 0.1 ± 0.001 | -4.03 ± 0.021 | - | 114536 | 144536 | |
| 8 | 160000 | 270000 | 110 | 10 | 70 | 0.09 ± 0.015 | -4.34 ± 0.287 | - | 164536 | 274537 | |
| 8 | 370000 | 390000 | 20 | 1 | 53 | 0.1 | -4.04 | - | 374538 | 394538 | |
| 8 | 420000 | 630000 | 210 | 19 | 116 | 0.09 ± 0.003 | -4.29 ± 0.055 | - | 424781 | 634785 | |
| 8 | 8740000 | 8760000 | 20 | 1 | 221 | 0.03 | -5.46 | - | 8824776 | 8844776 | |
| 8 | 8810000 | 8830000 | 20 | 1 | 299 | 0.1 | -4.03 | - | 8894776 | 8914776 | |
| 8 | 9050000 | 9130000 | 80 | 7 | 125 | 0.08 ± 0.013 | -4.58 ± 0.262 | - | 9138797 | 9221862 | |
| 8 | 9420000 | 9450000 | 30 | 2 | 150 | 0.08 ± 0.01 | -4.45 ± 0.188 | - | 9511843 | 9541920 | - |
| 9 | 9630000 | 9680000 | 50 | 4 | 144 | 0.07 ± 0.012 | -4.63 ± 0.23 | *SLC16A14* | 10149261 | 10199462 | *SLC16A14* |
| 9 | 9740000 | 9790000 | 50 | 4 | 185 | 0.09 ± 0.013 | -4.32 ± 0.255 | *RNF7, GRK7, ATP1B3* | 10259300 | 10309300 | *RNF7, GRK7, ATP1B3* |
| 9 | 9870000 | 9900000 | 30 | 2 | 213 | 0.07 ± 0.019 | -4.67 ± 0.379 | *GK5* | 10389300 | 10433014 | *GK5* |
| 9 | 10960000 | 10990000 | 30 | 2 | 194 | 0.09 ± 0.002 | -4.31 ± 0.035 | *PLOD5* | 11499478 | 11529475 | *PLOD2* |
| 9 | 11860000 | 11880000 | 20 | 1 | 135 | 0.06 | -4.8 | *AGTR1* | 12410602 | 12430603 | *AGTR1* |
| 12 | 4350000 | 4420000 | 70 | 6 | 161 | 0.08 ± 0.01 | -4.55 ± 0.195 | *ATG7, HRH1* | 4352528 | 4422400 | *HRH1, ATG7* |
| 12 | 10490000 | 10520000 | 30 | 2 | 209 | 0.08 ± 0.018 | -4.43 ± 0.357 | *SLC41A3* | 10513118 | 10543121 | *SLC41A3* |

| | | | | | | | | | | | |
|---|---|---|---|---|---|---|---|---|---|---|---|
| 23 | 5460000 | 5490000 | 30 | 2 | 67 | 0.1 ± 0.013 | -4.2 ± 0.254 | *HPCAL4, TRIT1, MYCL* | 5521860 | 5551860 | *HPCAL4, MYCL, TRIT1* |
| 24 | 6140000 | 6160000 | 20 | 1 | 199 | 0.08 | -4.41 | *BCDO2* | 6117101 | 6137115 | *BCO2* |
