# Supplementary material for: Whole-Genome Resequencing of Red Junglefowl and Indigenous Village Chicken Reveal New Insights on the Genome Dynamics of the Species
Source: Front Genet. 2018 Jul 20;9:264. doi: 10.3389/fgene.2018.00264 (PMC6062655; doi:10.3389/fgene.2018.00264)
Supplement: Supplementary file 6 [file Table_6.PDF]

Table S6 | Candidate selective sweep regions detected in Saudi Arabian domestic chicken population using H_p. The **Ggal** is the reference genome annotation for the *Galgal* version 4 or 5, **Ln** is length in kilobase, **nWd** is the number of windows analysed for the sweep region.

Chr	Ggal4_start	Ggal4_stop	Ln	nWd	Total SNP	mean (H_p)	mean Z(H_p)	Ggal4_gene	Ggal5_start	Ggal5_stop	Ggal5_gene
1	8510000	8540000	30	2	54	0.06 ± 0.072	-6.19 ± 1.48	*SEMA3A*	8655463	8685324	-
1	9050000	9070000	20	1	76	0.15	-4.24	-	9195890	9215896	-
1	20820000	20850000	30	2	125	0.14 ± 0.008	-4.47 ± 0.171	*GRM8*	21048712	21078728	*GRM8*
1	20960000	21000000	40	3	101	0.13 ± 0.005	-4.77 ± 0.107	*GRM8*	21188645	21228640	*GRM8*
1	42470000	42490000	20	1	50	0.15	-4.36	-	42587204	42607205	-
1	43140000	43170000	30	2	136	0.11 ± 0.014	-5.05 ± 0.296	-	43256829	43286830	-
1	46610000	46640000	30	2	176	0.15 ± 0.012	-4.23 ± 0.247	*ANKS1B*	46791047	46821047	*ANKS1B*
1	55950000	55990000	40	3	147	0.14 ± 0.017	-4.46 ± 0.343	*TBXAS1*	55852659	55892659	*TBXAS1*
1	56010000	56040000	30	2	167	0.14 ± 0.02	-4.5 ± 0.415	*TBXAS1*	55912659	55942660	*TBXAS1*
1	56730000	56750000	20	1	140	0.16	-4.01	*AKR1D1*	56635124	56655122	*AKR1D1*
1	58390000	58430000	40	3	172	0.14 ± 0.001	-4.52 ± 0.03	*MTPN*	58295164	58335165	*MTPN*
1	58680000	58730000	50	4	124	0.1 ± 0.06	-5.28 ± 1.244	-	58585656	58635815	-
1	58890000	58910000	20	1	187	0.13	-4.74	-	58796189	58816190	-
1	65060000	65080000	20	1	108	0.16	-4.05	*PDE3A*	64961251	64981245	*PDE3A*
1	66600000	66620000	20	1	198	0.15	-4.28	*ETNK1*	66506397	66526399	*ETNK1*
1	70150000	70180000	30	2	167	0.14 ± 0.029	-4.5 ± 0.593	*PHF21B*	70052471	70082363	-
1	71980000	72020000	40	3	238	0.12 ± 0.027	-4.97 ± 0.565	*MANSC1*	71895923	71935889	*BORCS5*
1	72040000	72110000	70	6	138	0.13 ± 0.05	-4.79 ± 1.04	*BORCS5, DUSP16*	71955872	72023023	*BORCS5*
1	72130000	72210000	80	7	132	0.11 ± 0.054	-5.1 ± 1.121	*CREBL2, GPR19*	72043004	72122859	*GPR19, CREBL2*
1	75410000	75430000	20	1	81	0.16	-4.16	*AICDA, MFAP5*	75957200	75977217	*AICDA, MFAP5*
1	75460000	75500000	40	3	78	0.16 ± 0.003	-4.19 ± 0.068	*RIMKLB*	76007210	76047240	*RIMKLB*
1	82380000	82400000	20	1	94	0.11	-5.04	*DRD3*	82959734	82979735	*DRD3*
1	96210000	96230000	20	1	50	0.16	-4.09	-	96865482	96885484	-
1	112840000	112860000	20	1	142	0.12	-4.83	-	113521411	113541411	-

1	122810000	122880000	70	6	156	0.13 ± 0.061	-4.75 ± 1.252	*TLR7, PRPS2*	123361389	123431387	*TLR7, PRPS2*
1	123700000	123770000	70	6	113	0.15 ± 0.036	-4.25 ± 0.742	*ARHGAP6*	124265475	124335470	-
1	126500000	126580000	80	6	72	0.13 ± 0.022	-4.79 ± 0.447	-	127142678	127222718	-
1	126910000	126940000	30	2	106	0.15 ± 0.003	-4.22 ± 0.053	-	127574901	127605626	-
1	136170000	136190000	20	1	136	0.12	-4.98	*Sep-10*	136827553	136847553	-
1	139970000	139990000	20	1	53	0.16	-4.08	-	140674252	140694246	-
1	140980000	141020000	40	3	112	0.14 ± 0.022	-4.46 ± 0.453	-	141712197	141752274	-
1	141160000	141180000	20	1	86	0.16	-4.04	-	141891998	141911996	-
1	156100000	156120000	20	1	164	0.14	-4.45	*DACH1*	156917585	156936607	*DACH1*
1	160120000	160140000	20	1	67	0.03	-6.79	-	160950617	160970616	-
1	167780000	167830000	50	4	212	0.17 ± 0.016	-4.0 ± 0.321	-	168477601	168527595	-
1	167930000	167950000	20	1	208	0.16	-4.06	*SUCLA2*	168627590	168647590	*SUCLA2*
1	178290000	178360000	70	6	128	0.13 ± 0.031	-4.67 ± 0.65	*ZMYM2*	179066742	179136744	-
1	190030000	190100000	70	6	114	0.16 ± 0.029	-4.02 ± 0.59	-	190897239	190967194	-
1	190120000	190140000	20	1	70	0.09	-5.54	-	190987202	191007202	-
2	2250000	2280000	30	2	260	0.14 ± 0.019	-4.42 ± 0.4	*OBSCN*	2286387	2316389	*OBSCN*
2	8230000	8260000	30	2	82	0.1 ± 0.069	-5.34 ± 1.426	-	8262165	8292803	-
2	18920000	18940000	20	1	96	0.15	-4.28	-	19065339	19085341	-
2	19950000	19970000	20	1	167	0.14	-4.53	*RSU1*	20095967	20115967	*RSU1*
2	21680000	21710000	30	2	250	0.14 ± 0.002	-4.58 ± 0.037	*CDK14*	21825360	21855358	-
2	24200000	24220000	20	1	152	0.16	-4.03	*DYNC1I1*	24099944	24119944	*DYNC1I1*
2	26210000	26270000	60	5	111	0.12 ± 0.032	-4.93 ± 0.661	*PHF14*	26135153	26195154	*PHF14*
2	26530000	26580000	50	4	91	0.12 ± 0.038	-4.85 ± 0.796	*THSD7A*	26451931	26501934	*THSD7A*
2	28190000	28220000	30	2	74	0.15 ± 0.015	-4.24 ± 0.312	*MEOX2*	28112043	28142109	*MEOX2*
2	28250000	28290000	40	3	91	0.14 ± 0.023	-4.5 ± 0.483	*5S_rRNA*	28171924	28211919	*5S_rRNA*
2	35850000	35890000	40	3	56	0.16 ± 0.01	-4.19 ± 0.209	-	35412150	35452144	-
2	35910000	35930000	20	1	108	0.14	-4.52	-	35472144	35492144	-
2	39300000	39320000	20	1	141	0.15	-4.25	-	38860337	38880391	-
2	39360000	39380000	20	1	178	0.14	-4.56	*CMC1*	38920389	38940389	*CMC1*
2	41420000	41460000	40	3	134	0.1 ± 0.032	-5.3 ± 0.653	*ABHD5*	40977323	41017324	*ABHD5*

2	43490000	43520000	30	2	320	0.16 ± 0.009	-4.19 ± 0.189	*LARS2*	43064488	43094486	*LARS2*
2	55240000	55260000	20	1	136	0.15	-4.37	*TNS3*	55313328	55333328	*TNS3*
2	58240000	58270000	30	2	128	0.14 ± 0.003	-4.46 ± 0.053	-	58357511	58387510	-
2	58560000	58580000	20	1	103	0.09	-5.46	-	58678019	58698019	-
2	60860000	60900000	40	3	171	0.14 ± 0.02	-4.45 ± 0.405	-	60977244	61017243	-
2	63110000	63140000	30	2	203	0.15 ± 0.003	-4.25 ± 0.063	*C1orf145*	63235842	63265843	-
2	65860000	65900000	40	3	148	0.12 ± 0.032	-4.91 ± 0.656	-	66000543	66040615	-
2	70850000	70870000	20	1	56	0.15	-4.22	-	71031438	71051451	-
2	74290000	74310000	20	1	97	0.16	-4.18	*CDH18*	74523330	74543335	*CDH18*
2	75140000	75280000	140	13	159	0.13 ± 0.029	-4.8 ± 0.605	-	75375947	75512081	-
2	81900000	81920000	20	1	51	0.15	-4.25	-	82140679	82160679	-
2	83160000	83200000	40	3	120	0.13 ± 0.028	-4.77 ± 0.588	*FHOD3*	83402038	83442039	*FHOD3*
2	83460000	83490000	30	2	82	0.14 ± 0.007	-4.53 ± 0.141	*FHOD3*	83702035	83748704	*FHOD3*
2	86240000	86260000	20	1	128	0.15	-4.22	-	86515413	86535412	-
2	86340000	86440000	100	9	106	0.1 ± 0.036	-5.36 ± 0.752	-	86615401	86715403	-
2	92550000	92580000	30	2	144	0.15 ± 0.007	-4.29 ± 0.145	*NETO1, MIR1803*	92866622	92896627	*gga-mir-1803*
2	95760000	95780000	20	1	74	0.16	-4.03	-	96102435	96122454	-
2	103040000	103060000	20	1	268	0.12	-4.9	*IMPACT*	103676119	103696119	*HRH4, IMPACT*
2	104610000	104640000	30	2	144	0.15 ± 0.017	-4.32 ± 0.344	-	105245091	105275091	-
2	121820000	121840000	20	1	245	0.15	-4.24	-	122569578	122589578	-
2	122030000	122090000	60	5	249	0.17 ± 0.033	-4.0 ± 0.687	-	122779584	122839580	*RALYL*
2	125030000	125050000	20	1	96	0.06	-6.14	-	125800217	125820219	-
2	138730000	138760000	30	2	155	0.14 ± 0.009	-4.59 ± 0.191	-	139258589	139288588	-
2	139130000	139150000	20	1	180	0.14	-4.42	-	139658602	139678603	-
2	140870000	140970000	100	8	180	0.11 ± 0.018	-5.08 ± 0.382	*EFR3A*	141401212	141501204	*HHLA1, EFR3A*
2	141000000	141070000	70	6	123	0.12 ± 0.057	-4.97 ± 1.182	*KCNQ3*	141531206	141601206	*KCNQ3*
2	146610000	146630000	20	1	85	0.15	-4.38	-	147144279	147164279	-
2	146690000	146800000	110	10	101	0.07 ± 0.052	-6.03 ± 1.067	-	147224789	147334917	-

2	148640000	148690000	50	4	208	0.14 ± 0.019	-4.51 ± 0.384	*SCRIB*	149359515	149400945	*SCRIB*
3	20610000	20630000	20	1	84	0.16	-4.1	*PROX1*	21297727	21317728	*PROX1*
3	17530000	17590000	60	5	163	0.11 ± 0.034	-5.14 ± 0.698	-	18200441	18260441	-
3	18920000	18940000	20	1	75	0.15	-4.23	-	19607982	19627969	-
3	23740000	23760000	20	1	187	0.16	-4.18	-	24455568	24475569	-
3	33710000	33760000	50	4	100	0.04 ± 0.05	-6.49 ± 1.027	*KIF26B*	34481501	34531541	*KIF26B*
3	45690000	45720000	30	2	135	0.14 ± 0.027	-4.44 ± 0.564	-	46542544	46572542	-
3	51940000	51970000	30	2	166	0.14 ± 0.008	-4.48 ± 0.166	-	52811308	52841309	-
3	52710000	52800000	90	5	64	0.14 ± 0.017	-4.45 ± 0.351	-	53580956	53670958	-
3	52830000	52850000	20	1	56	0.15	-4.25	-	53700958	53720958	-
3	52910000	52930000	20	1	163	0.14	-4.43	-	53781049	53801048	-
3	60860000	60880000	20	1	58	0.16	-4.04	-	61567721	61587720	-
3	63510000	63530000	20	1	81	0.14	-4.47	*FRK*	64251464	64271466	*FRK*
3	69200000	69220000	20	1	118	0.15	-4.32	-	70016118	70036036	-
3	71210000	71250000	40	3	160	0.15 ± 0.011	-4.29 ± 0.221	-	72018345	72058345	-
3	71470000	71490000	20	1	124	0.15	-4.37	-	72311718	72331718	-
3	72460000	72480000	20	1	68	0.12	-4.99	-	73301240	73321239	-
3	72500000	72530000	30	2	126	0.15 ± 0.011	-4.27 ± 0.235	-	73341244	73369976	-
3	74500000	74520000	20	1	225	0.16	-4.12	*MAP3K7*	75366769	75386750	*MAP3K7*
3	78360000	78380000	20	1	95	0.14	-4.45	-	79231914	79249094	-
3	78890000	78910000	20	1	211	0.16	-4.1	*HMGN3*	79759035	79779035	*HMGN3*
3	79180000	79230000	50	4	124	0.1 ± 0.045	-5.28 ± 0.932	-	80048866	80098866	-
3	79910000	79950000	40	3	217	0.1 ± 0.05	-5.25 ± 1.041	*MYO6*	80786402	80826401	*MYO6*
3	80180000	80200000	20	1	210	0.15	-4.39	*TMEM30A, COX7A2*	81056354	81076353	*COX7A2, TMEM30A*
3	80740000	80760000	20	1	81	0.15	-4.36	-	81614455	81634456	-
3	81610000	81630000	20	1	260	0.1	-5.32	*RIMS1*	82484657	82504658	*RIMS1*
3	83220000	83320000	100	8	98	0.13 ± 0.009	-4.63 ± 0.177	-	84094372	84194235	-
3	83370000	83450000	80	7	76	0.12 ± 0.01	-4.98 ± 0.216	-	84258077	84338083	-
3	83500000	83520000	20	1	80	0.15	-4.2	-	84388055	84408054	-
3	83590000	83650000	60	5	73	0.11 ± 0.009	-5.19 ± 0.192	-	84478052	84538052	-

3	84050000	84070000	20	1	122	0.16	-4.03	-	84965043	84985013	-
3	87580000	87630000	50	4	202	0.14 ± 0.023	-4.53 ± 0.476	*GCLC*	88524115	88574095	*GCLC*
3	93120000	93140000	20	1	160	0.16	-4.1	-	93907611	93927611	-
3	93160000	93240000	80	6	132	0.11 ± 0.028	-5.07 ± 0.581	*TSSC1, TRAPPC12*	93947611	94027610	*EIPR1, TRAPPC12*
3	97010000	97050000	40	3	151	0.15 ± 0.013	-4.2 ± 0.276	*LPIN1*	97890345	97930345	*LPIN1*
3	101360000	101390000	30	2	154	0.14 ± 0.026	-4.46 ± 0.532	*RHOB*	102134978	102164994	*RHOB*
3	102690000	102760000	70	6	118	0.14 ± 0.036	-4.41 ± 0.749	-	103487514	103557731	-
4	6960000	6990000	30	2	120	0.13 ± 0.052	-4.79 ± 1.074	-	7019690	7049689	-
4	26410000	26430000	20	1	239	0.16	-4.17	-	27150530	27174313	-
4	27110000	27140000	30	2	123	0.14 ± 0.01	-4.55 ± 0.21	-	27853766	27883764	-
4	27160000	27190000	30	2	80	0.16 ± 0.002	-4.05 ± 0.043	-	27903762	27933845	-
4	27380000	27470000	90	7	99	0.09 ± 0.032	-5.45 ± 0.656	-	28123845	28218670	-
4	27860000	27910000	50	4	123	0.15 ± 0.016	-4.29 ± 0.331	-	28621318	28671317	-
4	28120000	28240000	120	11	123	0.12 ± 0.084	-4.93 ± 1.743	*PCDH18*	28881313	29001315	*PCDH18*
4	32790000	32820000	30	2	163	0.11 ± 0.059	-5.19 ± 1.214	-	33597841	33627839	*FAM160A1*
4	41190000	41210000	20	1	53	0.16	-4.02	-	42031828	42051827	-
4	54050000	54070000	20	1	185	0.11	-5.06	*PDE5A*	54989025	55009036	*PDE5*
4	54640000	54660000	20	1	101	0.16	-4.09	-	55578941	55598939	-
4	58500000	58530000	30	2	198	0.12 ± 0.015	-4.87 ± 0.31	-	59272232	59301913	-
4	65280000	65300000	20	1	220	0.12	-5.02	*LNX1*	66083650	66103650	*LNX1*
4	75380000	75410000	30	2	154	0.13 ± 0.021	-4.63 ± 0.427	*LCORL*	76353909	76384013	*LCORL*
4	77110000	77170000	60	5	155	0.14 ± 0.03	-4.55 ± 0.625	-	78098132	78158138	-
4	77410000	77440000	30	2	132	0.16 ± 0.001	-4.13 ± 0.014	-	78407934	78437935	-
4	77600000	77630000	30	2	133	0.12 ± 0.011	-4.87 ± 0.229	*HS3ST1*	78598179	78628179	*HS3ST1*
4	80840000	80900000	60	3	60	0.11 ± 0.035	-5.03 ± 0.73	-	81850065	81909949	-
4	89900000	89930000	30	2	154	0.14 ± 0.004	-4.44 ± 0.073	-	90956121	90986122	-
5	18260000	18300000	40	3	89	0.12 ± 0.049	-4.91 ± 1.017	*CD44*	18968714	19010440	-
5	40060000	40110000	50	4	197	0.08 ± 0.051	-5.72 ± 1.059	*TSHR, GTF2A1*	40828747	40878736	*TSHR, GTF2A1*
5	13300000	13320000	20	1	127	0.15	-4.27	-	13920523	13940523	-

5	16940000	16980000	40	3	117	0.09 ± 0.02	-5.49 ± 0.423	-	17638570	17678611	-
5	39870000	39930000	60	5	72	0.06 ± 0.049	-6.2 ± 1.006	-	40638743	40698744	-
5	41100000	41150000	50	4	94	0.15 ± 0.007	-4.2 ± 0.146	-	41868267	41918264	-
5	41450000	41470000	20	1	99	0.16	-4.07	*FLRT2*	42218217	42238218	*FLRT2*
5	47950000	47970000	20	1	195	0.15	-4.25	*CCDC85C*	48126316	48146316	-
5	51720000	51750000	30	2	101	0.09 ± 0.06	-5.59 ± 1.24	*AHNAK2*	51895684	51919025	-
5	54080000	54120000	40	3	156	0.12 ± 0.028	-4.98 ± 0.577	-	54251928	54291928	-
5	55190000	55230000	40	3	267	0.14 ± 0.014	-4.59 ± 0.285	*C14orf37*	55431566	55471567	*C14orf37*
6	5550000	5590000	40	3	328	0.11 ± 0.028	-5.12 ± 0.581	-	6115363	6155369	-
6	5610000	5640000	30	2	279	0.05 ± 0.058	-6.34 ± 1.205	-	6175368	6205371	-
6	6070000	6100000	30	2	314	0.1 ± 0.012	-5.41 ± 0.246	*SIRT1*	6635375	6665263	*SIRT1*
6	6480000	6510000	30	2	180	0.14 ± 0.03	-4.52 ± 0.621	*CTNNA3*	7045263	7075263	*CTNNA3*
6	6860000	6920000	60	5	174	0.11 ± 0.04	-5.07 ± 0.821	-	7431893	7491893	-
6	9640000	9690000	50	4	168	0.14 ± 0.01	-4.6 ± 0.216	-	10134248	10184248	*PRKG1*
6	9700000	9750000	50	4	96	0.08 ± 0.046	-5.66 ± 0.957	*PRKG1*	10194248	10244249	*PRKG1*
6	12910000	12940000	30	2	61	0.15 ± 0.008	-4.28 ± 0.17	*KCNMA1*	13345131	13374904	*KCNMA1*
6	15480000	15530000	50	4	129	0.1 ± 0.047	-5.38 ± 0.964	*CHCHD1, FUT11, SEC24C*	15913193	15963193	*CHCHD1, SEC24C, FUT11*
6	15550000	15590000	40	3	111	0.13 ± 0.02	-4.65 ± 0.42	*SYNPO2L, MYOZ1*	15983193	16023120	*MYOZ1, SYNPO2L*
6	15610000	15640000	30	2	198	0.12 ± 0.003	-4.85 ± 0.068	-	16043120	16073121	-
6	16960000	17020000	60	5	204	0.1 ± 0.033	-5.36 ± 0.685	*HIF1AN, RP11-411B6.1, SEC31B, WNT8B*	17404713	17464713	*HIF1AN, WNT8B*
6	17860000	17890000	30	2	182	0.15 ± 0.003	-4.25 ± 0.064	*ARHGAP22*	18316795	18346799	-
7	290000	320000	30	2	126	0.13 ± 0.021	-4.68 ± 0.431	*OSGEPL1, ASNSD1*	312479	342479	*ASNSD1, OSGEPL1*
7	430000	450000	20	1	58	0.14	-4.5	*COL5A2*	452550	472550	*COL5A2*

7	490000	510000	20	1	65	0.08	-5.78	-	512550	532549	*COL5A2*
7	6090000	6110000	20	1	74	0.16	-4.03	-	6061013	6081023	-
7	6370000	6420000	50	4	102	0.12 ± 0.006	-4.88 ± 0.125	*HDAC4*	6341033	6402910	*HDAC4*
7	8070000	8090000	20	1	74	0.12	-4.87	-	8578942	8598945	-
7	11130000	11150000	20	1	142	0.16	-4.09	-	11682904	11702903	-
7	11890000	11920000	30	2	183	0.12 ± 0.013	-4.84 ± 0.268	*FASTKD2, MDH1B, DYTN*	12442251	12472251	*MDH1B, DYTN, FASTKD2*
7	21060000	21100000	40	3	104	0.07 ± 0.081	-5.94 ± 1.68	-	21673840	21713840	-
7	21240000	21260000	20	1	102	0.11	-5.2	*RBMS1*	21853770	21873769	*RBMS1*
7	24870000	24890000	20	1	157	0.14	-4.56	*PTPN4*	25518634	25538636	-
7	27580000	27600000	20	1	125	0.13	-4.8	*SNX4*	28228754	28248755	*SNX4*
7	36160000	36190000	30	2	52	0.12 ± 0.013	-4.95 ± 0.275	*BAZ2B, Mar-07*	36850252	36880269	*BAZ2B, Mar-07*
7	36200000	36230000	30	2	78	0.1 ± 0.013	-5.23 ± 0.262	*NAA20, Mar-07*	36890268	36924725	*NAA20, Mar-07*
8	1	30000	30	2	80	0.13 ± 0.007	-4.71 ± 0.155	-	4572	34532	-
8	200000	230000	30	2	60	0.01 ± 0.004	-7.27 ± 0.087	-	204536	234537	-
8	420000	630000	210	19	113	0.13 ± 0.013	-4.78 ± 0.272	-	424781	634785	-
8	7240000	7270000	30	2	174	0.16 ± 0.01	-4.16 ± 0.208	*RABGAP1L*	7283331	7313331	*RABGAP1L*
8	8730000	8920000	190	18	271	0.11 ± 0.036	-5.2 ± 0.743	-	8806310	9002909	-
8	9020000	9130000	110	10	129	0.15 ± 0.014	-4.33 ± 0.295	-	9108796	9221862	-
8	9330000	9350000	20	1	103	0.15	-4.28	-	9421929	9441929	-
8	9420000	9450000	30	2	143	0.13 ± 0.012	-4.73 ± 0.25	-	9511843	9541920	-
8	10880000	10900000	20	1	81	0.13	-4.75	*TRMT13, SASS6, HIAT1*	11918889	11938889	*TRMT13, SASS6*
8	16040000	16120000	80	7	115	0.13 ± 0.024	-4.66 ± 0.486	-	17163256	17243252	-
8	17920000	17950000	30	2	140	0.15 ± 0.008	-4.35 ± 0.16	*ZZZ3*	19077114	19107110	*ZZZ3*
8	27370000	27390000	20	1	214	0.1	-5.32	-	28566238	28586239	-
8	27810000	27850000	40	3	130	0.14 ± 0.002	-4.51 ± 0.048	*LRRC7*	29006913	29046914	-

9	9000000	9030000	30	2	100	0.13 ± 0.003	-4.63 ± 0.054	*SLC19A3, CCL20*	9519317	9549318	*CCL20*
9	9190000	9220000	30	2	124	0.11 ± 0.045	-5.1 ± 0.933	-	9709261	9739260	-
9	9810000	9910000	100	9	128	0.07 ± 0.042	-5.9 ± 0.874	*TFDP2, GK5, XRN1*	10329299	10443015	*ATP1B3, GK5, XRN1, TFDP2*
9	16860000	16900000	40	3	133	0.14 ± 0.019	-4.46 ± 0.392	*USP13*	17435395	17475021	*USP13*
9	19760000	19790000	30	2	90	0.09 ± 0.001	-5.63 ± 0.016	-	20374004	20404004	-
9	22840000	22860000	20	1	109	0.15	-4.4	*MBNL1*	23474540	23494540	*MBNL1*
10	3900000	3920000	20	1	192	0.16	-4.01	*TLN2*	4338656	4358590	-
10	17980000	18010000	30	2	206	0.14 ± 0.002	-4.41 ± 0.036	*MAP2K1, SNAPC5, RPL4, ZWILCH*	18508838	18538838	*MAP2K1, RPL4, SNAPC5, SNORD16, SNORD18*
11	1800000	1820000	20	1	227	0.13	-4.77	*GLG1*	1867724	1887724	*GLG1*
11	3310000	3340000	30	2	86	0.16 ± 0.008	-4.14 ± 0.165	-	3932540	3962538	-
11	3450000	3500000	50	4	68	0.15 ± 0.019	-4.34 ± 0.385	-	4072227	4122228	-
11	5270000	5290000	20	1	108	0.15	-4.24	-	5898784	5918850	-
11	9180000	9200000	20	1	138	0.16	-4.11	-	9824647	9853201	-
11	12460000	12550000	90	7	65	0.13 ± 0.028	-4.72 ± 0.589	-	13172682	13262615	-
11	12570000	12610000	40	3	72	0.14 ± 0.018	-4.49 ± 0.37	-	13282599	13322604	-
11	14210000	14230000	20	1	84	0.14	-4.54	-	14951445	14971444	-
14	3580000	3610000	30	2	213	0.16 ± 0.001	-4.16 ± 0.024	*SDK1*	3588840	3618842	*SDK1*
14	10290000	10310000	40	2	101	0.13 ± 0.001	-4.72 ± 0.034	-	10359543	10379542	-
14	10560000	10580000	80	6	132	0.16	-4.03	*CACNA1G, MIR163*	10628574	10648589	-
14	10760000	10790000	30	2	92	0.13 ± 0.029	-4.69 ± 0.592	-	10828623	10858622	-
15	11550000	11570000	20	1	86	0.13	-4.75	-	11620492	11640216	*MED13L*

18	10500000	10540000	40	3	126	0.09 ± 0.074	-5.64 ± 1.538	-	10316615	10356615	*CACNA1G, gga-mir-1637*
23	5460000	5490000	30	2	51	0.11 ± 0.004	-5.14 ± 0.075	*HPCAL4, TRIT1, MYCL*	5521860	5551860	*MYCL, HPCAL4, TRIT1*
