# Supplementary material for: Whole-Genome Resequencing of Red Junglefowl and Indigenous Village Chicken Reveal New Insights on the Genome Dynamics of the Species
Source: Front Genet. 2018 Jul 20;9:264. doi: 10.3389/fgene.2018.00264 (PMC6062655; doi:10.3389/fgene.2018.00264)
Supplement: Supplementary file 7 [file Table_7.PDF]

**Table S7 |** Candidate selective sweep regions detected in Sri Lankan domestic chicken population using $H_p$. The **Ggal** is the reference genome annotation for the *Galgal* version 4 or 5, **Ln** is length in kilobase, **nWd** is the number of windows analysed for the sweep region

| **Chr** | **Ggal4_start** | **Ggal4_stop** | **Ln** | **nWd** | **Total SNP** | **mean ($H_p$)** | **mean Z($H_p$)** | **Ggal4_gene** | **Ggal5_start** | **Ggal5_stop** | **Ggal5_gene** |
|---|---|---|---|---|---|---|---|---|---|---|---|
| 1 | 8520000 | 8540000 | 20 | 1 | 274 | 0.16 | -4.03 | *SEMA3A* | 8665357 | 8685324 | - |
| 1 | 18110000 | 18130000 | 20 | 1 | 145 | 0.16 | -4.07 | - | 18311759 | 18331760 | - |
| 1 | 25290000 | 25350000 | 60 | 5 | 216 | 0.14 ± 0.01 | -4.51 ± 0.262 | *TFEC* | 25351496 | 25412595 | - |
| 1 | 25400000 | 25430000 | 30 | 2 | 243 | 0.15 | -4.35 ± 0.008 | - | 25462594 | 25492468 | - |
| 1 | 25450000 | 25470000 | 20 | 1 | 227 | 0.15 | -4.28 | - | 25512469 | 25532471 | - |
| 1 | 25580000 | 25600000 | 20 | 1 | 321 | 0.14 | -4.53 | - | 25642536 | 25662537 | - |
| 1 | 26450000 | 26470000 | 20 | 1 | 270 | 0.16 | -4.04 | - | 26512591 | 26532597 | - |
| 1 | 27840000 | 27870000 | 30 | 2 | 319 | 0.14 ± 0.005 | -4.55 ± 0.131 | - | 27917075 | 27947075 | - |
| 1 | 32430000 | 32450000 | 20 | 1 | 186 | 0.12 | -4.95 | - | 32471039 | 32491038 | - |
| 1 | 42950000 | 42980000 | 30 | 2 | 146 | 0.14 ± 0.02 | -4.55 ± 0.518 | - | 43067034 | 43097039 | - |
| 1 | 43060000 | 43110000 | 50 | 4 | 84 | 0.09 ± 0.025 | -5.96 ± 0.64 | - | 43176831 | 43226829 | - |
| 1 | 43840000 | 43860000 | 20 | 1 | 103 | 0.15 | -4.32 | *KERA* | 43966513 | 43986513 | *KERA* |
| 1 | 75840000 | 75880000 | 40 | 3 | 108 | 0.11 ± 0.036 | -5.34 ± 0.932 | *OVSTL, gga-mir-6606* | 76415999 | 76450241 | *gga-mir-6606* |
| 1 | 79430000 | 79450000 | 20 | 1 | 242 | 0.14 | -4.43 | - | 79990564 | 80010564 | - |
| 1 | 92710000 | 92730000 | 20 | 1 | 180 | 0.15 | -4.19 | *CADM2* | 93354612 | 93374612 | *CADM2* |
| 1 | 103010000 | 103040000 | 30 | 2 | 376 | 0.15 ± 0.003 | -4.24 ± 0.069 | - | 103682296 | 103712296 | - |
| 1 | 110720000 | 110750000 | 30 | 2 | 161 | 0.13 ± 0.027 | -4.83 ± 0.687 | - | 111379827 | 111401672 | - |
| 1 | 118020000 | 118040000 | 20 | 1 | 292 | 0.13 | -4.8 | - | 118579504 | 118599504 | - |
| 1 | 140850000 | 140870000 | 20 | 1 | 119 | 0.16 | -4.05 | - | 141580644 | 141599898 | - |
| 1 | 146980000 | 147000000 | 20 | 1 | 263 | 0.15 | -4.23 | *GPC5* | 147749465 | 147769463 | *GPC5* |
| 1 | 147930000 | 147950000 | 20 | 1 | 99 | 0.16 | -4.05 | - | 148698337 | 148718340 | - |
| 1 | 149380000 | 149410000 | 30 | 2 | 96 | 0.15 ± 0.006 | -4.39 ± 0.148 | - | 150154135 | 150184552 | - |
| 1 | 158080000 | 158100000 | 20 | 1 | 85 | 0.15 | -4.17 | - | 158909666 | 158929683 | - |
| 1 | 189930000 | 189990000 | 60 | 5 | 181 | 0.15 ± 0.016 | -4.32 ± 0.415 | - | 190796652 | 190856658 | - |
| 1 | 190070000 | 190110000 | 40 | 3 | 199 | 0.1 ± 0.014 | -5.51 ± 0.364 | - | 190937229 | 190977200 | - |
| 1 | 190200000 | 190240000 | 40 | 3 | 202 | 0.14 ± 0.018 | -4.56 ± 0.463 | - | 191067211 | 191107508 | - |

| | | | | | | | | | | | |
|---|---|---|---|---|---|---|---|---|---|---|---|
| 2 | 2640000 | 2660000 | 20 | 1 | 246 | 0.14 | -4.45 | - | 2678814 | 2698812 | - |
| 2 | 11890000 | 11910000 | 20 | 1 | 116 | 0.14 | -4.52 | - | 11964104 | 11984107 | - |
| 2 | 24350000 | 24380000 | 30 | 2 | 355 | 0.15 ± 0.007 | -4.27 ± 0.188 | - | 24249156 | 24279167 | - |
| 2 | 27990000 | 28010000 | 20 | 1 | 109 | 0.14 | -4.53 | *AGMO* | 27911972 | 27931970 | *AGMO* |
| 2 | 28260000 | 28290000 | 30 | 2 | 102 | 0.11 ± 0.028 | -5.23 ± 0.722 | *5S_rRNA* | 28181919 | 28211919 | *5S_rRNA* |
| 2 | 36010000 | 36040000 | 30 | 2 | 240 | 0.14 ± 0.014 | -4.48 ± 0.365 | *KCNH8* | 35572340 | 35602326 | *KCNH8* |
| 2 | 36170000 | 36190000 | 20 | 1 | 281 | 0.13 | -4.73 | - | 35732316 | 35752317 | *KCNH8* |
| 2 | 36200000 | 36220000 | 20 | 1 | 273 | 0.16 | -4.06 | - | 35762317 | 35782317 | - |
| 2 | 52310000 | 52330000 | 20 | 1 | 83 | 0.15 | -4.28 | *VOPP1* | 51887766 | 51905922 | *VOPP1* |
| 2 | 52410000 | 52430000 | 20 | 1 | 67 | 0.14 | -4.66 | *EGFR* | 51985932 | 52005932 | *EGFR* |
| 2 | 52500000 | 52540000 | 40 | 3 | 82 | 0.15 ± 0.01 | -4.18 ± 0.252 | *EGFR* | 52075837 | 52115805 | *EGFR* |
| 2 | 52610000 | 52630000 | 20 | 1 | 116 | 0.15 | -4.28 | - | 52186033 | 52206034 | - |
| 2 | 52650000 | 52670000 | 20 | 1 | 70 | 0.16 | -4.08 | - | 52226035 | 52245967 | - |
| 2 | 52690000 | 52720000 | 30 | 2 | 103 | 0.15 ± 0.009 | -4.39 ± 0.245 | *SEC61G* | 52265804 | 52295807 | *SEC61G* |
| 2 | 52870000 | 52890000 | 20 | 1 | 76 | 0.16 | -4.03 | *TPK1* | 52982606 | 53002606 | *TPK1* |
| 2 | 52980000 | 53050000 | 70 | 6 | 148 | 0.15 ± 0.01 | -4.38 ± 0.252 | *TPK1* | 53092604 | 53162921 | *TPK1* |
| 2 | 53090000 | 53180000 | 90 | 8 | 116 | 0.16 ± 0.012 | -4.15 ± 0.323 | *TPK1* | 53212192 | 53292136 | - |
| 2 | 54720000 | 54740000 | 20 | 1 | 244 | 0.16 | -4.16 | - | 54807792 | 54827790 | - |
| 2 | 78310000 | 78340000 | 30 | 2 | 168 | 0.12 ± 0.01 | -5.2 ± 0.259 | - | 78554635 | 78584572 | - |
| 2 | 81900000 | 81920000 | 20 | 1 | 143 | 0.15 | -4.23 | - | 82140679 | 82160679 | - |
| 2 | 81950000 | 82240000 | 290 | 27 | 125 | 0.14 ± 0.018 | -4.65 ± 0.454 | - | 82190953 | 82481139 | - |
| 2 | 86710000 | 86730000 | 20 | 1 | 113 | 0.15 | -4.33 | - | 86985708 | 87005707 | - |
| 2 | 86790000 | 86820000 | 30 | 2 | 122 | 0.15 ± 0.006 | -4.29 ± 0.166 | - | 87066280 | 87096280 | - |
| 2 | 87440000 | 87460000 | 20 | 1 | 86 | 0.16 | -4.08 | - | 87730072 | 87750073 | - |
| 2 | 91940000 | 91960000 | 20 | 1 | 306 | 0.15 | -4.2 | *CYB5A* | 92245735 | 92265735 | *CYB5A* |
| 2 | 95760000 | 95780000 | 20 | 1 | 108 | 0.16 | -4.01 | - | 96102435 | 96122454 | - |
| 2 | 108940000 | 108980000 | 40 | 3 | 199 | 0.13 ± 0.01 | -4.93 ± 0.255 | - | 109577665 | 109617666 | - |
| 2 | 118490000 | 118520000 | 30 | 2 | 126 | 0.15 ± 0.019 | -4.35 ± 0.482 | - | 119213580 | 119243580 | - |
| 2 | 140970000 | 141010000 | 40 | 3 | 199 | 0.15 ± 0.01 | -4.32 ± 0.268 | *HHLA1, KCNQ3* | 141501204 | 141541206 | *HHLA1, KCNQ3* |
| 2 | 146620000 | 146650000 | 30 | 2 | 175 | 0.12 ± 0.002 | -5.12 ± 0.061 | - | 147154279 | 147184251 | - |

| | | | | | | | | | | | |
|---|---|---|---|---|---|---|---|---|---|---|---|
| 2 | 146720000 | 146740000 | 20 | 1 | 199 | 0.13 | -4.92 | - | 147254792 | 147274793 | - |
| 3 | 1120000 | 1210000 | 90 | 8 | 111 | 0.14 ± 0.017 | -4.59 ± 0.445 | - | 1146438 | 1236442 | - |
| 3 | 23050000 | 23080000 | 30 | 2 | 166 | 0.13 ± 0.017 | -4.87 ± 0.445 | *EML4* | 23765628 | 23795628 | *EML4* |
| 3 | 69020000 | 69060000 | 40 | 3 | 159 | 0.15 ± 0.008 | -4.23 ± 0.2 | - | 69835999 | 69875998 | - |
| 3 | 69250000 | 69270000 | 20 | 1 | 155 | 0.16 | -4.1 | - | 70066048 | 70086052 | - |
| 3 | 69370000 | 69390000 | 20 | 1 | 161 | 0.16 | -4 | - | 70186196 | 70206297 | - |
| 3 | 77550000 | 77570000 | 20 | 1 | 257 | 0.15 | -4.17 | - | 78420044 | 78439926 | - |
| 3 | 81600000 | 81630000 | 30 | 2 | 392 | 0.14 ± 0.007 | -4.49 ± 0.178 | *RIMS1* | 82474657 | 82504658 | *RIMS1* |
| 3 | 99070000 | 99100000 | 30 | 2 | 202 | 0.15 ± 0.003 | -4.29 ± 0.09 | - | 99956352 | 99986352 | - |
| 3 | 110160000 | 110290000 | 130 | 12 | 263 | 0.14 ± 0.006 | -4.45 ± 0.162 | *EVA1A* | 111008970 | 111138863 | - |
| 4 | 25240000 | 25260000 | 20 | 1 | 196 | 0.14 | -4.47 | - | 25981856 | 26001851 | - |
| 4 | 26410000 | 26430000 | 20 | 1 | 253 | 0.15 | -4.2 | - | 27150530 | 27174313 | - |
| 4 | 27860000 | 27910000 | 50 | 4 | 186 | 0.16 ± 0.013 | -4.13 ± 0.341 | - | 28621318 | 28671317 | - |
| 4 | 32410000 | 32440000 | 30 | 2 | 250 | 0.13 ± 0.016 | -4.79 ± 0.409 | *LRBA* | 33217876 | 33247878 | *LRBA* |
| 4 | 38590000 | 38640000 | 50 | 4 | 247 | 0.1 ± 0.034 | -5.52 ± 0.874 | *TACR3* | 39419744 | 39469745 | *TACR3* |
| 4 | 40630000 | 40650000 | 20 | 1 | 92 | 0.16 | -4.15 | - | 41472219 | 41492219 | - |
| 4 | 56900000 | 56920000 | 20 | 1 | 224 | 0.14 | -4.59 | - | 57663311 | 57683312 | - |
| 4 | 61190000 | 61230000 | 40 | 3 | 224 | 0.16 ± 0.013 | -4 ± 0.332 | *RP11-215A19.2* | 61984820 | 62024820 | *MTNR1A* |
| 4 | 61750000 | 61770000 | 20 | 1 | 218 | 0.14 | -4.59 | - | 62544568 | 62564571 | - |
| 4 | 72190000 | 72210000 | 20 | 1 | 314 | 0.15 | -4.33 | - | 73067668 | 73087708 | - |
| 4 | 77080000 | 77100000 | 20 | 1 | 263 | 0.16 | -4.14 | *RAB28* | 78068132 | 78088132 | *RAB28* |
| 5 | 3800000 | 3840000 | 40 | 3 | 64 | 0.16 ± 0.003 | -4.05 ± 0.082 | - | 3758345 | 3798346 | - |
| 5 | 4240000 | 4260000 | 20 | 1 | 84 | 0.14 | -4.51 | - | 4201902 | 4221909 | - |
| 5 | 21670000 | 21890000 | 220 | 19 | 71 | 0.11 ± 0.033 | -5.4 ± 0.866 | - | 22371859 | 22591888 | - |
| 5 | 21980000 | 22010000 | 30 | 2 | 202 | 0.13 ± 0.009 | -4.69 ± 0.237 | - | 22682551 | 22711752 | - |
| 5 | 30360000 | 30400000 | 40 | 3 | 105 | 0.15 ± 0.009 | -4.34 ± 0.245 | - | 31096727 | 31136725 | - |
| 5 | 39870000 | 39890000 | 20 | 1 | 164 | 0.15 | -4.33 | - | 40638743 | 40658743 | - |
| 5 | 40060000 | 40110000 | 50 | 4 | 187 | 0.07 ± 0.063 | -6.32 ± 1.634 | *TSHR, GTF2A1* | 40828747 | 40878736 | *TSHR, GTF2A1* |
| 5 | 40880000 | 40910000 | 30 | 2 | 190 | 0.13 ± 0.043 | -4.82 ± 1.111 | - | 41648280 | 41678280 | - |

| | | | | | | | | | | | |
|---|---|---|---|---|---|---|---|---|---|---|---|
| 5 | 41100000 | 41160000 | 60 | 5 | 115 | 0.13 ± 0.033 | -4.89 ± 0.862 | - | 41868267 | 41928265 | - |
| 5 | 46470000 | 46490000 | 20 | 1 | 110 | 0.16 | -4.12 | - | 47284217 | 47304374 | - |
| 6 | 8780000 | 8800000 | 20 | 1 | 96 | 0.15 | -4.26 | *BKJ* | 9283122 | 9294493 | *BKJ* |
| 6 | 13310000 | 13330000 | 20 | 1 | 67 | 0.15 | -4.28 | *KCNMA1* | 13744753 | 13764764 | *KCNMA1* |
| 6 | 17860000 | 17950000 | 90 | 8 | 214 | 0.15 ± 0.008 | -4.18 ± 0.197 | *ARHGAP22* | 18316795 | 18406698 | - |
| 6 | 18020000 | 18040000 | 20 | 1 | 257 | 0.16 | -4.14 | *WDFY4* | 18476698 | 18496698 | - |
| 7 | 7890000 | 7930000 | 40 | 3 | 121 | 0.14 ± 0.015 | -4.55 ± 0.388 | - | 8399045 | 8439044 | - |
| 7 | 8070000 | 8100000 | 30 | 2 | 109 | 0.14 ± 0.024 | -4.6 ± 0.629 | - | 8578942 | 8608945 | - |
| 7 | 14810000 | 14830000 | 20 | 1 | 58 | 0.16 | -4.17 | *TTN* | 15373407 | 15393407 | - |
| 7 | 36140000 | 36210000 | 70 | 6 | 72 | 0.13 ± 0.018 | -4.86 ± 0.462 | *BAZ2B, MARCH7, NAA20* | 36830252 | 36900268 | *NAA20, BAZ2B, Mar-07* |
| 8 | 10630000 | 10650000 | 20 | 1 | 93 | 0.15 | -4.3 | - | 11668855 | 11688853 | - |
| 8 | 11330000 | 11360000 | 30 | 2 | 136 | 0.14 ± 0.019 | -4.54 ± 0.484 | *SNX7* | 12368824 | 12398824 | *SNX7* |
| 8 | 11420000 | 11450000 | 30 | 2 | 137 | 0.16 ± 0.004 | -4.1 ± 0.102 | - | 12458826 | 12496487 | - |
| 8 | 12050000 | 12070000 | 20 | 1 | 70 | 0.12 | -5.06 | - | 13103912 | 13123912 | - |
| 8 | 12090000 | 12140000 | 50 | 4 | 167 | 0.15 ± 0.012 | -4.21 ± 0.305 | - | 13143915 | 13193915 | - |
| 8 | 16000000 | 16030000 | 30 | 2 | 274 | 0.16 ± 0.005 | -4.12 ± 0.135 | - | 17123257 | 17153256 | - |
| 8 | 16050000 | 16070000 | 20 | 1 | 208 | 0.15 | -4.34 | - | 17173256 | 17193255 | - |
| 8 | 28540000 | 28580000 | 40 | 2 | 86 | 0.16 ± 0.002 | -4.09 ± 0.016 | *TYW3* | 29736834 | 29776834 | *TYW3* |
| 9 | 10960000 | 10980000 | 20 | 1 | 138 | 0.13 | -4.84 | *PLOD2* | 11499478 | 11519475 | *PLOD2* |
| 10 | 5350000 | 5390000 | 40 | 3 | 187 | 0.14 ± 0.016 | -4.47 ± 0.402 | *APBA2* | 5788488 | 5828489 | *APBA2* |
| 11 | 10400000 | 10420000 | 20 | 1 | 255 | 0.15 | -4.2 | *KIAA0355* | 11054882 | 11074888 | *KIAA0355* |
| 11 | 12350000 | 12390000 | 40 | 3 | 199 | 0.13 ± 0.032 | -4.82 ± 0.83 | *CDH8* | 13046168 | 13102512 | *CDH8* |
| 11 | 12810000 | 12830000 | 20 | 1 | 185 | 0.15 | -4.26 | - | 13522597 | 13542604 | - |
| 11 | 14890000 | 14910000 | 20 | 1 | 222 | 0.15 | -4.32 | - | 15644830 | 15664829 | - |
| 11 | 18720000 | 18750000 | 30 | 2 | 95 | 0.14 ± 0.008 | -4.61 ± 0.215 | - | 19516200 | 19546198 | - |
| 12 | 16600000 | 16620000 | 20 | 1 | 138 | 0.15 | -4.38 | *PDZRN3* | 16647821 | 16667822 | *PDZRN3* |
| 18 | 10910000 | 10940000 | 30 | 2 | 52 | 0.14 ± 0.022 | -4.59 ± 0.565 | *SLC16A5, ARMC7, HN1* | 10732550 | 10762550 | *SLC16A5, ARMC7, JPT1* |
| 18 | 11080000 | 11100000 | 20 | 1 | 51 | 0.15 | -4.35 | *TMEM94* | 10902378 | 10922379 | *TMEM94* |

| | | | | | | | | | | | |
|---|---|---|---|---|---|---|---|---|---|---|---|
| 18 | 11120000 | 11140000 | 20 | 1 | 58 | 0.16 | -4.1 | *CASKIN2, MCHR1* | 10942379 | 10962384 | *MCHR1, CASKIN2* |
| 22 | 230000 | 300000 | 70 | 6 | 133 | 0.13 ± 0.016 | -4.9 ± 0.403 | *ANTXR1, GKN2, BMP10, ARHGAP25* | 238113 | 308051 | *GKN2, ARHGAP25, ANTXR1, BMP10* |
| 22 | 560000 | 580000 | 20 | 1 | 56 | 0.15 | -4.24 | *PPP2R2A* | 568106 | 588044 | *PPP2R2A* |
| 22 | 630000 | 650000 | 20 | 1 | 68 | 0.16 | -4.09 | *EBF2* | 638042 | 658043 | *EBF2* |
| 22 | 1040000 | 1070000 | 30 | 2 | 87 | 0.13 ± 0.009 | -4.75 ± 0.227 | *ADAM28* | 1048083 | 1078084 | - |
| 22 | 1110000 | 1140000 | 30 | 2 | 58 | 0.15 ± 0.006 | -4.2 ± 0.158 | - | 1118082 | 1148094 | - |
| 22 | 1220000 | 1250000 | 30 | 2 | 135 | 0.15 ± 0.009 | -4.19 ± 0.246 | *LOXL2, R3HCC1* | 1228020 | 1258019 | *LOXL2* |
| 22 | 1590000 | 1610000 | 20 | 1 | 113 | 0.16 | -4.07 | *SLC18A1, MAK16, TTI2, RNF122* | 1616510 | 1636436 | *SLC18A1, DUSP26, RNF122, TTI2, MAK16* |
| 23 | 5420000 | 5490000 | 70 | 6 | 88 | 0.14 ± 0.028 | -4.66 ± 0.723 | *PABPC4, HEYL, NT5C1A, HPCAL4, TRIT1, MYCL* | 5481959 | 5551860 | *HPCAL4, TRIT1, MYCL, SNORA55,NT5C1A, HEYL* |
| 26 | 160000 | 190000 | 30 | 2 | 58 | 0.15 ± 0.012 | -4.33 ± 0.306 | *MAPK13, BRPF3* | 164475 | 194481 | *MAPK13* |
| 26 | 5230000 | 5250000 | 20 | 1 | 62 | 0.15 | -4.23 | *ATP2B4* | 5195938 | 5215915 | *ATP2B4* |
| 26 | 5290000 | 5310000 | 20 | 1 | 62 | 0.15 | -4.4 | - | 5255921 | 5275925 | - |
| 27 | 4970000 | 5040000 | 70 | 4 | 58 | 0.14 ± 0.01 | -4.67 ± 0.262 | *MLX, PSMC3IP, FAM134C, TUBG1, PLEKHH3, CNTNAP1,* | 5393751 | 5462838 | *EZH1, CCR10, WNK4, Mlx, VPS25, RAMP2, CNTNAP1, RETREG3, PSMC3IP* |

| | | | | | | | | | | | |
|---|---|---|---|---|---|---|---|---|---|---|---|
| | | | | | | | | *EZH1, RAMP2, WNK4, CCR10* | | | |
| 27 | 5070000 | 5110000 | 40 | 3 | 66 | 0.14 ± 0.015 | -4.51 ± 0.379 | *AARSD1, RUNDC1, RPL27, IFI35* | 5492836 | 5532835 | *G6PC, IFI35, AARSD1, RUNDC1* |
