# Supplementary material for: Whole-Genome Resequencing of Red Junglefowl and Indigenous Village Chicken Reveal New Insights on the Genome Dynamics of the Species
Source: Front Genet. 2018 Jul 20;9:264. doi: 10.3389/fgene.2018.00264 (PMC6062655; doi:10.3389/fgene.2018.00264)
Supplement: Supplementary file 8 [file Table_8.PDF]

Table S8 | Gene ontology analysis for red junglefowl and domestic chicken populations using DAVID version 6.8[1].

A. Red Junglefowl population

GO term	Description	*P* value	Genes
GO:0050789	Regulation of biological process	0.009225514	*ADCY1, AL158801.1,* ***EBF2****, KCNA5,* ***PPP2R2A****, CCND2, NAB1, NEFM, FURIN,* ***BMP10****, GNRH1,* ***ARHGAP25****,* ***MYCL****, NPY2R, RHOBTB2, PDIA3, ZNF302, BCAR3, NKX2-6, ADAM9, ZFHX4,* ***KCNMA1****, RCAN3, PAFAH1B1*
GO:0051246	Regulation of protein metabolic process	0.015403594	*AL158801.1, CCND2, ADAM9, FURIN,* ***BMP10***
GO:0006810	Transport	0.020966967	*SCN2A, ATP6V1B2, KCNA5, GRIK2, MFSD2A, SLC25A37, NEFM, KCNA1, FURIN, KCTD9, SLC16A5, KCNMA1, SLC26A8, PAFAH1B1*
GO:0015672	Monovalent inorganic cation transport	0.022221846	*ATP6V1B2, KCNA5, KCNA1, KCTD9,* ***KCNMA1***
GO:0022836	Gated channel activity	0.024342944	*KCNA5, GRIK2, KCNA1, KCTD9,* ***KCNMA1***
GO:0008088	Axon cargo transport	0.039408326	*NEFM, PAFAH1B1*
GO:0051592	Response to calcium ion	0.045827198	*ADAM9,* ***KCNMA1***

Note: Genes in bold overlap with other genes selected in domestic chicken populations

B. Ethiopian domestic chicken population

GO term	Description	*P* value	Genes
GO:0019229	Regulation of vasoconstriction	0.01063	*HRH1, AGTR1*

[1] https://david.ncifcrf.gov/

C. Saudi Arabian domestic chicken population

GO term	Description	*P* value	Genes
GO:0008015	Blood circulation	0.004766	*PRKG1, PDE5A, SEMA3A, PDE3A, HDAC4, CTNNA3, CACNA1G, GCLC*
GO:0008016	Regulation of heart contraction	0.007859	*PDE5A, SEMA3A, HDAC4, CTNNA3, CACNA1G*
GO:0090257	Regulation of muscle system process	0.009369	*PRKG1, PDE5A, MTPN, HDAC4, CTNNA3*
GO:0051094	Positive regulation of developmental process	0.010359	*TSHR, PDE5A, RHOB, SIRT1, PROX1, FLRT2, SEMA3A, RIMS1, PDE3A, IMPACT, MAP2K1, TMEM30A, HIF1AN*
GO:0006886	Intracellular protein transport	0.011917	*RHOB, SEC24C, RABGAP1L, MYO6, RIMS1*
GO:0060284	Regulation of cell development	0.016126	*PDE5A, PROX1, FLRT2, SEMA3A, RIMS1, PDE3A, HDAC4, IMPACT, MYCL, MAP2K1, TMEM30A*
GO:0070301	Cellular response to hydrogen peroxide	0.017447	*RHOB, SIRT1, IMPACT*
GO:0008206	Bile acid metabolic process	0.018957	*AKR1D1, SIRT1, PROX1*
GO:0045892	Negative regulation of transcription, DNA-templated	0.019806	*SIRT1, TFDP2, PHF14, FRK, PROX1, DACH1, HDAC4, IMPACT, AICDA, HIF1AN, GCLC*
GO:1902679	Negative regulation of RNA biosynthetic process	0.022875	*SIRT1, TFDP2, PHF14, FRK, PROX1, DACH1, HDAC4, IMPACT, AICDA, HIF1AN, GCLC*
GO:0043502	Regulation of muscle adaptation	0.02443	*PDE5A, MTPN, HDAC4*
GO:0005912	Adherens junction	0.025717	*RHOB, TNS3, ARHGAP22, TLN2, FLRT2, RSU1, CTNNA3, RPL4*
GO:0042391	Regulation of membrane potential	0.026217	*RIMS1, NETO1, KCNMA1, CTNNA3, CACNA1G, GCLC*
GO:2000243	Positive regulation of reproductive process	0.028393	*PDE5A, SEMA3A, PDE3A*
GO:0045595	Regulation of cell differentiation	0.030314	*SIRT1, GLG1, PROX1, SEMA3A, RIMS1, MYCL, MAP2K1, TMEM30A, HIF1AN, PDE5A, FLRT2, HDAC4, PDE3A, IMPACT, COL5A2*
GO:0055117	Regulation of cardiac muscle contraction	0.030465	*PDE5A, HDAC4, CTNNA3*
GO:0044712	Single-organism catabolic process	0.030569	*RP11-240B13.2, PDE5A, GK5, AKR1D1, ABHD5, LPIN1, PDE3A, HDAC4, USP13*
GO:0009117	Nucleotide metabolic process	0.039809	*TSHR, PDE5A, PRPS2, RP11-411B6.6, GRM8, MDH1B, PDE3A, HDAC4*
GO:0019934	Cgmp-mediated signaling	0.040082	*PRKG1, PDE3A*
GO:0034983	Peptidyl-lysine deacetylation	0.040082	*SIRT1, HDAC4*
GO:0019693	Ribose phosphate metabolic process	0.040998	*TSHR, PDE5A, PRPS2, RP11-411B6.6, GRM8, PDE3A, HDAC4*
GO:0005875	Microtubule associated complex	0.045734	*DYNC1I1, SNX4, KIF26B, BORCS5*

GO:0044281	Small molecule metabolic process	0.045786	*GK5, PRPS2, SIRT1, LARS2, PROX1, GRM8, LPIN1, ASNSD1, MDH1B, GCLC, PDE5A, TSHR, AKR1D1, RP11-411B6.6, HDAC4, PDE3A*

D. Sri Lankan domestic chicken population

**GO term**	**Description**	***P* value**	**Genes**
GO:0016500	protein-hormone receptor activity	0.04484	*TSHR, MCHR1*
