# Supplementary material for: Whole-Genome Resequencing of Red Junglefowl and Indigenous Village Chicken Reveal New Insights on the Genome Dynamics of the Species
Source: Front Genet. 2018 Jul 20;9:264. doi: 10.3389/fgene.2018.00264 (PMC6062655; doi:10.3389/fgene.2018.00264)
Supplement: Supplementary file 9 [file Table_9.PDF]

Table S9 | Kyoto Encyclopaedia of Genes and Genomes pathway analysis for red junglefowl and domestic chicken populations using KOBAS version 3.0[1].

A. Red Junglefowl population

**ID**	**Description**	***P* value**	**Genes**
gga04914	Progesterone-mediated oocyte maturation	0.033069783	*RPS6KA1, ADCY1, CDC23*
gga00230	Purine metabolism	0.039356647	*ADCY1, ENTPD4, GUCY1A3, NT5C1A*
gga04270	Vascular smooth muscle contraction	0.049550781	***KCNMA1**, GUCY1A3, ADCY1*

B. Ethiopian domestic chicken population

**ID**	**Description**	***P* value**	**Genes**
gga04080	Neuroactive ligand-receptor interaction	0.004583	*AGTR1, TACR3, HRH1, TSHR*
gga04020	Calcium signaling pathway	0.008034	*AGTR1, TACR3, HRH1*
gga04261	Adrenergic signaling in cardiomyocytes	0.041098	*AGTR1, ATP1B3*

C. Saudi Arabian domestic chicken population

**ID**	**Description**	***P* value**	**Genes**
gga04620	Toll-like receptor signaling pathway	0.038572949	*TLR7, MAP2K1, MAP3K7*
gga04270	Vascular smooth muscle contraction	0.047517517	*KCNMA1, PRKG1, MAP2K1*

D. Sri Lankan domestic chicken population

**ID**	**Description**	***P* value**	**Genes**
gga04261	Adrenergic signaling in cardiomyocytes	0.02710155	*ATP2B4, MAPK13, **PPP2R2A***
gga04020	Calcium signaling pathway	0.049582166	*ATP2B4, TACR3, EGFR*

[1] http://kobas.cbi.pku.edu.cn/
