# Supplementary material for: Whole-Genome Resequencing of Red Junglefowl and Indigenous Village Chicken Reveal New Insights on the Genome Dynamics of the Species
Source: Front Genet. 2018 Jul 20;9:264. doi: 10.3389/fgene.2018.00264 (PMC6062655; doi:10.3389/fgene.2018.00264)
Supplement: Supplementary file 10 [file Data_Sheet_1.PDF]

Supplementary Figures

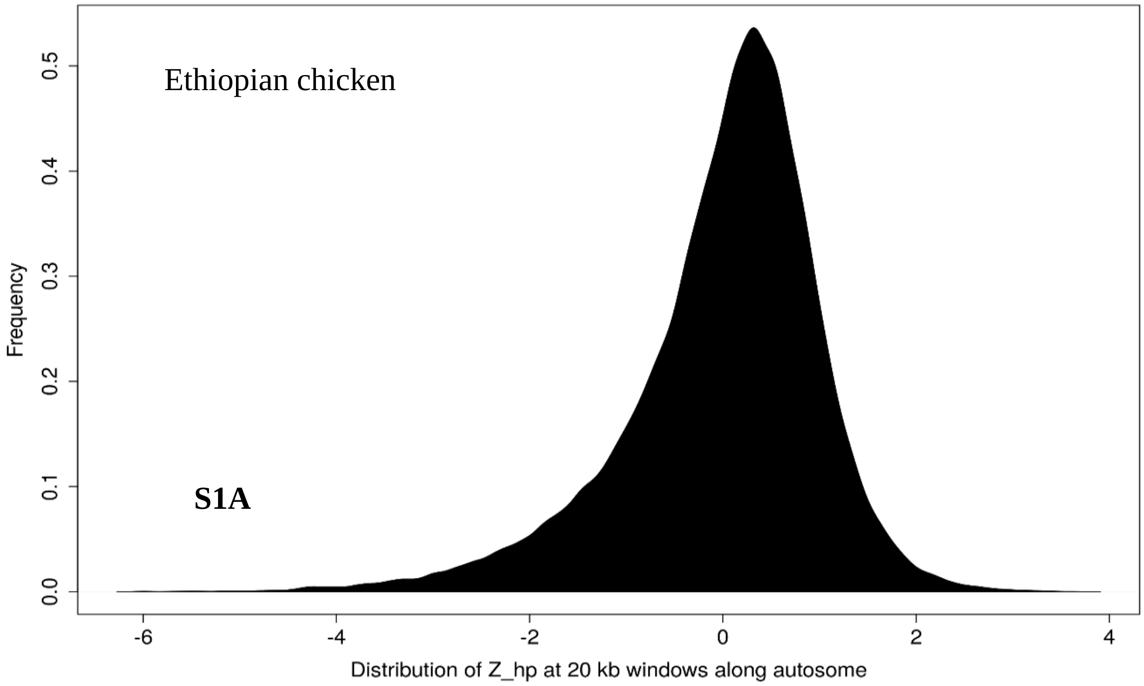

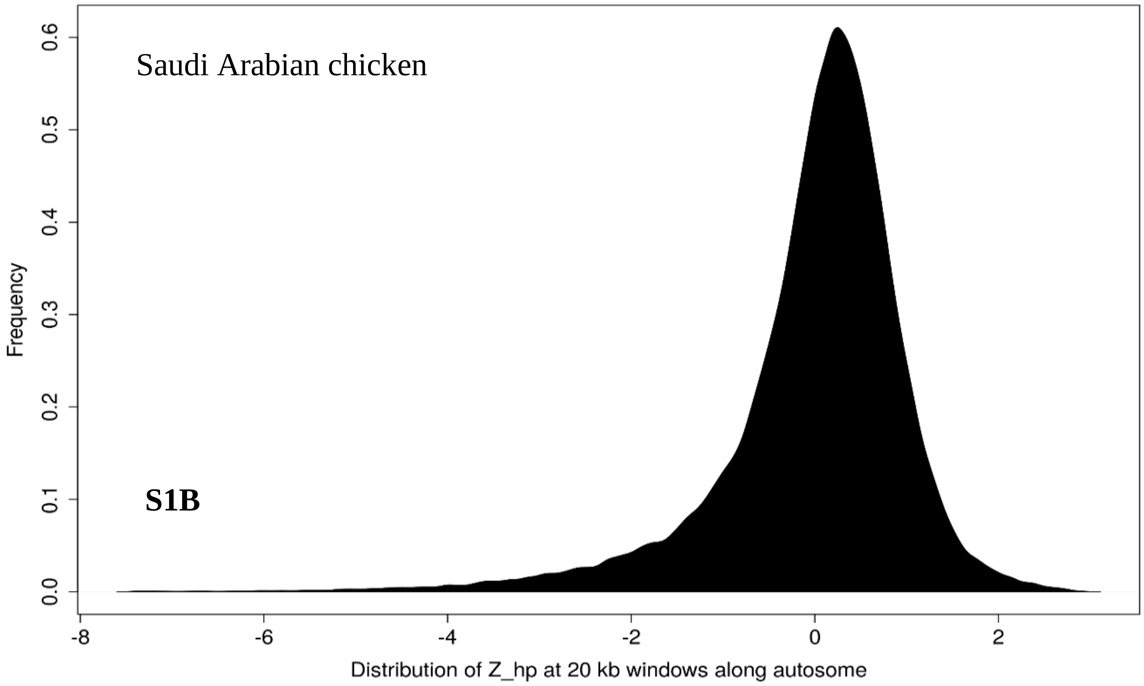

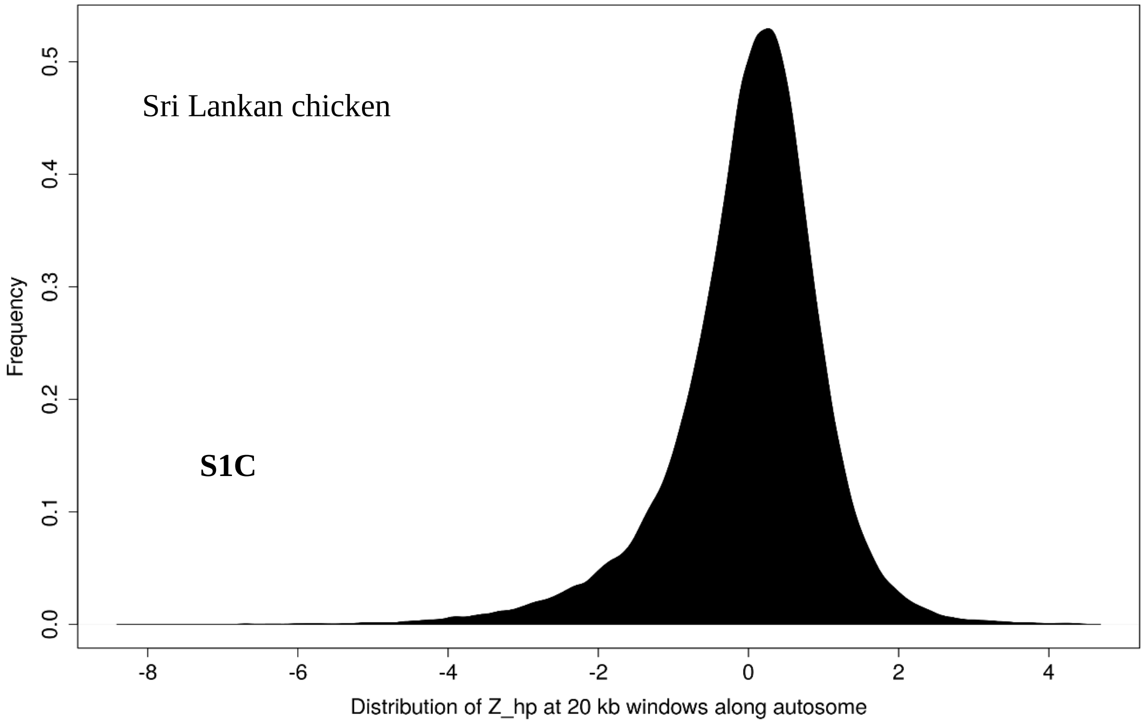

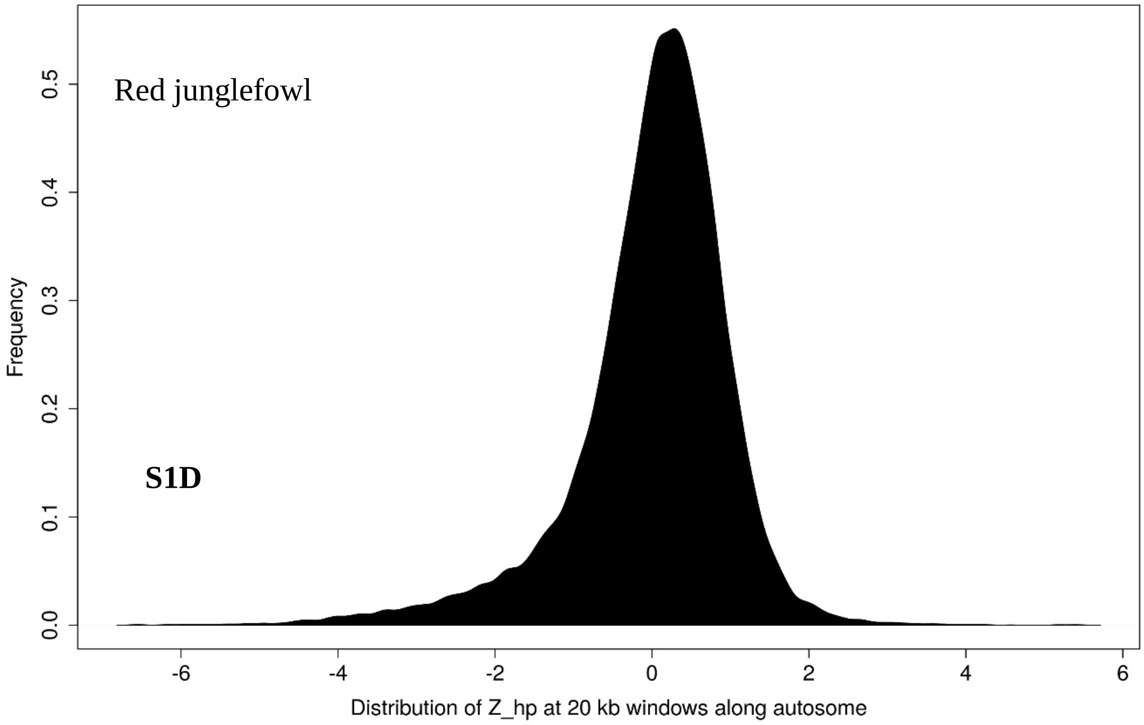

FIGURES S1A - S1D | The distribution of Z-transformed pool heterozygosity Z(H_p) values for the 20 kb windows at 10 kb step size along the autosomes for the studied population

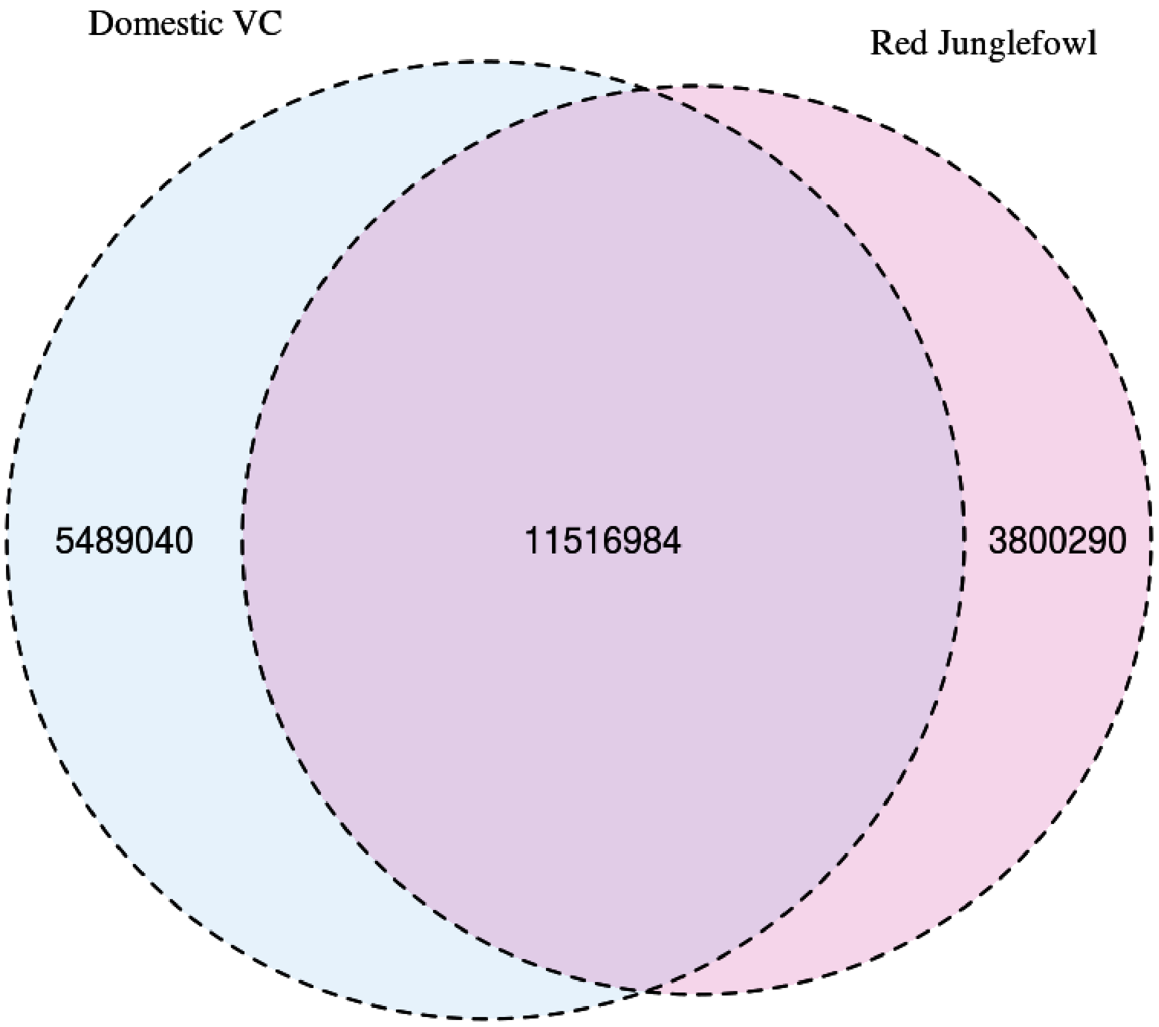

FIGURE S2 | Venn diagram showing the shared and unique SNPs between 27 domestic village chickens (VC) and six red junglefowls.

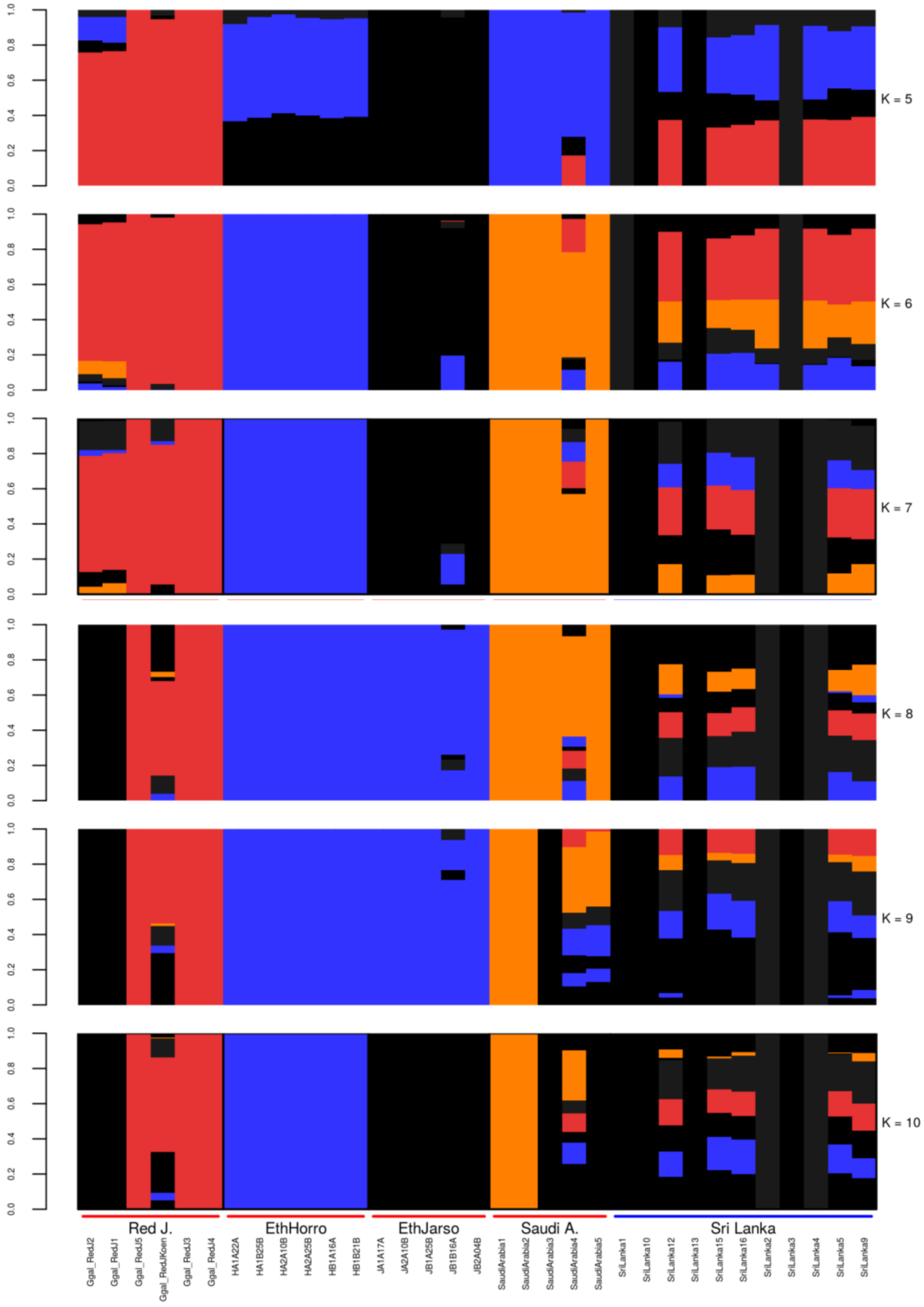

FIGURE S3 | Autosomal admixture plots for $5 \leq K \leq 10$

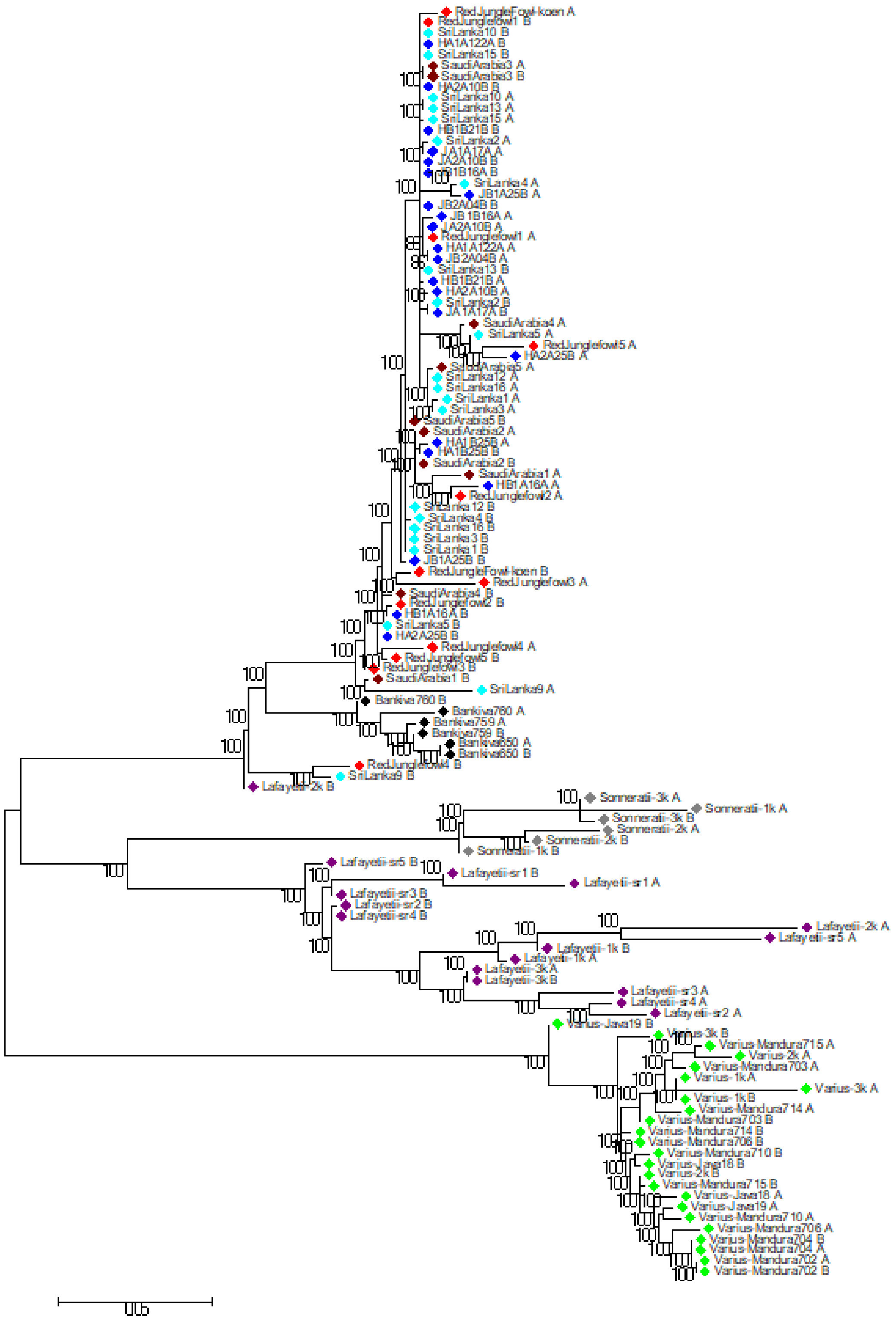

FIGURE S4 | A 20 kb candidate selected region on chromosome 7 (*Galgal* 5.0 position 8578942 - 8598945) common to the four domestic chicken populations and the red junglefowl. ◆: Ethiopian chicken; ◆: Saudi Arabian chicken; ◆: Sri Lankan chicken; ◆: Red junglefowl; ◆: Javan red junglefowl; ◆: Grey junglefowl; ◆: Ceylon junglefowl; ◆: Green junglefowl

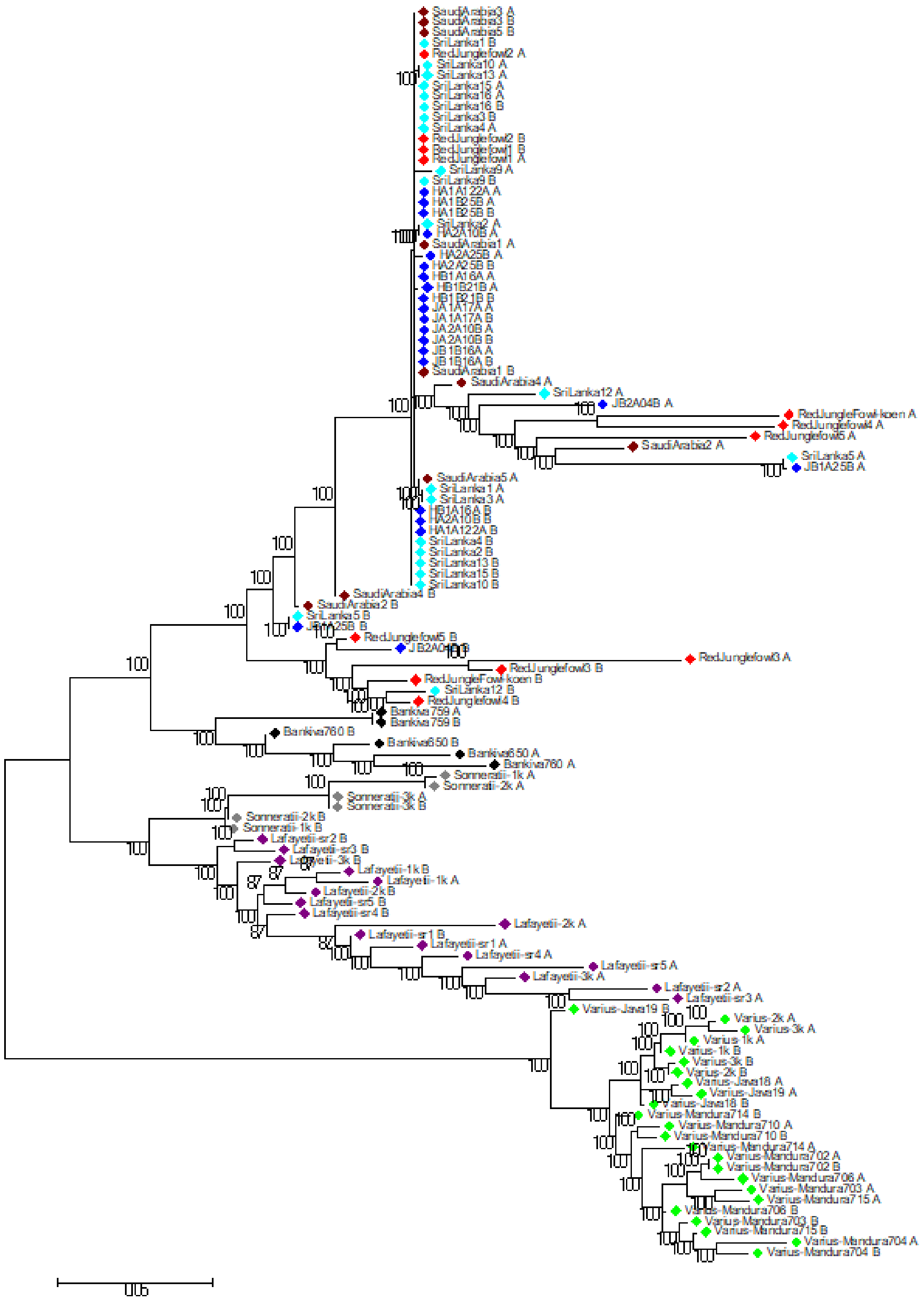

FIGURE S5 | A 20 kb candidate selected region on chromosome 1 (*Galgal* 5.0 position 190947207 - 190967194) shared between the four domestic chicken population. [blue diamond]: Ethiopian chicken; [brown diamond]: Saudi Arabian chicken; [cyan diamond]: Sri Lankan chicken; [red diamond]: Red junglefowl; [black diamond]: Javan red junglefowl; [grey diamond]: Grey junglefowl; [purple diamond]: Ceylon junglefowl; [green diamond]: Green junglefowl.

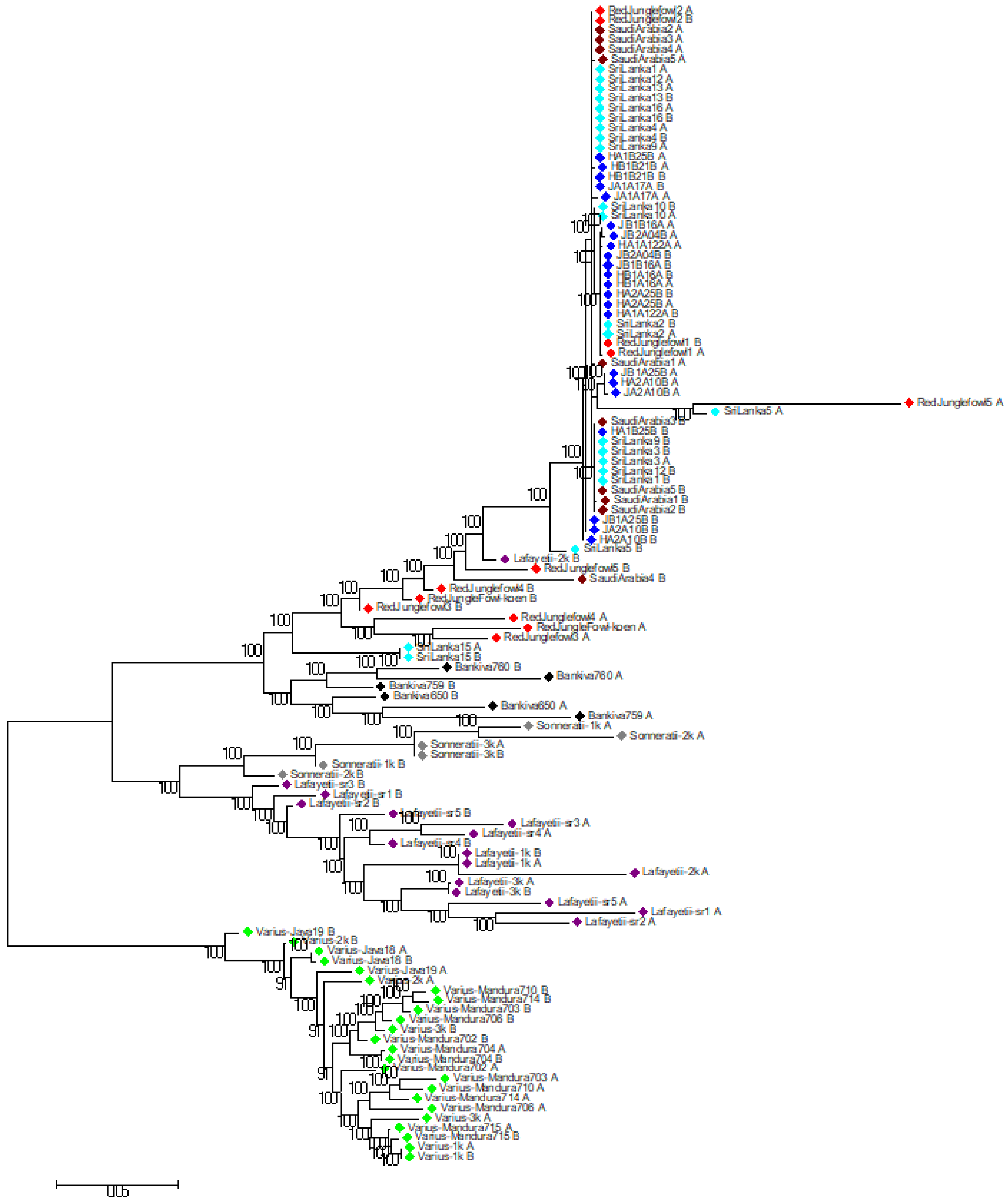

FIGURE S6 | A 20 kb candidate selected region on chromosome 2 (*Galgal* 5.0 position 147254792 - 147274793) shared between the four domestic chicken population. ◆: Ethiopian chicken; ◆: Saudi Arabian chicken; ◆: Sri Lankan chicken; ◆: Red junglefowl; ◆: Javan red junglefowl; ◆: Grey junglefowl; ◆: Ceylon junglefowl; ◆: Green junglefowl

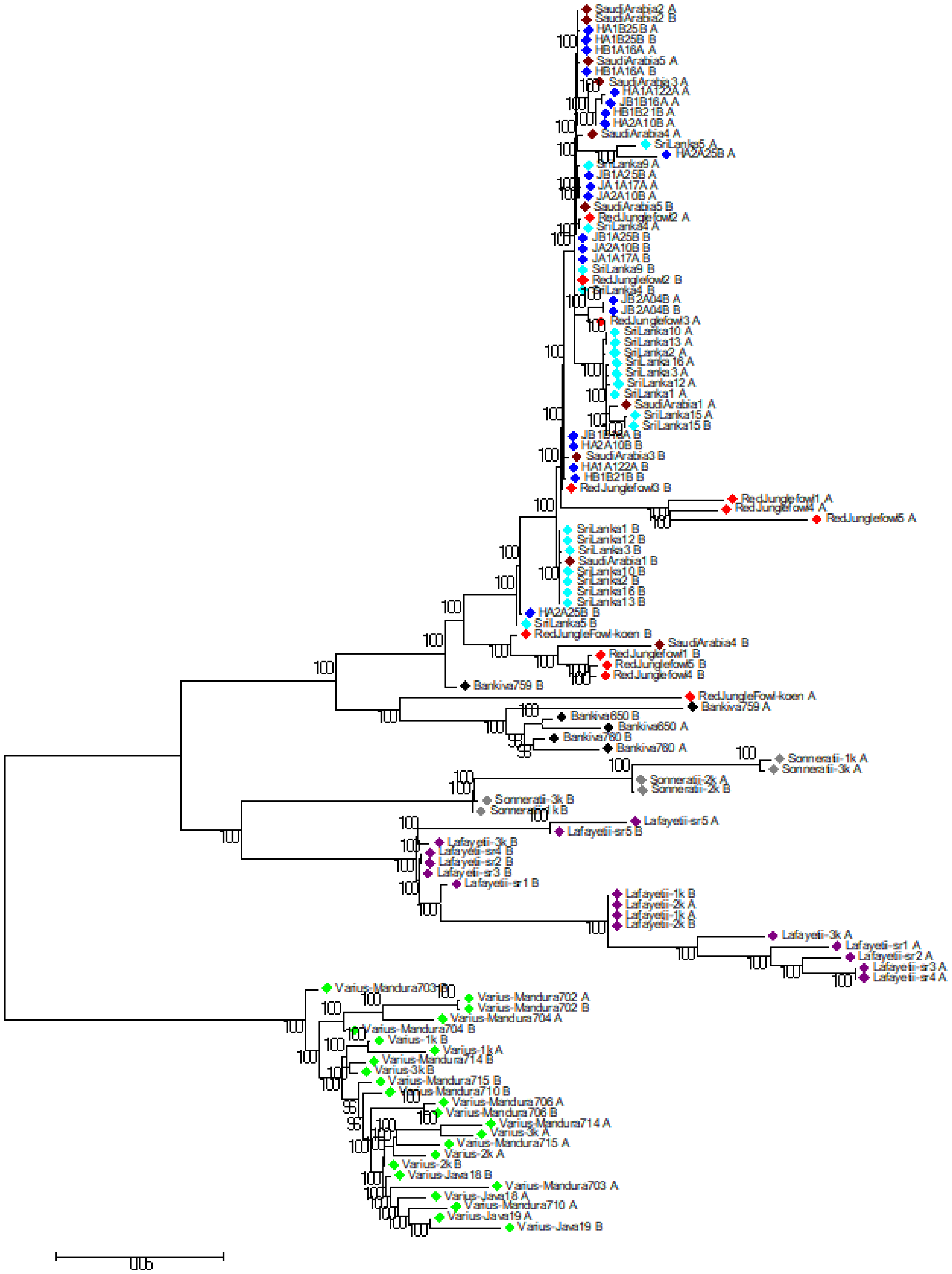

FIGURE S7 | A 40 kb candidate sweep region on chromosome 5 (*Galgal* 5.0 position 41868268 - 41908264) shared between the four domestic chicken population. ◆: Ethiopian chicken; ◆: Saudi Arabian chicken; ◆: Sri Lankan chicken; ◆: Red junglefowl; ◆: Javan red junglefowl; ◆: Grey junglefowl; ◆: Ceylon junglefowl; ◆: Green junglefowl
